# Assessment of Glyphosate Induced Epigenetic Transgenerational Inheritance of Pathologies and Sperm Epimutations: Generational Toxicology

**DOI:** 10.1038/s41598-019-42860-0

**Published:** 2019-04-23

**Authors:** Deepika Kubsad, Eric E. Nilsson, Stephanie E. King, Ingrid Sadler-Riggleman, Daniel Beck, Michael K. Skinner

**Affiliations:** Center for Reproductive Biology, School of Biological Sciences, Washington State University, Pullman, WA 99164-4236 USA

**Keywords:** DNA methylation, Diseases

## Abstract

Ancestral environmental exposures to a variety of factors and toxicants have been shown to promote the epigenetic transgenerational inheritance of adult onset disease. One of the most widely used agricultural pesticides worldwide is the herbicide glyphosate (N-(phosphonomethyl)glycine), commonly known as Roundup. There are an increasing number of conflicting reports regarding the direct exposure toxicity (risk) of glyphosate, but no rigorous investigations on the generational actions. The current study using a transient exposure of gestating F0 generation female rats found negligible impacts of glyphosate on the directly exposed F0 generation, or F1 generation offspring pathology. In contrast, dramatic increases in pathologies in the F2 generation grand-offspring, and F3 transgenerational great-grand-offspring were observed. The transgenerational pathologies observed include prostate disease, obesity, kidney disease, ovarian disease, and parturition (birth) abnormalities. Epigenetic analysis of the F1, F2 and F3 generation sperm identified differential DNA methylation regions (DMRs). A number of DMR associated genes were identified and previously shown to be involved in pathologies. Therefore, we propose glyphosate can induce the transgenerational inheritance of disease and germline (e.g. sperm) epimutations. Observations suggest the generational toxicology of glyphosate needs to be considered in the disease etiology of future generations.

## Introduction

Glyphosate (N-(phosphonomethyl)glycine) was discovered in 1950 and was commercialized for its herbicidal activity as Roundup in the 1970s by Monsanto, St. Louis Missouri^1^. Glyphosate is the world’s most commonly used herbicide accounting for nearly 72% of global pesticide usage^1^. It is the primary herbicide used in the agriculture of corn, soy, and canola, with extensive use in the USA, Supplemental Figure S1. The current “safe” standard set by the Environmental Protection Agency (EPA) for daily chronic reference dose of glyphosate is 1.75 milligrams per kilogram of body weight^1^. The no observable adverse effect level (NOAEL) is 50 mg/kg per day dose^2^. The allowed industry exposure levels are 2.5–4.5 mg/kg per day^2^. High exposure dose studies involving 50–500 mg/kg per day have been reported^3–9^. According to the European Food Safety Authority (EFSA) there is low acute toxicity observed by oral, dermal, or inhalation routes^10^. Glyphosate acts by inhibiting the 5-enolpyruvylshikimate-3-phosphate synthase (EPSP) enzyme that is involved in the metabolism of aromatic amino acids in plants. This inhibition leads to a protein shortage and eventually plant death^11^. The absence of this biochemical pathway in vertebrates, a rapid metabolism, and elimination of glyphosate in mammals (~5–10 hr half-life) has led to the assumption that low levels of toxicity are expected in humans and other mammals^2,10,11^.

There are many conflicting reports regarding the toxicity of glyphosate^12,13^. In March 2015 the International Agency of Research on Cancer classified glyphosate as a Grade 2a carcinogen based on prevalence of liver and kidney tumors in chronic feeding studies^1^. Shortly after, this statement was retracted in 2016^14^. Previous reviews of the literature have discussed the opposing opinions and the scientific studies involved^1,12,13^. Epidemiology studies have suggested direct exposure associations with diseases such as autism^15^, but appropriate animal or clinical studies have not been performed. A wide variety of different organisms have been used to assess the ecotoxicological actions of glyphosate^16^. Direct glyphosate exposure studies in mammals (i.e. mice and rats) have suggested a variety of different pathologies. A complicating factor is Roundup contains additional compounds along with glyphosate, but the actions are presumed to be only through glyphosate. Glyphosate, or Roundup, direct exposure has been linked to reproductive toxicity, birth defects^17^, reduced sperm production^18,19^ in rodents, and increased risk of liver metabolic pathologies^4^. Testis pathologies develop following direct glyphosate exposure involving Leydig cell, Sertoli cell and spermatogenic cell apoptosis and damage, as well as reduced testosterone production^20^. Additional male reproductive abnormalities include delayed onset puberty, behavioral alterations, and testis pathology^21–24^. Glyphosate induced female pathologies involve uterine abnormalities, altered ovarian steroidogenesis, and implantation pathology^25–27^ in rodents. A review of a small number of human epidemiological studies involving direct exposure to glyphosate concluded no risk for human development and reproduction^28^. Therefore, a mixture of studies exist showing no direct exposure effects versus induced pathologies. An increasing number of recent published studies suggest a potential risk of direct glyphosate exposure^1,12,13^. Regulatory agencies consider the herbicide to be minimally or not toxic^1,10^. The published literature has been focused on the direct exposure of an individual to glyphosate which is the primary current standard for toxicology risk assessment studies. No previous studies have examined the potential transgenerational impacts of glyphosate on successive generations not having continued direct glyphosate exposure.

Epigenetic transgenerational inheritance involves the germline (sperm or egg) mediated inheritance of epigenetic information between generations that leads to pathologies or phenotypic variation in the absence of continued direct exposures^29–31^. Epigenetics is defined as “molecular factors and processes around DNA that regulate genome activity (e.g. gene expression) independent of DNA sequence, and that are mitotically stable”^29^. The epigenetic processes include DNA methylation, histone modifications, non-coding RNA, chromatin structure, and RNA methylation. This non-genetic form of inheritance allows environmental factors to induce epigenetic alterations at critical developmental periods in the germline (sperm or egg) which can then be passed to subsequent generations^29,30^. These critical developmental periods involve the epigenetic reprogramming that occurs in the early embryo following fertilization^32^, and the reprogramming in the primordial germ cells in early gonadal development^33^. During adult gametogenesis, in particular spermatogenesis in the testis, epigenetic programming can also be altered^34^. Preconception adult exposures have also been shown to promote the transgenerational inheritance of pathologies^29,35^. Previous studies with a number of environmental toxicants have been shown to induce the transgenerational inheritance of pathologies, disease and sperm epigenetic alterations. This includes the fungicide vinclozolin^36–38^, plastic derived compounds (bisphenol A and phthalates)^39^, pesticides permethrin^40^, dichlorodiphenyltrichloroethane (DDT) and methoxychlor^41,42^, hydrocarbons (jet fuel JP8)^43^, dioxin^44^, and herbicide atrazine^45^. In addition to environmental toxicants, nutrition and stress can also promote the transgenerational inheritance of pathologies^29,30^. Human studies have also demonstrated epigenetic transgenerational inheritance in responses to nutrition, smoking, stress, and other environmental exposures^31^. Environmentally induced transgenerational inheritance of pathologies and phenotypic variations have been shown in plants, worms, flies, fish, birds, rodents, pigs, and humans^29^. Therefore, the environmentally induced epigenetic transgenerational inheritance phenomenon is induced by a wide variety of toxicants and environmental factors, and appears to be a highly conserved non-genetic inheritance process. The current study examines the influence of glyphosate on the transgenerational inheritance of pathologies and sperm epimutations.

## Results

Analysis of the transgenerational actions of glyphosate used outbred Sprague Dawley female rats (F0 generation) transiently exposed (25 mg/kg body weight glyphosate daily) during days 8 to 14 of gestation. This is half the NOAEL exposure of 50 mg/kg/day^10^, and due to rapid metabolism turnover would lead to a decreased (5–10 mg/kg) dose during the transient exposure period. The F1 generation animals (direct fetal exposure) were bred within the lineage to generate the F2 generation (direct germline exposure), which were bred to generate the F3 generation (transgenerational, no direct exposure). A control lineage used F0 generation gestating females administered vehicle control dimethyl sulfoxide (DMSO) or phosphate buffered saline (PBS). The control and glyphosate lineages were aged to 1 year and euthanized for pathology and sperm epigenetic analysis. No sibling or cousin breeding (crosses) was used in order to avoid any inbreeding artifacts in either the control or glyphosate lineages. Generally, 6–8 founder gestating females from different litters were bred, and 5 animals of each sex from each litter used to generate 25–50 individuals of each sex for each generation for analysis, as previously described^41^. Therefore, litter bias was negligible, and the full spectrum of pathology within the generation and lineage was assessed.

### Pathology Analysis

Upon dissection at one year of age, abdominal and thoracic organs were briefly examined for obvious gross abnormalities and pathologies. No pathologies or remarkable abnormalities were observed with the exceptions of some animals showing enlarged roughened kidneys (associated with histological evidence of renal disease), and some female animals showing enlarged fluid-filled uteri. All animals that died or were euthanized for welfare reasons prior to 1 year of age were submitted for necropsy and examined for gross and histologic pathologies by the Washington Animal Disease Diagnostic Laboratory (WADDL) at Washington State University College of Veterinary Medicine. For the eleven animals so submitted there were three F3 generation glyphosate control rats that showed aspiration pneumonia, dermal necrosis, or hepatic centrolobar necrosis. There were four F2 generation glyphosate lineage rats showing metritis, dystocia, hepatic necrosis, or adrenal cortical necrosis. There were four F3 generation glyphosate lineage rats showing granulomatous furunculosis, ulcerative balanoposthitis, or seizures for which the underlying diagnosis was open.

**Figure 1:**
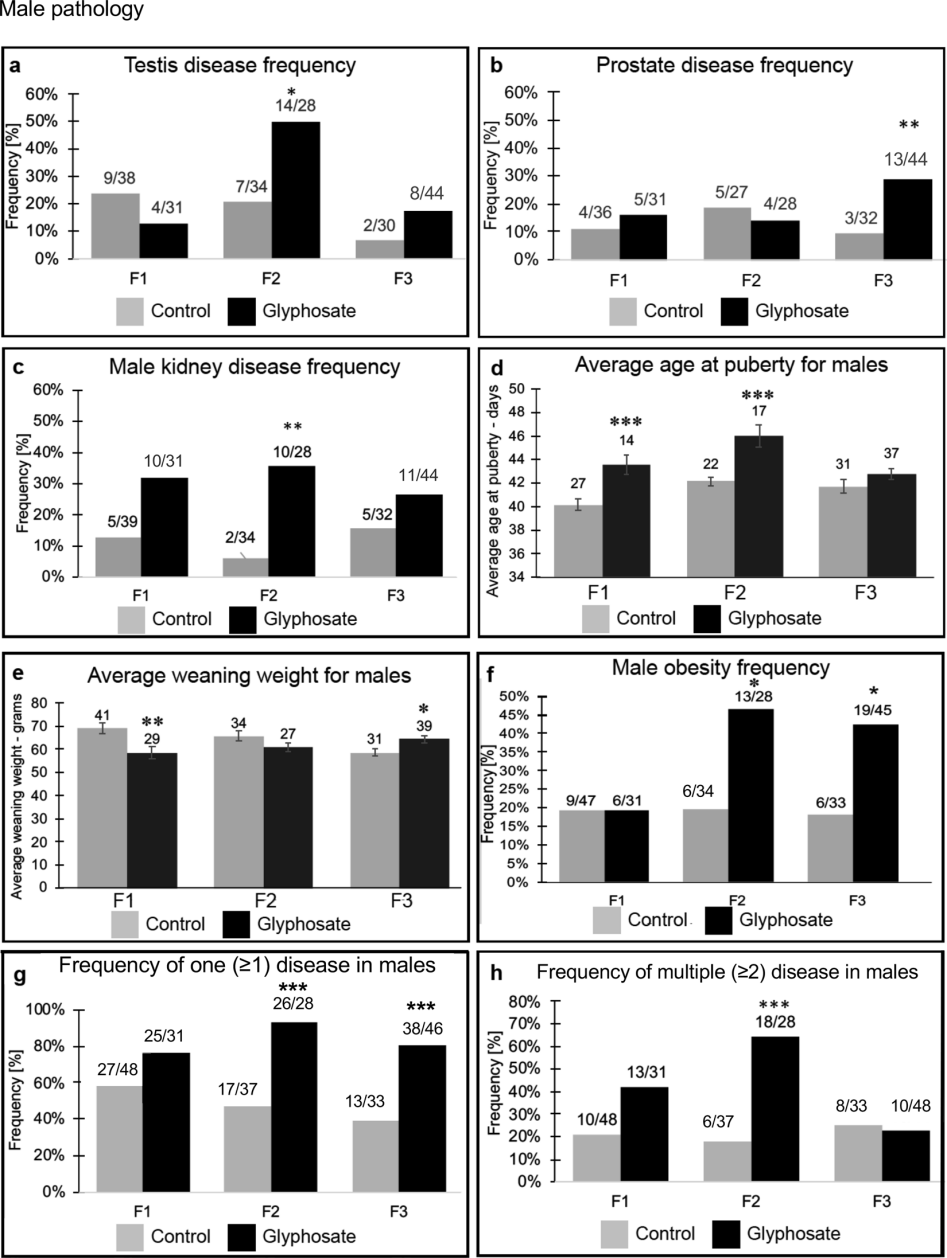
Male pathology analysis in F1, F2 and F3 generation control and glyphosate linage 1 year-old rats. **(a)** testis disease frequency, **(b)** prostate disease frequency, **(c)** male kidney disease frequency, **(d)** average puberty age for males, **(e)** average weaning weight for males, **(f)** male obesity frequency, **(g)** frequency of one disease in males, and **(h)** frequency of multiple disease in males. The pathology number ratio with total animal number is listed for each bar graph (**a**–**f**), or mean ± SEM (**d**,**e**), presented with asterisks indicating a statistical difference (*)p < 0.05, (**)p < 0.01, and (***)p < 0.001 in comparison with control lineage animals.

**Figure 2:**
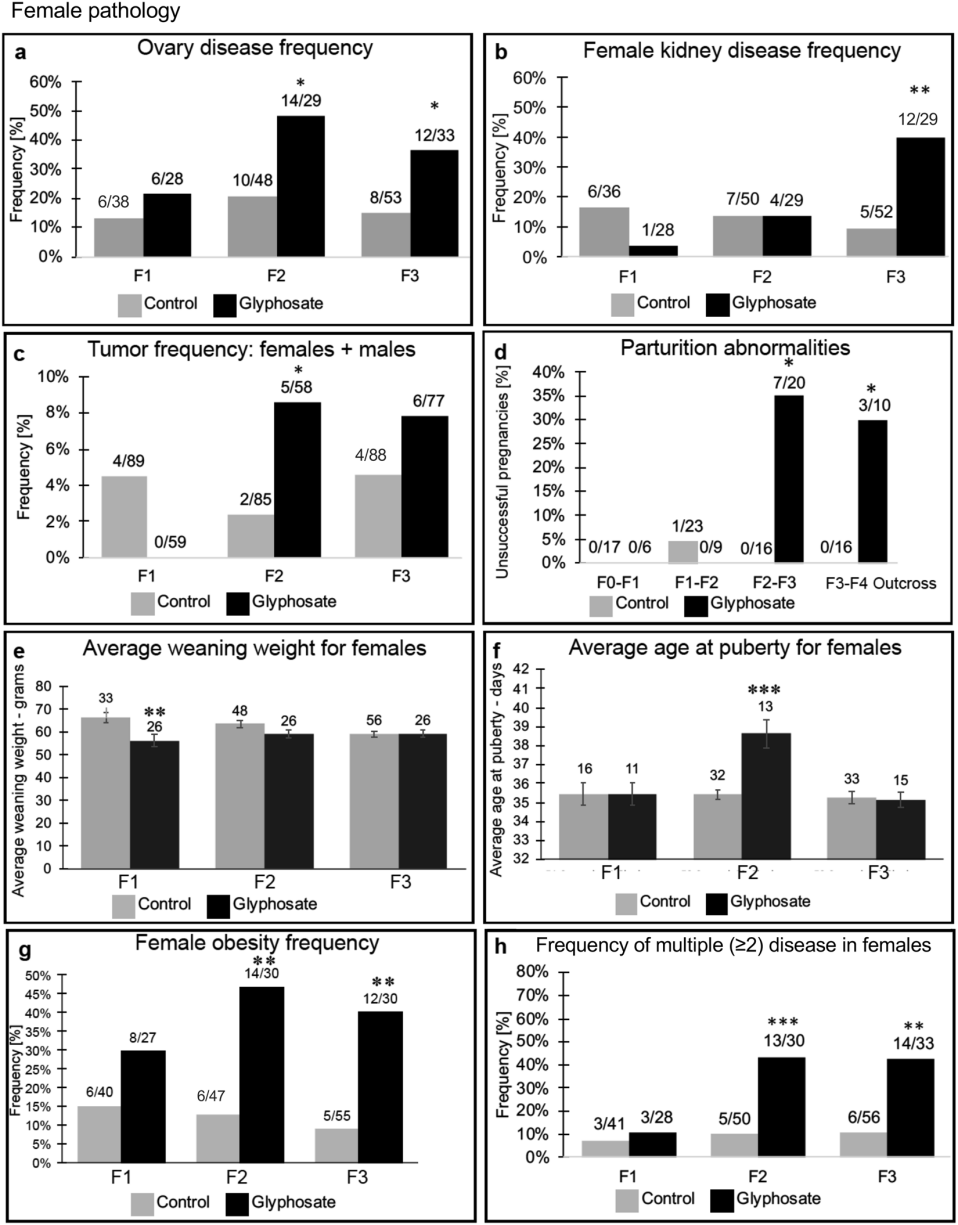
Female pathology analysis in F1, F2 and F3 generation control and glyphosate linage 1-yearr old rats. **(a)** ovary disease frequency, **(b)** female kidney disease frequency, **(c)** tumor frequency (males and females), **(d)** parturition abnormalities, **(e**) average weaning weight for females, **(f)** average age of puberty for females, **(g)** female obesity frequency, and **(h)** frequency of multiple disease in females. The pathology number ratio with total animal number is listed for each bar graph or mean ± SEM (**e**,**f**), presented with asterisks indicating a statistical difference (*)p < 0.05, (**)p < 0.01, and (***)p < 0.001 in comparison with control lineage animals.

Upon dissection at 1 year of age the testis, prostate, kidney, and ovary were collected and examined for histopathologies, Supplemental Figure S2. Stained paraffin sections of isolated tissues were examined by three different trained pathology observers blinded to the exposure lineages to assess the presence of specific histological abnormalities as described in the Methods^29^, (Supplemental Figure S2). The male and female pathologies are summarized in Figs 1 and 2, respectively, with the diseased individuals per total number of individuals presented for each generation and lineage, Supplemental Tables S1–S3. For the purposes of this paper an animal was considered to have a diseased tissue if the number of histological abnormalities was markedly increased (i.e. greater than two standard deviations) compared to that of the controls for that tissue, as described in Methods. Previously we have confirmed with apoptosis analysis an increase in spermatogenic cell death in testes^36,37^. Testis disease was characterized by the presence of histopathologies including azoospermia, atretic seminiferous tubules, presence of vacuoles in basal regions of the seminiferous tubules, sloughed germ cell in the lumen of tubules, and lack of tubule lumen^29^, (Supplemental Figure S2). The most common histology abnormalities were atrophy and vacuoles, followed by sloughed cells and debris in the tubule lumen. The frequency or incidence of testis disease was found to be significantly elevated in the F2 generation glyphosate lineage, but no effect was observed in the direct exposure F1 generation or transgenerational F3 generation at one year of age, Fig. 1a. The different mechanisms and exposures for each generation (F1 generation direct somatic exposure, F2 generation direct germline exposure and F3 generation no exposure) can generate distinct pathologies for each generation^29^. No gross abnormalities were observed in the corresponding epididymis at the time of dissection.

Prostate disease was characterized by atrophic or hyperplastic prostate glandular epithelium, and the presence of vacuole spaces in the epithelium as previously described^46^ (Supplemental Figure S2). The most common histological abnormalities were epithelial cell atrophy and vacuoles, followed by hyperplasia. The prostate atrophy and regions with the presence of vacuoles are generally distinct from the regions showing hyperplasia. The frequency or incidence of prostate disease was similar for the control and glyphosate F1 and F2 generation males at one year of age, Fig. 1b. Interestingly, there was an increased frequency of prostate disease observed in the F3 generation glyphosate lineage males (p < 0.01). Therefore, one of the transgenerational pathologies (F3 generation) observed was prostate disease in approximately 30% of glyphosate lineage males, a three-fold increase in disease rate over controls, Fig. 1b.

Kidney disease was characterized by the presence of an increased number of proteinaceous fluid filled cysts, reduction in size of glomeruli, and thickening of Bowman’s capsules, as previously described^37,47^ (Supplemental Figure S2). The most common histological abnormalities were cysts, presumably derived from dilated tubules, and thickened Bowman capsules, followed by reduced glomerular areas. There was an increase in kidney disease frequency in the glyphosate lineage males in the F2 generation, but not F1 or F3 generations, Fig. 1c. The frequency of kidney disease was found to be similar for both the F1 and F2 generation between the control and the glyphosate lineage females. There was an increased incidence of kidney disease observed in the F3 generation glyphosate lineage females affecting nearly 40% of females (a four-fold increase in disease rate) compared to the F3 generation control females, Fig. 2b.

Ovarian disease was characterized by the development of polycystic ovaries with an increase in the number of small and large cysts showing negligible granulosa cells, as previously described^48^ (Supplemental Figure S2). The most common histological abnormalities were small cysts followed by large cysts. In addition, follicle counts were performed to determine any changes in the primordial follicle pool size, as previously described^48,49^. The frequency of ovarian disease was not significantly different between control and glyphosate lineages in the F1 generation. However, there was a significant increase in ovarian disease observed in the F2 and F3 generation glyphosate lineage females when compared to the control lineage, Fig. 2a.

Tumor development was also monitored in males and females, and found to increase in the F2 generation glyphosate female lineage, but not the F1 or F3 generation glyphosate lineages, Fig. 2c. The most predominant tumors to develop in the male and female were mammary adenomas, as previously described^37,41^. One mammary fibrosarcoma, one lymphoma, one trichoepithelioma and one aural fibrosarcoma were also identified. Tumor histopathology analysis was performed by WADDL.

Pubertal analysis revealed delayed pubertal onset in males in the F1 and F2 generation glyphosate lineage, but no effects in the F3 generation, Fig. 1d. Female pubertal onset was delayed in the F2 generation glyphosate lineage, and no effects were observed in the F1 or F3 generations, Fig. 2f.

Analysis of potential direct fetal exposure toxicity effects of glyphosate in the F1 generation and subsequent F2 and F3 generations included evaluation of litter sizes, sex ratios and weaning body weights of pups. There was no effect on litter size or sex ratio observed for any generation, Supplemental Figure S3. There was significantly lower weaning body weight observed for the F1 generation in the glyphosate lineage for both males (p < 0.01), Fig. 1e, and females (p < 0.01), Fig. 2e. In the F3 generation, there was no statistical difference in weaning weights for females, but an increase in males between the control and glyphosate lineages (Figs 1e and 2e).

A parturition (birth) abnormality was observed, and involved either the death of the late stage gestating mother or her pups immediately after or during birth. This phenotype was not observed in the F0 generation breeding to produce the F1 generation in either the glyphosate or control lineages. In F1 breeding to produce F2 generation offspring there was one instance in the control population where a parturition abnormality was observed, and no such instance occurred in that generation in the glyphosate lineage, Fig. 2d. However, during the gestation of F2 generation mothers with the F3 generation fetuses, dramatic parturition abnormalities were observed in the glyphosate lineage. The frequency of unsuccessful parturition was 35% (p < 0.03). Out of the 7 cases that were classified as unsuccessful pregnancies, Fig. 2d, there were 5 maternal mortalities observed. Necropsy of these animals by WADDL diagnosed 2 cases of dystocia, 1 case of severe rhinitis, 1 case of adrenal gland necrosis, and 1 case in which the cause was unknown. To further investigate the parturition abnormalities an outcross of F3 generation glyphosate lineage males with a wildtype female was performed. There were parturition abnormalities observed with a frequency of 30% (p < 0.04), Fig. 2d. In the paternal outcross generation, there were 3 cases of maternal mortality. The causes of maternal death confirmed by WADDL were 2 cases in which the cause of death was identified as dystocia, and 1 case of hyperplasia-mastitis. In order to quantify the rates of initial successful pregnancies, fertility rates of the females were compared between the control and the glyphosate lineages. Fertility rate was defined as the number of pregnancies divided by the number of breedings. Results showed no significant difference in the comparison of glyphosate and control lineage fertility rates in any generation, Supplemental Figure S3a.

The weight, body mass index (BMI), abdominal adiposity, and adipocyte cell size were analyzed in order to assess the frequency of obesity in glyphosate and control lineage males and females, as described in the Methods^45^ (Supplemental Figure S2). Analysis of potential obese phenotypes in the F1, F2, and F3 generation glyphosate and control lineages identified a significant increase in the obese phenotype of the F2 and F3 glyphosate lineage males and females, Figs 1f and 2g. The frequency of obesity was not found to be different between the control versus glyphosate lineage F1 generation males and females. Therefore, a transgenerational (F3 generation) obese phenotype was observed in approximately 40% of the glyphosate lineage females and 42% of the glyphosate lineage males, Figs 1f and 2g.

Direct exposure studies to glyphosate have been shown to induce behavioral abnormalities in the exposed F0 generation^50–53^. Behavioral analysis of the glyphosate and control lineage transgenerational F3 generation at 11 months of age was done. Both a light and dark box (LDB) and elevated plus maze (EPM) were used to assess potential anxiety behavior^50^. The F3 generation glyphosate lineage males and females had fewer total light side attempts and fewer total attempts compared to the controls in the light and dark box, Supplemental Figure S4a–e. No changes in other light and dark box parameters were observed. For the elevated plus maze with an open and closed arm results indicate that there was no behavioral difference observed (p > 0.05) for the control or glyphosate (open arm time or closed arm time per total time ratio) lineage F3 generation females or males, Supplemental Figure S4g and i. None of the other parameters of the EPM analysis were found to be altered, Supplemental Figure S4f–j. Although there was a reduced number of light and total attempts in the LDB by the glyphosate lineage F3 generation males, none of the other LDB or EPM parameters supported a behavioral effect, Supplemental Figure S4. Therefore, no major behavioral effects were observed in the F3 generation glyphosate lineage males or females.

The incidence of disease and abnormalities in all F1, F2 and F3 generation control and glyphosate lineage males and females is presented in Figs 1 and 2 and Supplemental Tables S1–S3 (a–d). The specific diseases associated with each individual animal are shown in Tables S1 (a–d), S2 (a–d) and S3 (a–d). This information was used for the analysis of one (≥1) disease and multiple (≥2) disease incidence, Figs 1 and 2. The frequency of one (≥1) disease in F1, F2 or F3 generation glyphosate lineage females was not statistically different from control lineage animals. In males, the frequency rate of one (≥1) male disease did not differ from the controls in the F1 generation, but increased significantly in the F2 and F3 generations, Fig. 1g. The frequency of multiple diseases (≥2) for females was not significant for the F1 generation, but the frequency increased for glyphosate lineage females in the F2 generation (p < 0.01) and F3 generation (p < 0.01) (Fig. 2h). Over 40% of the F3 generation glyphosate lineage females (2-fold increase) developed disease and abnormalities when compared to the controls. The frequency of multiple disease in the F1 and F3 generation males was not statistically different from controls, but an increase in multiple disease frequency was observed in the F2 generation males (p < 0.01) (Fig. 1h). Therefore, the F3 generation glyphosate lineage females had a significant increase in multiple diseases, suggesting a transgenerational increase in disease susceptibility.

### Sperm Epigenetic Analysis

**Figure 3:**
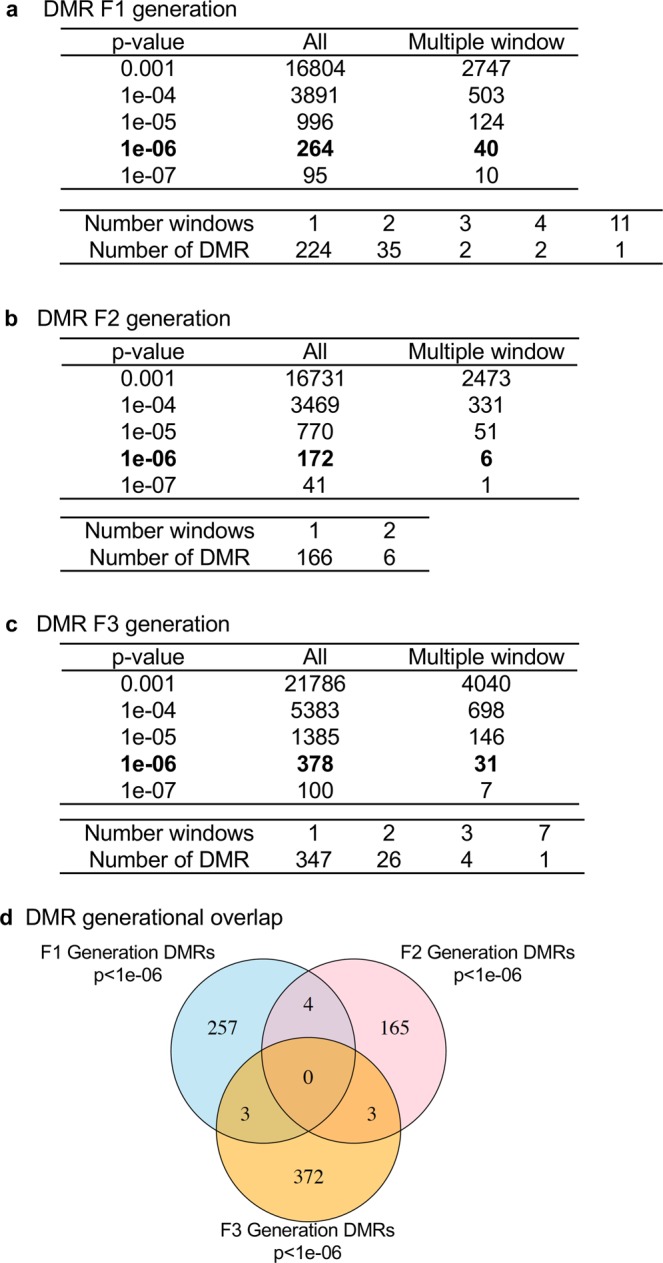
Epigenetic analysis and DMR identification. The number of DMRs found using different p-value cutoff thresholds. The All column shows all DMRs. The Multiple Window column shows the number of DMRs containing at least two significant windows. The number of DMR with each specific number of significant windows at a p-value threshold of 1e-06 is shown below each table. **(a)** DMR F1 Generation. **(b)** DMR F2 Generation. **(c)** DMR F3 Generation. **(d)** DMR overlap Venn diagram.

Glyphosate induced transgenerational inheritance of disease and pathology requires the germline (sperm or egg) transmission of epigenetic information between generations^29^. Therefore, sperm was collected from the control and glyphosate lineage F1, F2 and F3 generation males for epigenetic analysis. Potential differential DNA methylation regions (DMRs) in the sperm were identified using a comparison between the control and glyphosate lineage, as described in the Methods^45^. The sperm DNA was isolated, fragmented and the methylated DNA immunoprecipitated (MeDIP) with a methyl-cytosine antibody. The MeDIP DNA fragments were sequenced for an MeDIP-Seq analysis as described in the Methods^54^. The sperm DMR numbers are presented in Fig. 3 for a variety of p-value cutoff thresholds, and p < 10^−6^ was selected as the threshold for all subsequent analyses. The total number of DMRs for the control versus glyphosate lineage F1 generation is 264 with 40 of them having multiple neighboring 100 bp windows, Fig. 3a. The F2 generation had 174 DMRs with 6 of them with two multiple windows detected, Fig. 3b. The transgenerational F3 generation sperm were found to have 378 total DMRs with 31 of these having multiple neighboring windows, Fig. 3c. Therefore, the glyphosate lineage sperm were found to have altered DNA methylation in direct exposure F1 and F2 generations, as well as the transgenerational F3 generation^55^. Interestingly, there was negligible overlap of the sperm DMRs between each generation, Fig. 3d. Previous studies have observed that direct exposure and transgenerational generation DMRs are distinct, apparently due to the unique mechanisms for direct exposure toxicity and transgenerational actions of environmental exposures^29,55^. Observations indicate glyphosate can promote germline epigenetic alterations in DNA methylation.

**Figure 4:**
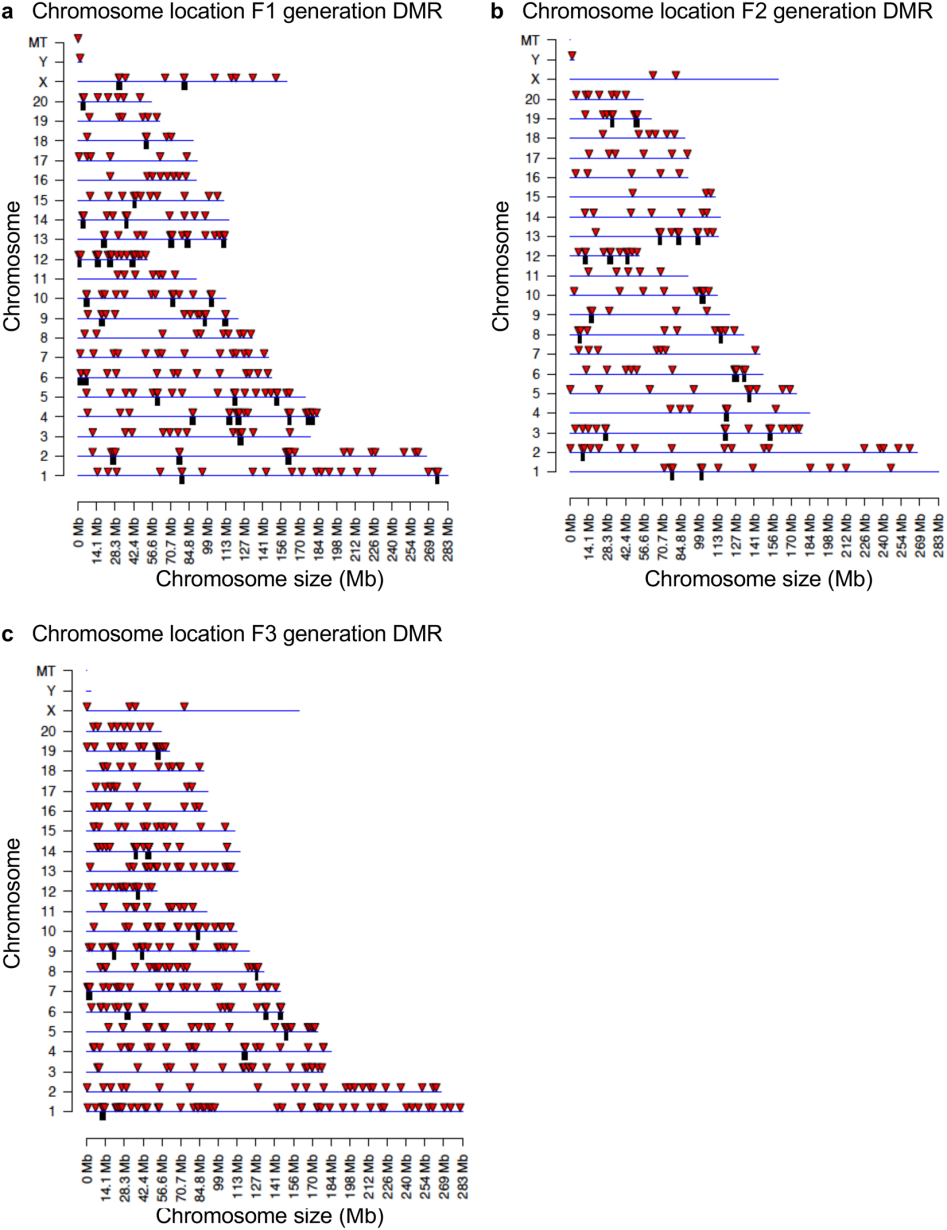
Chromosomal location of DMRs at a p-value threshold of 1e-06. **(a)** Chromosome locations of F1 generation DMRs. **(b)** Chromosome location F2 generation DMRs. **(c)** Chromosome location F3 generation DMRs. Triangles indicate DMRs. Rectangles indicate clusters of DMRs.

**Figure 5:**
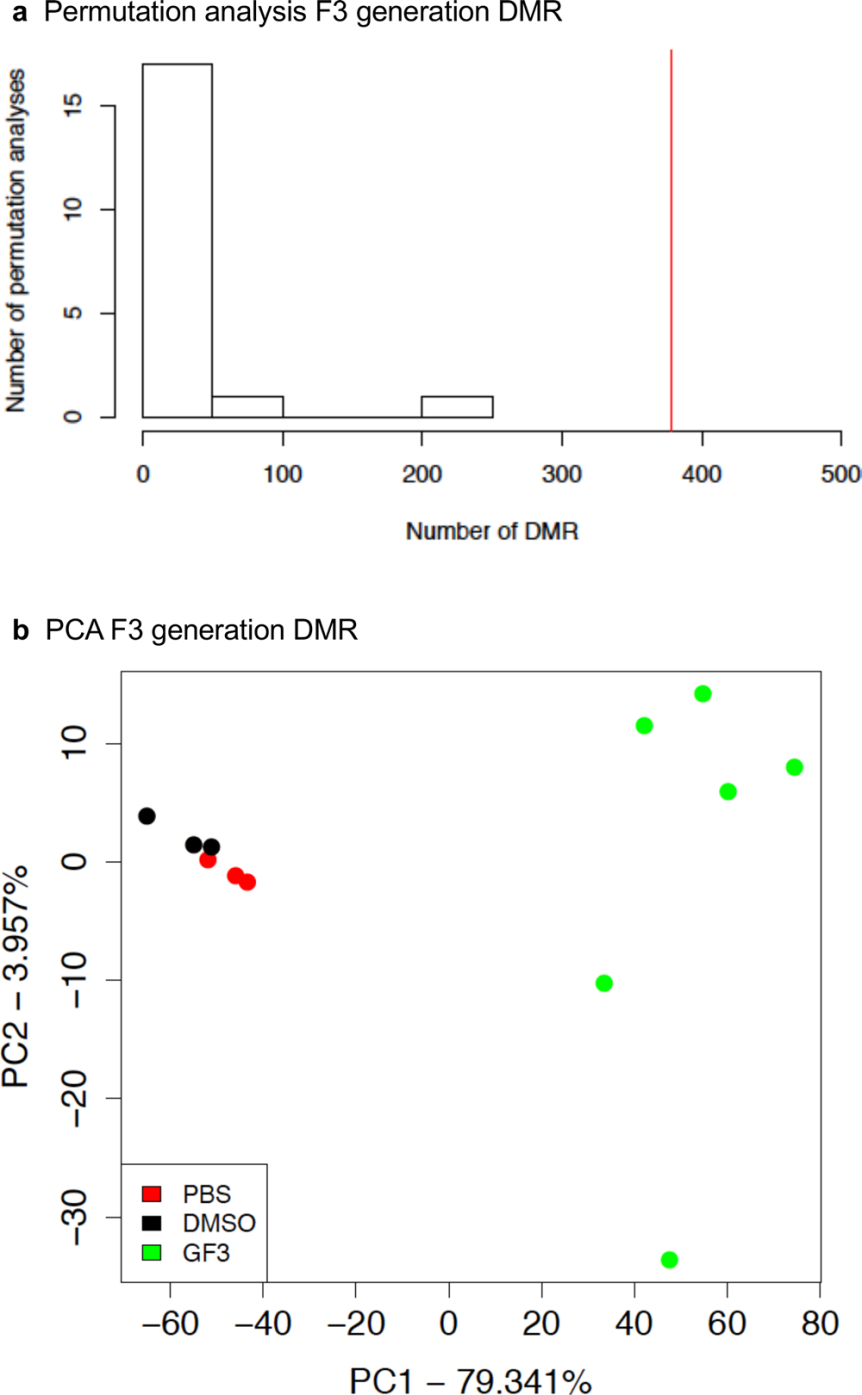
Permutation and principle component DMR analysis (PCA). **(a)** The number of F3 generation DMR for all permutation analyses. The vertical red line shows the number of DMR found in the original analysis. All DMRs are defined using an edgeR p-value threshold of 1e-06. **(b)** DMR PCA using DMRs and not whole genome for F3 generation control and glyphosate DMR analysis with legend insert. The control (PBS), control (DMSO) and glyphosate F3 generation lineage (GF3) indicated.

The chromosomal locations of the DMRs for each generation are presented in Fig. 4. Nearly all chromosomes had DMRs for the F1, F2 and F3 generations, indicated by arrowhead, along with clusters of DMRs indicated by black boxes, Fig. 4. Therefore, the DMR identified were genome-wide on all chromosomes. The genomic features of the DMRs were investigated and shown to have a low CpG density “CpG deserts”^56^, and be predominantly 1 kb in length, Supplemental Figure S5. Similar DMR genomic factors were observed for the F1, F2 and F3 generations. The F3 generation DMR data was used in a permutation analysis to show the number of DMRs identified (red line) is not due to random variation in the control and glyphosate data, Fig. 5a, and correlated with the false discovery rate (FDR) analysis performed. In addition, a principle component analysis (PCA), with the DMRs not whole genome, of the F3 generation DMRs showed efficient separation of the control versus glyphosate DMR data and clustering of control DMR data, Fig. 5b. Similar observations were made with the F1 and F2 generation DMR PCA analysis, Supplemental Figure S6. These data demonstrate that statistically significant DMRs are observed for the F1, F2 and F3 generations sperm.

**Figure 6:**
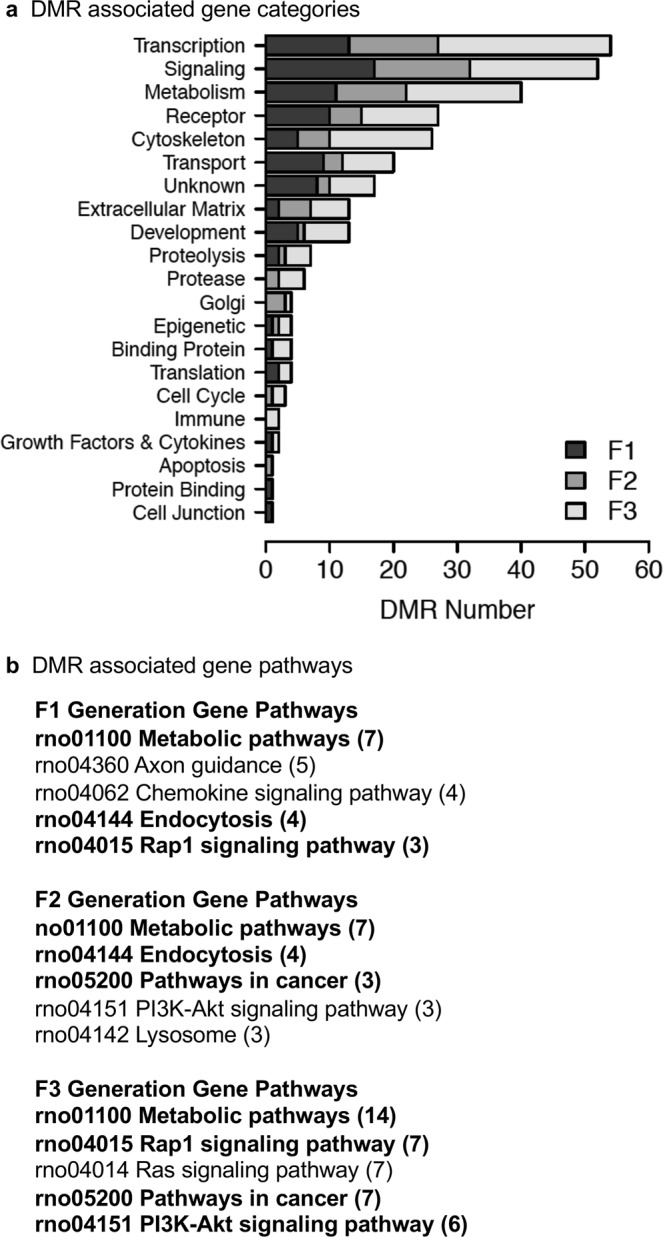
DMR gene associations. **(a)** DMR associated gene functional categories (genes within 10 kb DMR). **(b)** KEGG pathways containing DMR associated genes. Number DMR associated genes in pathway in brackets. Pathways in bold are common to at least two generations.

The DMR associated genes were identified for DMRs within 10 kb of a gene to include gene promoters and listed in Supplemental Figures S4, S5 and S6 for the F1, F2 and F3 generation DMR lists, respectively. The majority of DMRs were not associated with genes. The genes and associated gene categories for each DMR and associated genes are provided. A summary of the DMR associated gene categories indicates transcription, signaling, metabolism, receptors, and cytokines are predominant, Fig. 6a. A summary of DMR associated gene categories are presented for the F1, F2 and F3 generation gene categories. The DMR associated gene pathways are presented in Fig. 6b. The top five KEGG (Kyoto Encyclopedia of Genes and Genomes) gene pathways for the F1, F2 and F3 generations are listed with number of DMR associated genes involved in the pathway shown in brackets. The only pathway that overlaps in all three generations is the metabolic pathway, but this pathway involves hundreds of genes and sub-pathways so is anticipated. Other common pathways between the F1 and F2 generations and F2 and F3 generations are present. Various signaling pathways are the most common pathways identified.

## Discussion

**Figure 7:**
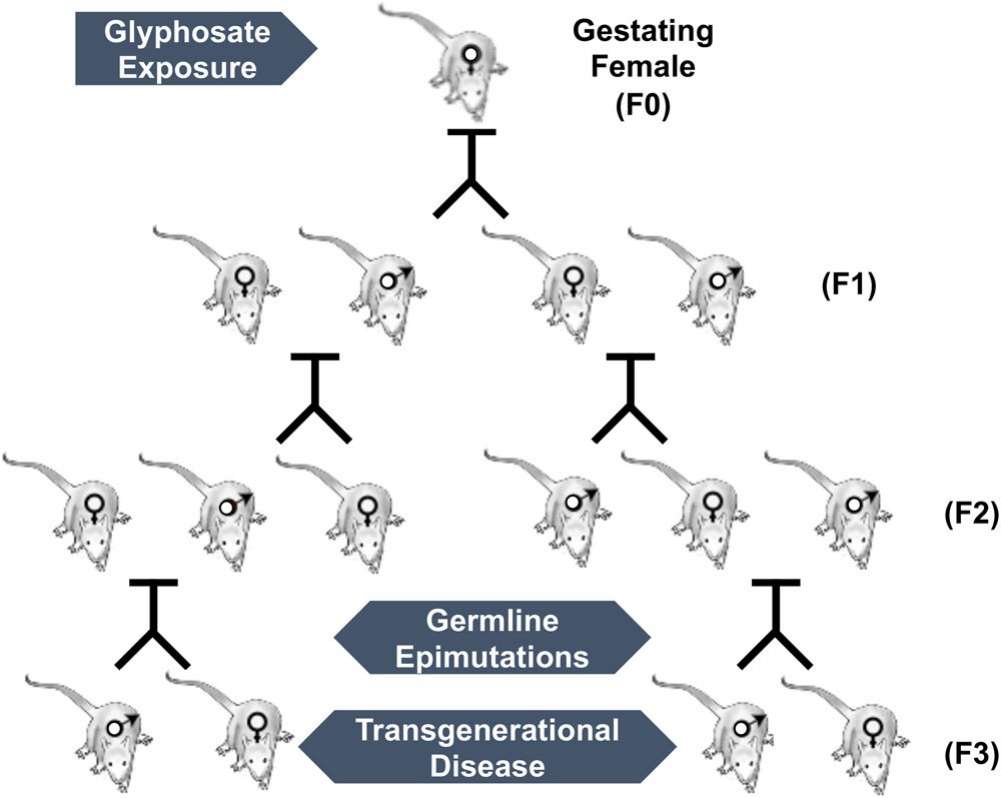
Schematic of generational (F0, F1, F2 and F3) transmission through male and female lineage to the transgenerational F3 generation.

Glyphosate is the most commonly used pesticide worldwide with predominant use in corn, soy and canola crops, Supplemental Figure S1. Although there have been many reports regarding the potential toxicity of glyphosate^1,12,13^, direct exposure has been suggested by regulatory agencies to have minimal or no toxicity^10,11^. A recent study suggested glyphosate induced female reproduction abnormalities in the offspring of exposed rats^27^. The current study provides the first analysis of potential transgenerational impacts of glyphosate in mammals. The exposure of a gestating female directly exposes the F0 generation female, the F1 generation offspring, and the germline within the F1 generation offspring that will generate the F2 generation grand-offspring^45^. Therefore, the first transgenerational generation is the F3 generation great-grand-offspring not having any direct exposure^55^, Fig. 7. The direct exposure mechanisms of action in the F0, F1 and F2 generations are distinct from the transgenerational germline mediated actions. Although the F2 generations grand-offspring can have a mixture of direct exposure and generational actions^29^, the lack of any direct exposure is first observed in the transgenerational F3 generation, Fig. 7. The impacts of transient glyphosate exposure on a F0 generation gestating female and subsequent generations not receiving any further exposure were assessed. Preconception adult exposures (e.g. abnormal diet and mercury) of males and females have also been shown to promote transgenerational impacts^57,58^. The current study focused on gestating female exposure actions.

The impacts of environmental exposures on subsequent generations can be referred to as “Generational Toxicology”, and suggests ancestral exposures can promote the onset of disease and pathology in subsequent generations. The mechanism involved is epigenetic transgenerational inheritance through epigenetic alterations of the germline^29^. Although many exposures can influence both the directly exposed individuals and transgenerational individuals, recent observations suggest some toxicants or exposures have negligible impacts on the direct exposed individuals, but can influence subsequent generations never having direct exposure. For example, a recent study with the herbicide atrazine was found to have negligible impacts on the direct exposed F0, F1 or F2 generations, but increased pathologies in the transgenerational F3 generation^45^. Therefore, classic toxicology analysis with atrazine demonstrates negligible or low risk of direct exposure, so relative safety for the compound, since “Generational Toxicology” is not considered. The possibility glyphosate may have similar transgenerational actions was investigated.

Analysis of the direct actions of glyphosate demonstrated no overt toxicity on the F0, F1, F2 or F3 generations considering the lack of impacts on litter size, sex ratios or fertility, Figure S3. The F1 generation offspring had negligible pathologies in any of the tissues analyzed. The only effects observed were on weaning weights in both males and females, and a delay in puberty in males. Therefore, classic toxicology analysis of the F0 and F1 generations demonstrated negligible toxicity or pathology from direct glyphosate exposure. In contrast, the F2 generation grand-offspring, derived from a direct exposure F1 generation germline^29^, had significant increases in testis disease, kidney disease, obesity, and multiple diseases in males, Fig. 1. The F2 generation females had significant increases in ovary disease, obesity, mammary gland tumors, parturition abnormalities, and multiple disease susceptibility, Fig. 2. The transgenerational F3 generation great-grand-offspring males had increased prostate disease, obesity, and single disease frequencies, while females had increased ovarian disease, kidney disease, parturition abnormalities, and multiple disease susceptibility, Figs 1 and 2. A unique pathology observed with glyphosate exposure, and seldom seen in previous transgenerational studies^59^, was the parturition abnormalities. Over 30% of the F2 generation female rats in the later stages of gestation died of dystocia and/or had litter mortality. This was also seen in the paternal outcross F3 generation gestating female rats, Fig. 2. Although dystocia and parturition mortalities are not a common occurrence in humans today due to improved obstetric care, the underlying pathology observed may reflect parturition abnormalities such as premature birth rates and infant abnormalities seen today^60,61^. In addition, the significantly higher obesity rates observed in the F2 and F3 generation male and female rats appear to correlate with the dramatic increase in obesity in the human population observed over the past several generations^62^. Many of the pathology frequencies observed to be induced by glyphosate transgenerationally are similar to the frequency of pathology in the human population today, for example the 40% obesity frequency identified. In contrast to the negligible pathology and disease observed in the F0 and F1 generations, significant pathology was observed in the F2 generation and transgenerational F3 generation males and females. Therefore, based on the associations of glyphosate exposure, the transgenerational disease and sperm epigenetic alterations, we propose glyphosate promotes the epigenetic transgenerational inheritance of disease.

Interpretation of the data needs to take into consideration the experimental design and technical limitation of the study. The current study used a mode of administration to control the exposure dose that does not allow a classic risk assessment. The experimental dose used does provide an environmentally relevant exposure of twice the allowed industry exposure (2.8–5 mg/kg/day) following metabolism of the glyphosate and half the NOAEL. Therefore, the current study was performed to simply determine the potential that glyphosate may promote the epigenetic transgenerational inheritance of pathology and sperm epimutations. Observations suggest future glyphosate risk assessment will need to consider generational toxicology and transgenerational impacts. Classic and current toxicology studies only involve direct exposure of the individual, while impacts on future generations are not assessed. In addition, a technical limitation of the study to consider was the observation that a founder effect derived from one control lineage female and one control lineage male from the F0 generation, whose offspring when bred to the F2 generation resulted in nearly all offspring having obesity. This level of disease (100% in females) suggests an abnormality and founder effect. This was calculated and confirmed as outlined in Supplemental Figure S7, and resulted in the removal of all individuals from these control founders lineages. A replacement was made with a concurrently generated control population using PBS (phosphate buffered saline) versus DMSO (dimethyl sulfoxide) as vehicle control exposure. A principle component analysis (PCA) with the DMRs was performed on the PBS and DMSO controls in the epigenetic analysis (Fig. 5b and Supplemental Figure S6), and they clustered together with the F3 generation and to a lesser extent in the F1 and F2 generations. Separately, a histopathology analysis comparison of the PBS and DMSO controls did not find any statistical difference pathologies between the controls. In addition, analysis of the pathologies with the affected founders lineages present still identified similar generational pathologies with the exception of the frequency of kidney disease and obesity being reduced. Therefore, a founder effect was identified, and the replacement controls were shown to correlate well. Although potential founder effects for other pathologies were considered in the F1 generation controls and glyphosate lineages, no other founder effects were identified. Finally, rodent models have long been used as model organisms to study human related phenomena. Rodent animal studies should be considered relevant to humans due to the extensive evolutionary conservation of the vast majority of physiological systems among mammals. Although the specific physiological impact and pathologies may vary, the current study indicates transgenerational impacts need to be considered.

The molecular mechanisms involved in epigenetic transgenerational inheritance requires germline (sperm and egg) epigenetic alterations, termed “epimutations”^29^. A recent study identified the concurrent transgenerational alterations in DNA methylation, ncRNAs and histone retention have been observed in sperm^54^. Although the focus of the current study is to identify the presence of epimutations only involved DNA methylation analysis, other epigenetic processes are also anticipated to be involved. A comparison of control versus glyphosate lineage F1, F2 and F3 generation male sperm DNA identified DMRs at each generation. As previously demonstrated^54^, negligible overlap of DMRs were observed between the generations (Fig. 3d) which appears to reflect the very different mechanisms of action between direct exposure in the F1 and F2 generation versus the transgenerational F3 generation mechanisms (germline induced embryonic stem cell alteration)^29^. The genomic features of the DMRs were similar to those previously described involving CpG deserts^29^. DMR gene associations were determined and found to have common gene functional categories and some overlap in gene pathways between generations, but specific genes did not overlap.

Approximately ~43% of the F3 generation DMR are associated with genes, while the majority (57%) are intergenic. These intergenic regions have been proposed to influence associated disease functions^63^ in the regulation of ncRNA that can act distally across megabases^64,65^. The DNA methylation can also stabilize copy number variation and suppress transposons, and alter chromatin structure^66,67^. Therefore, the DMRs are proposed to be functional with some impacting gene expression^63^. The, sperm epimutations identified (i.e. DMRs) are proposed to in part mediate the transgenerational pathology phenotype observed.

Although the DNA methylation is correlated with the transgenerational pathology, and previous studies shown to have regulatory roles in germline inheritance mechanisms^29^, a functional role for DNA methylation remains to be elucidated. Interestingly, alterations in germline ncRNAs has been shown to be functionally linked and determined by injection of altered sperm ncRNA into eggs and observing the induced transgenerational phenotypes^30^. Further research is needed, but the current literature suggests a functional role for the altered germline epigenome in mediating transgenerational epigenetic inheritance. For example, a DNA methylation epigenetic alteration in a plant flowering phenotype has been shown to be epigenetically inherited for over a 100 generations^29,68^. Similar alterations in drosophila and C. elegans have also been reported^29,69–71^, but the epigenetic process is distinct and appears to involve histone alterations. Observations suggest the phenomenon does not appear easily reversible. Previous studies have shown that environmentally induced epigenetic transgenerational inheritance does not impact genetics of the F1 generation sperm, as shown by analysis of copy number variations (CNVs) or point mutations^72,73^, but genetic mutations start to appear in the F3 generation sperm^72,73^. Therefore, the integrated actions of epigenetics and genetics may contribute to the transgenerational phenotypes, but this remains to be elucidated.

The F1 generation offspring epigenetic alterations are due to direct exposure of specific somatic cell types^55^ and negligible pathology or disease was observed. The transgenerational F3 generation great-grand-offspring developed pathology and appears to involve the presence of germline (i.e. sperm) epimutations. The proposal is following fertilization the epigenetic alterations potentially alter the early developing stem cells in the embryo. This may result in epigenetic and transcription alterations in all somatic cell types and lineages derived from these embryonic stem cells^29^. We have observed this in the Sertoli cells in the testis^74^, in prostate epithelial cells^46^, and in granulosa cells in the ovary^75^. Therefore, the pathology and disease observed in the F3 generation appear to be in part the result of cell type specific epigenetic and transgenerational alterations for potentially all cells and tissues^29^. Glyphosate exposure of the F0 and F1 generation had negligible toxicity and pathology, which supports direct exposure having low risk, however, the transgenerational germline mediated inheritance promotes significant pathology and disease.

## Conclusions

In summary, glyphosate was found to promote the epigenetic transgenerational inheritance of disease and pathology through germline (i.e. sperm) epimutations. Negligible pathology was observed in the F0 and F1 generations, while a significant increase in pathology and disease was observed in the F2 generation grand-offspring and F3 generation great-grand-offspring. Therefore, glyphosate appears to have a low or negligible toxic risk for direct exposure, but promotes generational toxicology in future generations. Observations suggest generational toxicology needs to be incorporated into the risk assessment of glyphosate and all other potential toxicants, as previously described^45^. The ability of glyphosate and other environmental toxicants to impact our future generations needs to be considered, and is potentially as important as the direct exposure toxicology done today for risk assessment.

## Methods

### Animal studies and breeding

As previously described^76^, female and male rats of an outbred strain Hsd:Sprague Dawley^®™^SD^®™^ (Harlan) at 70 to 100 days of age were fed ad lib with a standard rat diet and ad lib tap water. Timed-pregnant females on days 8 through 14 of gestation^59^ were administered daily intraperitoneal injections of glyphosate (25 mg/kg BW/day dissolved in PBS) (Chem Service, Westchester PA) or dimethyl sulfoxide (DMSO) or Phosphate Buffered Saline (PBS), as previously described^47^. Twenty-five mg/kg for glyphosate is 0.4% of rat oral LD50 and 50% of the NOAEL and considering glyphosate rapid metabolism approximately twice the occupational exposure 3–5 mg/kg per daily exposure^10,77^. There was a founder effect observed in the offspring of a specific male from the control population treated with PBS, manifesting as abnormally high rates (80–100%) of obesity in descendants, Supplemental Figure S7. Therefore, a portion of original control colony was excluded from the study due to the obesity founder effects identified. These animals were replaced with offspring from DMSO-treated controls from a concurrent study. Disease phenotypes were compared from both DMSO lineage and PBS lineage controls, with no significant differences observed with histopathology evaluations between the two populations, Fig. 5 and Supplemental Figure S6.

As previously described^76^, the gestating female rats treated were designated as the F0 generation. F1–F3 generation control and glyphosate lineages were housed in the same room and racks with lighting, food and water as previously described^37,47,78^. All experimental protocols for the procedures with rats were pre-approved by the Washington State University Animal Care and Use Committee (protocol IACUC # 6252). All methods were performed in accordance with the relevant guidelines and regulations.

#### Tissue harvest and histology processing

Rats were euthanized at 12 months of age by CO_2_ inhalation and cervical dislocation for tissue harvest. Testis, prostate, ovary, kidney, and gonadal fat pads were fixed in Bouin’s solution (Sigma) followed by 70% ethanol, then processed for paraffin embedding and hematoxylin, and eosin (H & E) staining by standard procedures for histopathological examination. Paraffin five micron sections were processed, stained, and provided by Nationwide Histology, Spokane WA, USA.

### Histopathology examination and disease classification

The oversight of the pathology analysis involved the co-author, Dr. Eric Nilsson, DVM/PhD, with over 20 years of pathology analysis in rats^45,46^. The Washington Animal Disease Diagnostic Laboratory (WADDL) at the Washington State University College of Veterinary Medicine has board certified veterinary pathologists and assisted in initially establishing the criteria for the pathology analyses and identifying parameters to assess^37^. WADDL performed full necropsies as required on animals that died prior to the time of scheduled sacrifice at one year, and performed tumor classifications in the current study.

Upon dissection a brief examination of abdominal and thoracic organs was performed to look for obvious abnormalities. The current study found no significant gross pathology of heart, lung, liver, gastro-intestinal track, or spleen. The tissues evaluated histologically were selected from previous literature showing them to have pathology in transgenerational models^36–45^, with an emphasis on reproductive organs. Histopathology readers were trained to recognize the specific abnormalities evaluated for this study in rat testis, ventral prostate, ovary and kidney (see below). Three different pathology readers were used for each tissue and were blinded to the treatment groups. A set of quality control (QC) slides was generated for each tissue and was read by each reader prior to evaluating any set of experimental slides. These QC slide results are monitored for reader accuracy and concordance. WADDL was consulted when any questions developed. Previous studies by the laboratory help confirm and validate the pathology analysis^36–45^.

As previously described^29^, testis histopathology criteria included the presence of vacuoles in the seminiferous tubules, azoospermic atretic seminiferous tubules, and ‘other’ abnormalities including sloughed spermatogenic cells in center of the tubule and a lack of a tubule lumen (Supplemental Figure S2). As previously described^46,79^, prostate histopathology criteria included the presence of vacuoles in the glandular epithelium, atrophic glandular epithelium and hyperplasia of prostatic gland epithelium (Supplemental Figure S2). Kidney histopathology criteria included reduced size of glomerulus, thickened Bowman’s capsule, and the presence of proteinaceous fluid-filled cysts >50 μm in diameter (Supplemental Figure S2). Ovary sections were assessed for the two pathologies of primordial follicle loss and ovarian cysts, as previously described^48^ (Supplemental Figure S2). Ovarian cysts have little or no granulosa cell layer, a smooth border, and are 50–250 μm (small cysts) or >250 μm (large cysts) in diameter. A cut-off was established to declare a tissue ‘diseased’ based on the mean number of histopathological abnormalities plus two standard deviations from the mean of control group tissues, as assessed by each of the three individual observers blinded to the treatment groups. This number (i.e. greater than two standard deviations) was used to classify rats into those with and without testis, ovary, prostate, or kidney disease in each lineage. A rat tissue section was finally declared ‘diseased’ only when at least two of the three observers marked the same tissue section ‘diseased’.

Obesity was assessed with an increase in adipocyte size (area), body mass index (BMI) and abdominal adiposity, as previously described^40,41,80–82^. BMI was calculated with weight (g)/length (cm)^2^ with the length of the animal measured from the nose to the base of the tail. Gonadal fat pad slides were imaged using a Nikon Eclipse E800 microscope (10×) with an AVT Prosilica GE1050C Color GigE camera. Five field of view image captures were taken per slide in varying parts of the fat pad. Adipocyte size was measured converting pixels into microns using Adiposoft^83^. Measurements of the 20 largest cells from each image for a total of 100 were averaged as hypertrophic cells are the most metabolically relevant and susceptible to cell death^84^. Obesity and lean phenotypes were determined utilizing the mean of the control population males and females, and a cut off of 1.5 standard deviations above and below the mean (Supplemental Figure S2).

#### Behavior analysis

As previously described^45^, behavior analysis was performed to evaluate general anxiety^85^ with both an elevated plus maze and Light and Dark, box as previously described^86,87^. F3 generation male and female rats from control and glyphosate lineages were used for the behavioral studies at 11 months of age. The elevated plus-maze consisted of a “plus”-shaped platform made of black opaque Plexiglas, with each platform 10 cm in width and 50 cm in length, creating a 10 × 10 cm neutral zone in the center. Two of the arms were enclosed with black Plexiglas walls 40 cm high, with no ceiling. For this task, rats were placed individually into the center (neutral) zone of the maze, facing an open arm. Rats were allowed to explore for a 5 min period, and the number of open and closed arm entries and time spent on the open and closed arms were recorded. The Light and Dark box consists of a small dark compartment that made up one third of the apparatus with the other two thirds being an illuminated compartment. The rats were placed individually in the light zone, facing the dark zone, as previously recommended^88,89^. Similarly, the number light and dark compartment entries and time spent on the light and dark compartments were recorded.

#### Statistical analyses for pathology

As previously described^76^, for results that yielded continuous data (age at puberty, weight at euthanization, sex ratio, litter size, fertility rate, parturition abnormality behavioral parameters), treatment groups were analyzed using Student’s t-test. For results expressed as the proportion of affected animals that exceeded a pre-determined threshold (testis, prostate, kidney or ovary disease frequency, tumor frequency, lean/obese frequency), groups were analyzed using Fisher’s exact test.

### Sperm Epigenetic Analysis

#### Epididymal sperm collection and DNA isolation

The protocol is described in detail in reference76. Briefly, the epididymis was dissected free of fat and connective tissue, then, after cutting open the cauda, placed into 6 ml of phosphate buffer saline (PBS) for 20 minutes at room temperature. Further incubation at 4 °C will immobilize the sperm. The tissue was then minced, the released sperm pelleted at 4 °C 3,000 × *g* for 10 min, then resuspended in NIM buffer and stored at −80 °C for further processing.

An appropriate amount of rat sperm suspension was used for DNA extraction. Previous studies have shown mammalian sperm heads are resistant to sonication unlike somatic cells^90,91^. Somatic cells and debris were therefore removed by brief sonication (Fisher Sonic Dismembrator, model 300, power 25), then centrifugation and washing 1–2 times in 1xPBS. The resulting pellet was resuspended in 820 μL DNA extraction buffer and 80 μl 0.1 M DTT added, then incubated at 65 °C for 15 minutes. 80 μl proteinase K (20 mg/ml) was added and the sample was incubated at 55 °C for 2–4 hours under constant rotation. Protein was removed by addition of protein precipitation solution (300 μl, Promega A795A), incubation for 15 min on ice, then centrifugation at 13,500 rpm for 30 minutes at 4 °C. One ml of the supernatant was precipitated with 2 μl of glycoblue (Invitrogen, AM9516) and 1 ml of cold 100% isopropanol. After incubation, the sample was spun at 13,500 × g for 30 min at 4 °C, then washed with 70% cold ethanol. The pellet was air-dried for about 5 minutes then resuspended in 100 μl of nuclease free water. For all generations, equal amounts of DNA from each individual’s sample was used to produce 6 different DNA pools per lineage and the pooled DNA used for methylated DNA immunoprecipitation (MeDIP).

#### Methylated DNA Immunoprecipitation (MeDIP)

The protocol is described in detail in reference^76^. Genomic DNA was sonicated and run on 1.5% agarose gel for fragment size verification. The sonicated DNA was then diluted with TE buffer to 400 μl, then heat-denatured for 10 min at 95 °C, and immediately cooled on ice for 10 min to create single-stranded DNA fragments. Then 100 μl of 5X IP buffer and 5 μg of antibody (monoclonal mouse anti 5-methyl cytidine; Diagenode #C15200006) were added, and the mixture was incubated overnight on a rotator at 4 °C. The following day magnetic beads (Dynabeads M-280 Sheep anti-Mouse IgG; Life Technologies 11201D) were pre-washed per manufacturer’s instructions, and 50 μl of beads were added to the 500 μl of DNA-antibody mixture from the overnight incubation, then incubated for 2 h on a rotator at 4 °C. After this incubation, the samples were washed three times with 1X IP buffer using a magnetic rack. The washed samples were then resuspended in 250 μl digestion buffer (5 mM Tris PH 8, 10.mM EDTA, 0.5% SDS) with 3.5 μl Proteinase K (20 mg/ml), and incubated for 2–3 hours on a rotator at 55°. DNA clean-up was performed using a Phenol-Chloroform-Isoamyalcohol extraction, and the supernatant precipitated with 2 μl of glycoblue (20 mg/ml), 20 μl of 5 M NaCl and 500 μl ethanol in −20 °C freezer for one to several hours. The DNA precipitate was pelleted, washed with 70% ethanol, then dried and resuspended in 20 μl H_2_O or TE. DNA concentration was measured in Qubit (Life Technologies) with the ssDNA kit (Molecular Probes Q10212).

#### MeDIP-Seq Analysis

MeDIP DNA was used to create libraries for next generation sequencing (NGS) using the NEBNext® Ultra™ RNA Library Prep Kit for Illumina® (San Diego, CA) starting at step 1.4 of the manufacturer’s protocol to generate double stranded DNA from the single-stranded DNA resulting from MeDIP. After this step, the manufacturer’s protocol was followed indexing each sample individually with NEBNext Multiplex Oligos for Illumina. The WSU Spokane Genomics Core sequenced the samples on the Illumina HiSeq 2500 at PE50, with a read size of approximately 50 bp and approximately 20 million reads per pool. Twelve libraries were run in one lane.

#### Statistics and Bioinformatics

The DMR identification and annotation methods follow those presented in previous published papers^45,54^. Data quality was assessed using the FastQC program (https://www.bioinformatics.babraham.ac.uk/projects/fastqc/), and reads were cleaned and filtered to remove adapters and low quality bases using Trimmomatic^92^. The basic read quality was verified using summaries produced by the FastQC program. The new data was cleaned and filtered to remove adapters and low quality bases using Trimmomatic^92^. The reads for each MeDIP sample were mapped to the Rnor 6.0 rat genome using Bowtie2^93^ with default parameter options. The mapped read files were then converted to sorted BAM files using SAMtools^83^. The MEDIPS R package^84^ was used to calculate differential coverage between control and exposure sample groups. The edgeR p-value^94^ was used to determine the relative difference between the two groups for each genomic window. Windows with an edgeR p-value less than an arbitrarily selected threshold were considered DMRs. The DMR edges were extended until no genomic window with an edgeR p-value less than 0.1 remained within 1000 bp of the DMR. The false discovery rate (FDR) p-value was <0.05 for all DMRs identified at an edgeR p-value < 1e-06.

DMRs were annotated using the biomaRt R package^95^ to access the Ensembl database^96^. The genes that associated with DMR were then input into the KEGG pathway search^97,98^ to identify associated pathways. The DMR associated genes were then automatically sorted into functional groups using information provided by the DAVID^99^ and Panther^100^ databases incorporated into an internal curated database (www.skinner.wsu.edu under genomic data). All molecular data has been deposited into the public database at NCBI (GEO # GSE118557) and R code computational tools available at GitHub (https://github.com/skinnerlab/MeDIP-seq) and www.skinner.wsu.edu.

## Supplementary information

Supplemental Material

## Data Availability

All molecular data has been deposited into the public database at NCBI (GEO # GSE118557) and R code computational tools available at GitHub (https://github.com/skinnerlab/MeDIP-seq) and www.skinner.wsu.edu.
