# Supplementary material for: Assessment of Glyphosate Induced Epigenetic Transgenerational Inheritance of Pathologies and Sperm Epimutations: Generational Toxicology
Source: Sci Rep. 2019 Apr 23;9:6372. doi: 10.1038/s41598-019-42860-0 (PMC6476885; doi:10.1038/s41598-019-42860-0)
Supplement: Supplementary file 1 — Supplemental Material [file 41598_2019_42860_MOESM1_ESM.pdf]

Glyphosate Induced Epigenetic Transgenerational Inheritance of Pathologies and Sperm Epimutations: Generational Toxicology

Deepika Kubsad, Eric E. Nilsson, Stephanie E. King, Ingrid Sadler-Riggleman, Daniel Beck and *Michael K. Skinner

Center for Reproductive Biology
School of Biological Sciences
Washington State University
Pullman, WA, 99164-4236, USA

Supplemental Material

Supplemental Figure and Table Legends

Figure S1. Glyphosate usage. **(a)** Estimated USA agricultural use for glyphosate in 2015. **(b)** Glyphosate use by year and crop. From U.S. Geological Survey Pesticide Use Maps, accessed February 12, 2018. (https://water.usgs.gov/nawqa/pnsp/usage/maps/show_map.php?year=2011&map=GLYPHOSATE&hilo=L&disp=Glyphosate)

Figure S2. F3 generation glyphosate and control histopathology. **(a)** Testis histological abnormalities. A. Atrophic seminiferous tubule showing reduced Sertoli and germ cells (black arrow). Blue arrowhead indicates a round, smooth-edged, basally located vacuole. **(b)** Control testis showing normal seminiferous tubules. Scale bars = 100 μm. **(c)** Prostate histological abnormalities. Prostate glands showing thinning atrophic glandular epithelium (black arrows). **(d)** Normal prostate glands with normal thickness epithelium. **(e)** Prostate gland with epithelial hyperplasia (blue arrowhead). **(f)** Small vacuole in prostate epithelial layer of gland (red arrow). Scale bars = 100 μm. Kidney histological abnormalities. **(g)** Kidney histological abnormalities. Severely affected kidney showing a glomerulus (blue arrowhead) with a reduced area of the glomerular tuft and a thickened Bowman's capsule. Black arrows show examples of cysts, likely of renal tubular origin. **(h)** Kidney showing histologically normal glomeruli and renal tubules. Scale bars = 100 μm. **(i)** Ovary histological features counted. Large ovarian cysts (blue arrows), distinguished from follicles by the absence of a distinct granulosa cell layer. **(j)** Primordial (red arrows) and primary (black arrow) ovarian follicles are indicated. Other pre-antral follicles lower in this image were not counted as their oocytes did not have clearly visible nuclei. Scale bars =

100 μm. **(k)** Adipocyte size histological abnormalities. Adipocyte area with enlarge size (6129 μm^2) from F3 generation glyphosate lineage gonadal fat pad. Scale bars = 100 μm. **(l)** Adipocyte area with normal size (3103 μm^2) from F3 generation control lineage gonadal fat pad.

Figure S3. Additional pathology data. **(a)** Fertility rates, **(b)** litter size, and **(c)** F3 generation gestational length (days). The mean ± SEM presented.

Figure S4. Behavior analysis. Bars on left of panels / graph are female and right of panels / graph are male for control and glyphosate lineage F3 generation. LDB = light-dark box. EPM = elevated plus maze. The mean ± SEM presented and (*) indicates $p<0.05$.

Figure S5. DMR genomic features. **(a, c, e)** The number of DMRs at different CpG densities for all DMRs at a p-value threshold of $p<1x10^{-6}$. **(b, d, f)** The DMR lengths for all DMRs at a p-value threshold of $p<1x10^{-6}$. **(a, b)** F1 generation. **(c, d)** F2 generation. **(e, f)** F3 generation.

Figure S6. F1 and F2 generation DMR principle component analysis (PCA). The F1 and F2 generation DMRs and not whole genome were used for the PCA analysis. All DMRs are defined using an edgeR p-value threshold of 1e-06. **(a)** PCA F1 generation control and glyphosate analysis with legend insert. **(b)** PCA F2 generation control and glyphosate DMR analysis with legend insert. The control (PBS), control (DMSO) and glyphosate generation lineage (GF1 or GF2) indicated.

Figure S7. Founder effect. **(a)** Normalized Obesity Frequency of generation offspring to determine lineage effects. Fifteen wild type males were assessed for the frequency of obesity phenotype which had offspring in five different colonies. The number of males or females in each litter (N_L) was divided by the number of males and females in the colony (N_C) in order to control for litter size. The frequency of obesity in the litter (f_l) was multiplied by (N_L/N_C). The product was divided the frequency of obesity in the colony (f_c) to create a normalized frequency of obesity in the offspring (f_n). The average of all (f_n) litters the wild type sired was subtracted by (f_c) to assess the lineage based attributed phenotype. Wildtype 41 (W41) was identified as having a larger attributed phenotype and was the ancestral sire of the two unusual litters previously mentioned. The F1 generation litter he sired had five out of five females and one out of 4 males (67% frequency) that were determined to be obese. As the lineage was determined to be an outlier, it was removed from the study. **(b)** During the assessment of obesity in the F3 generation glyphosate and glyphosate control lineage rats, two unusual litters had high levels of obesity (88% frequency) in comparison to the rest of the colony (20% frequency). To determine if this effect was a founder's effect lineage phenomenon, the litters were traced back to a common ancestor. The F1 and F2 generation glyphosate and glyphosate control lineage rats were assessed for obesity and a formula was developed to normalize expected obesity by treatment group based on colony frequency.

Table S1. F1 generation individual animal pathology. **(a)** Glyphosate Male. **(b)** Control Male. **(c)** Glyphosate Female. **(d)** Control Female. (+) Indicates pathology present and (-) indicates no pathology and blank box not examined.

Table S2. F2 generation individual animal pathology. **(a)** Glyphosate Male. **(b)** Control Male. **(c)** Glyphosate Female. **(d)** Control Female. (+) Indicates pathology present and (-) indicates no pathology and blank box not examined.

Table S3. F3 generation individual animal pathology. **(a)** Glyphosate Male. **(b)** Control Male. **(c)** Glyphosate Female. **(d)** Control Female. (+) Indicates pathology present and (-) indicates no pathology and blank box not examined.

Table S4. F1 generation DMR list.

Table S5. F2 generation DMR list.

Table S6. F3 generation DMR list.

Figure S1

a Estimated USA agricultural use for glyphosate, 2015

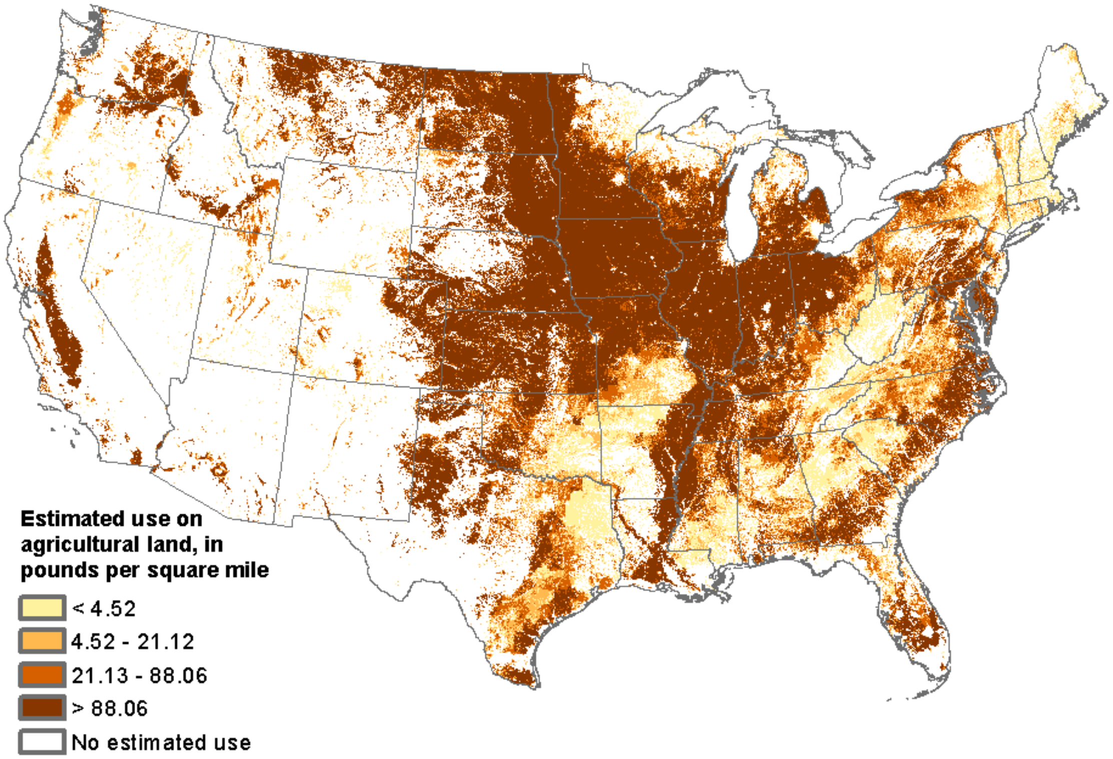

b Glyphosate use by year and crop

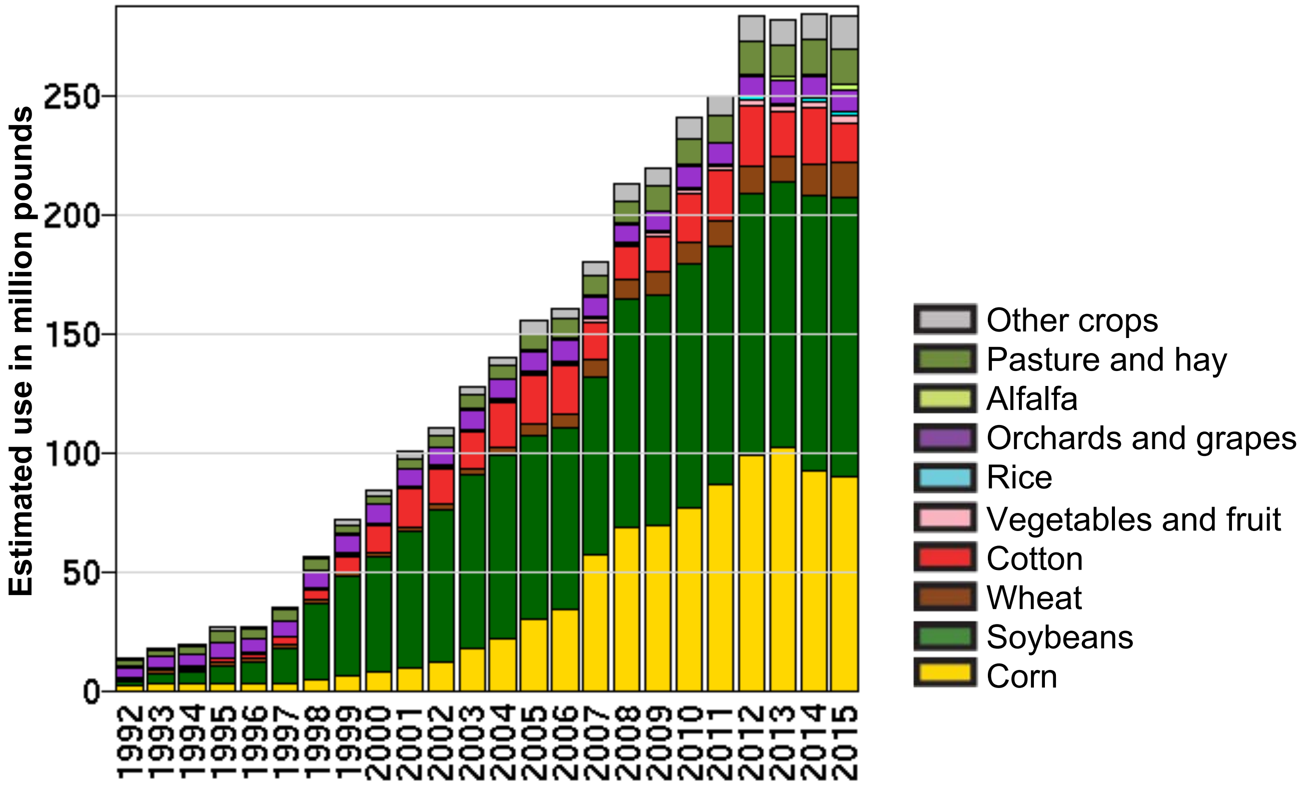

Figure S2

Testis Pathology (F3 Generation)

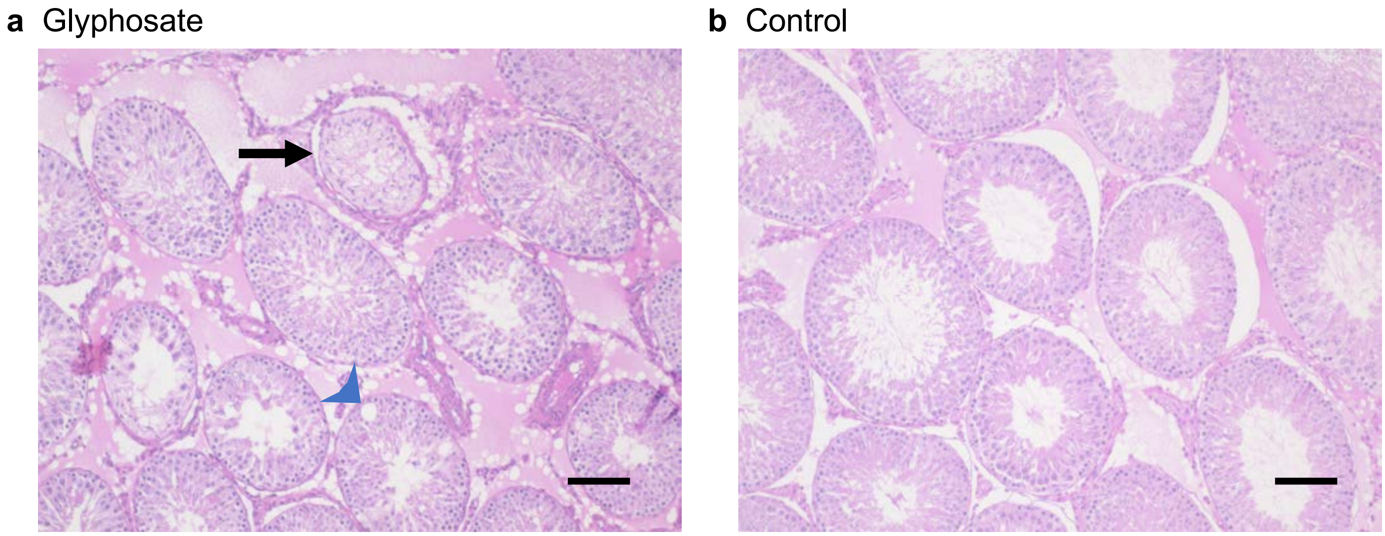

Prostate Pathology (F3 Generation)

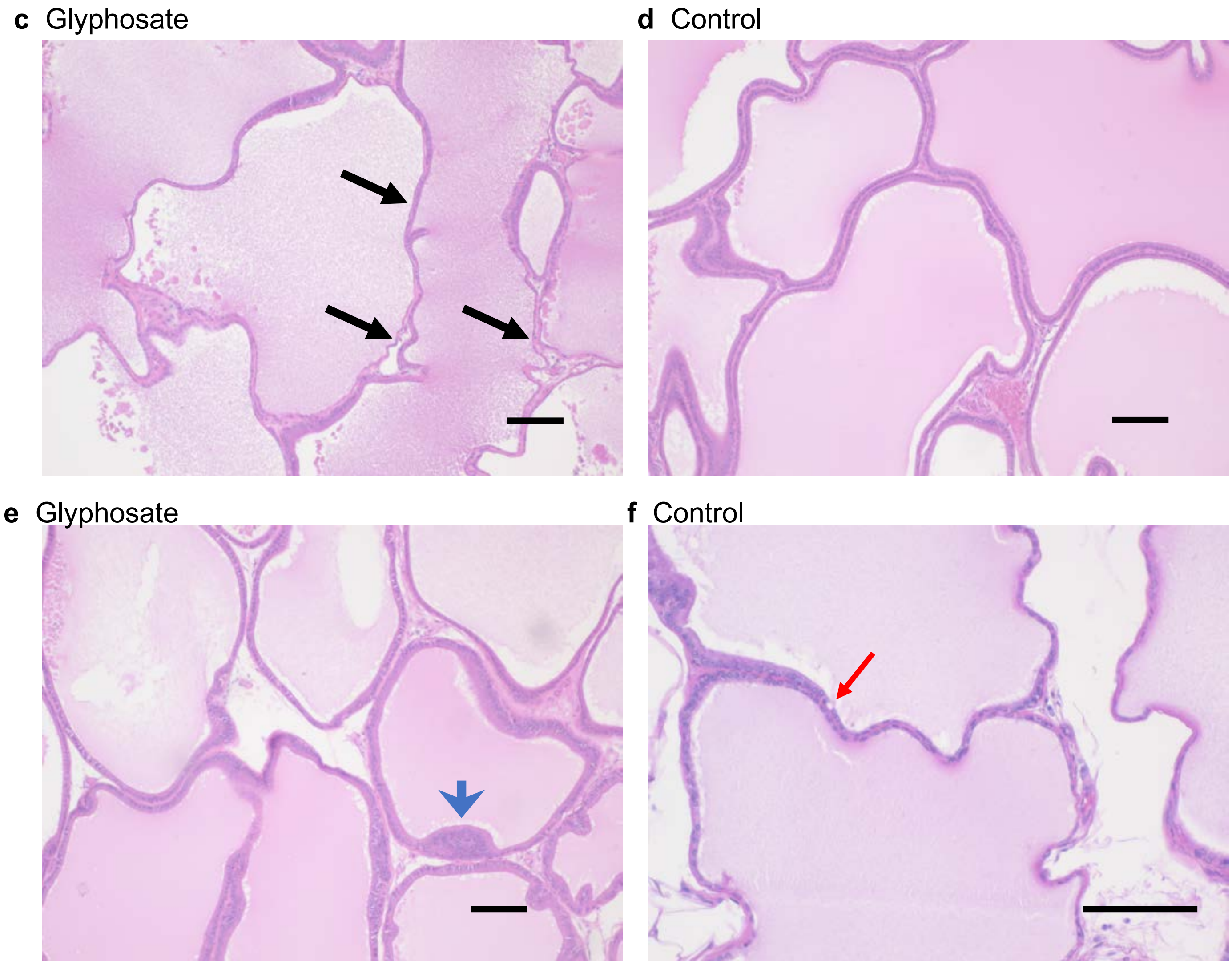

Figure S2 (continued)

Kidney Pathology (F3 Generation)

g Glyphosate

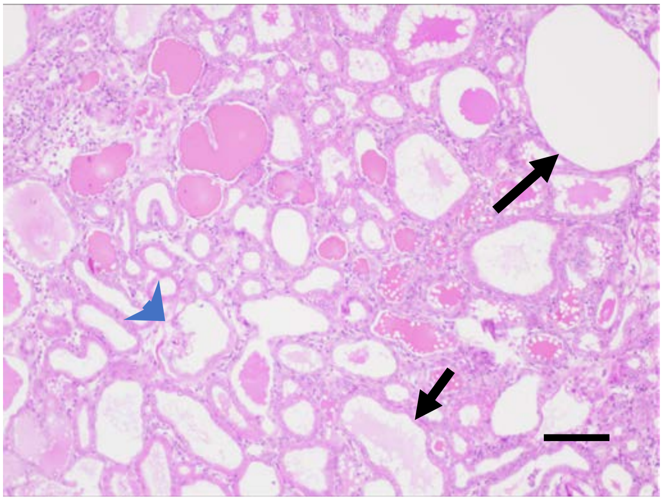

h Control

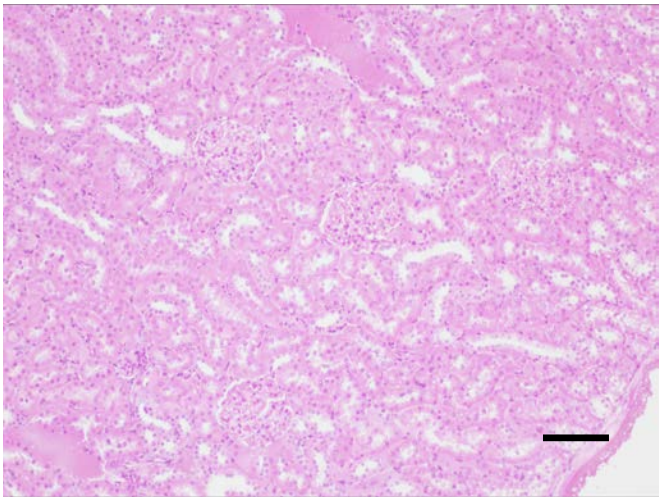

Ovary Pathology (F3 Generation)

i Glyphosate

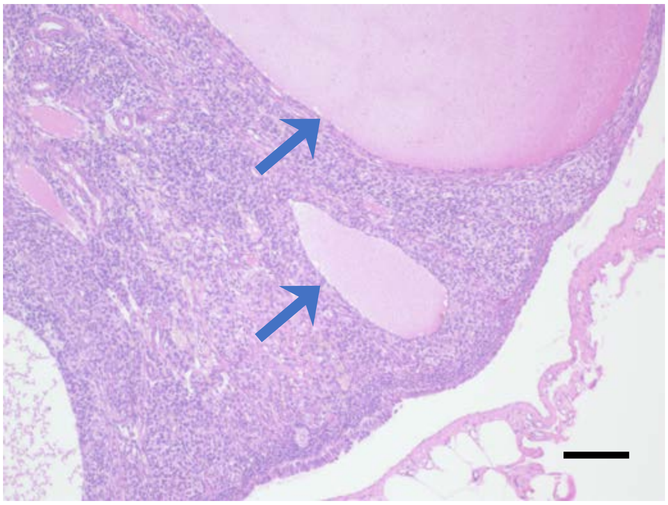

j Control

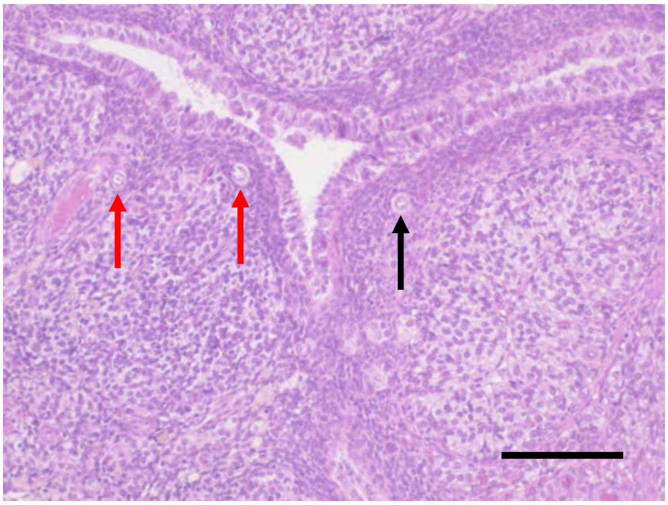

Adipocyte Pathology (F3 Generation)

k Glyphosate

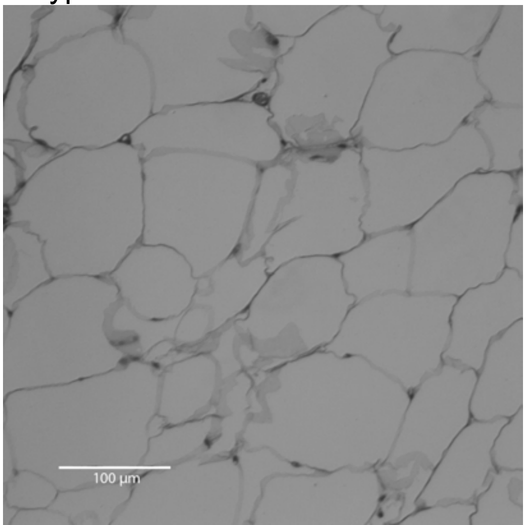

l Control

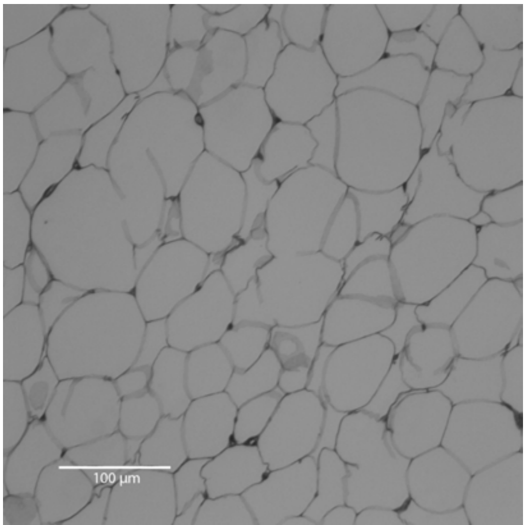

Figure S3

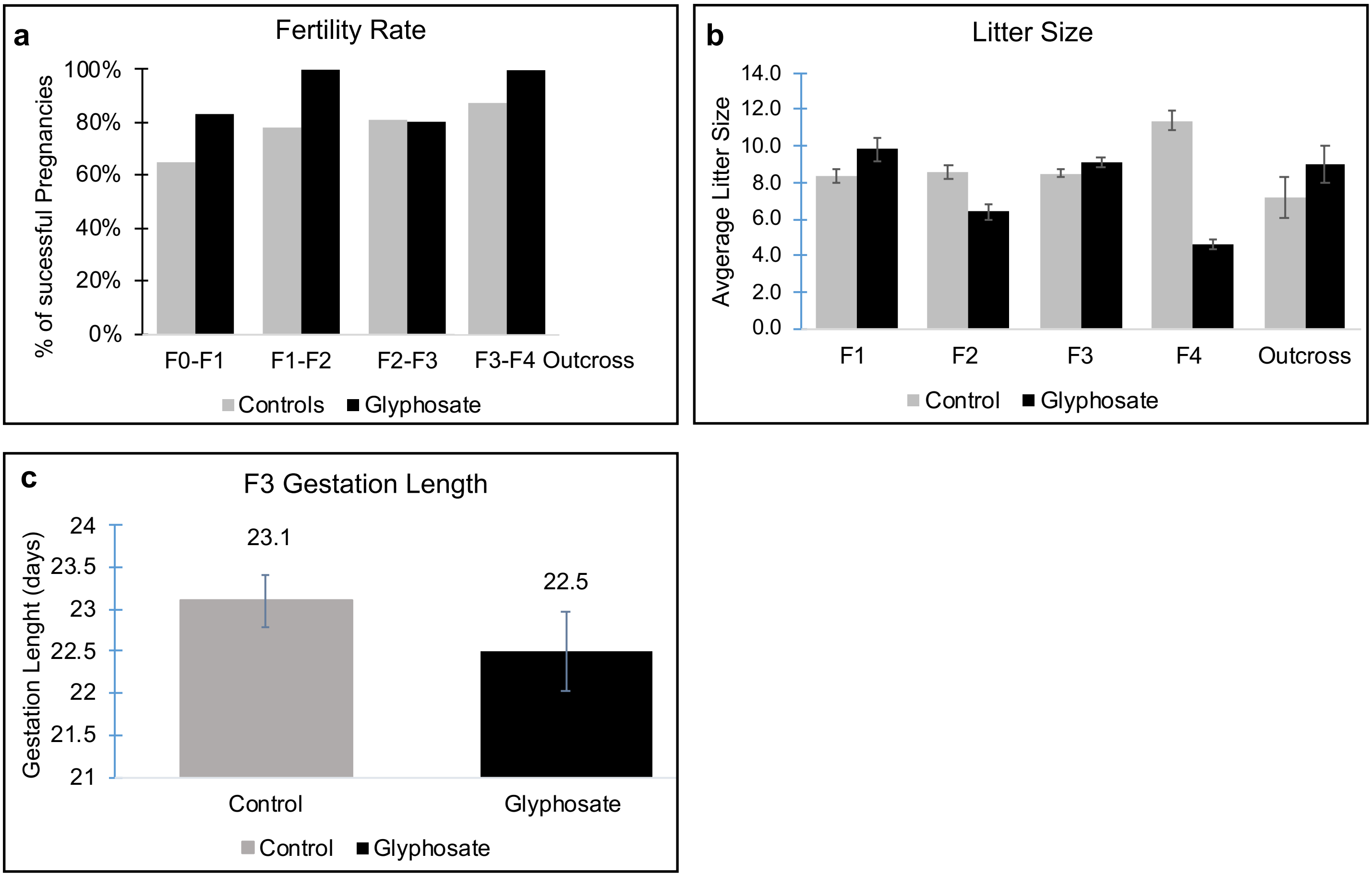
a
Fertility Rate
% of sucessful Pregnancies
100%
80%
60%
40%
20%
0%
F0-F1
F1-F2
F2-F3
F3-F4 Outcross
Controls
Glyphosate
b
Litter Size
Avgerage Litter Size
14.0
12.0
10.0
8.0
6.0
4.0
2.0
0.0
F1
F2
F3
F4
Outcross
Control
Glyphosate
c
F3 Gestation Length
Gestation Lenght (days)
24
23.5
23
22.5
22
21.5
21
23.1
22.5
Control
Glyphosate
Control
Glyphosate

Figure S4

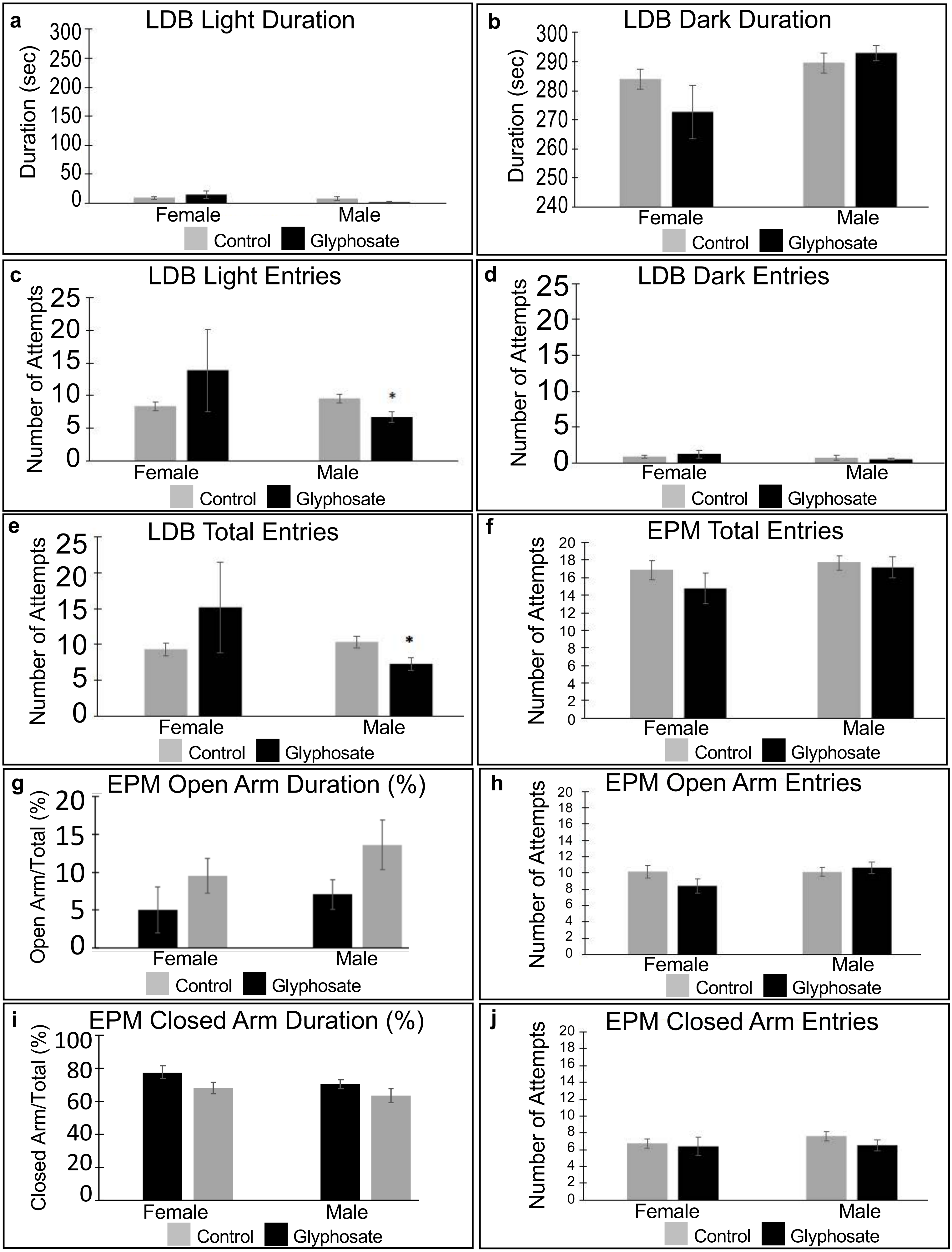

a
LDB Light Duration
Duration (sec)
300
250
200
150
100
50
0
Female
Male
Control
Glyphosate
b
LDB Dark Duration
Duration (sec)
300
290
280
270
260
250
240
Female
Male
Control
Glyphosate
c
LDB Light Entries
Number of Attempts
25
20
15
10
5
0
*
Female
Male
Control
Glyphosate
d
LDB Dark Entries
Number of Attempts
25
20
15
10
5
0
Female
Male
Control
Glyphosate
e
LDB Total Entries
Number of Attempts
25
20
15
10
5
0
*
Female
Male
Control
Glyphosate
f
EPM Total Entries
Number of Attempts
20
18
16
14
12
10
8
6
4
2
0
Female
Male
Control
Glyphosate
g
EPM Open Arm Duration (%)
Open Arm/Total (%)
20
15
10
5
0
Female
Male
Control
Glyphosate
h
EPM Open Arm Entries
Number of Attempts
20
18
16
14
12
10
8
6
4
2
0
Female
Male
Control
Glyphosate
i
EPM Closed Arm Duration (%)
Closed Arm/Total (%)
100
80
60
40
20
0
Female
Male
Control
Glyphosate
j
EPM Closed Arm Entries
Number of Attempts
20
18
16
14
12
10
8
6
4
2
0
Female
Male
Control
Glyphosate

Figure S5 DMR density and length

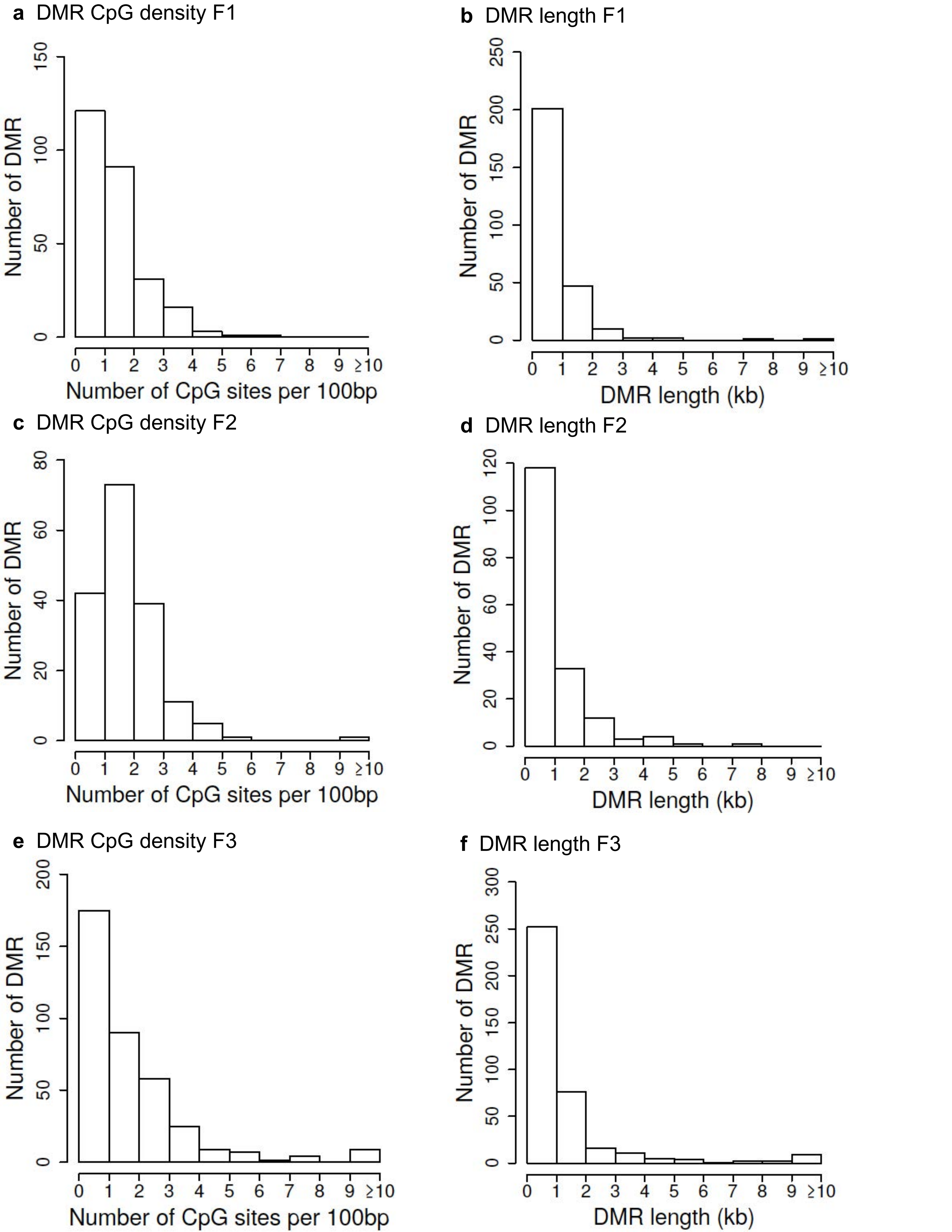

Figure S6

a PCA F1 DMR

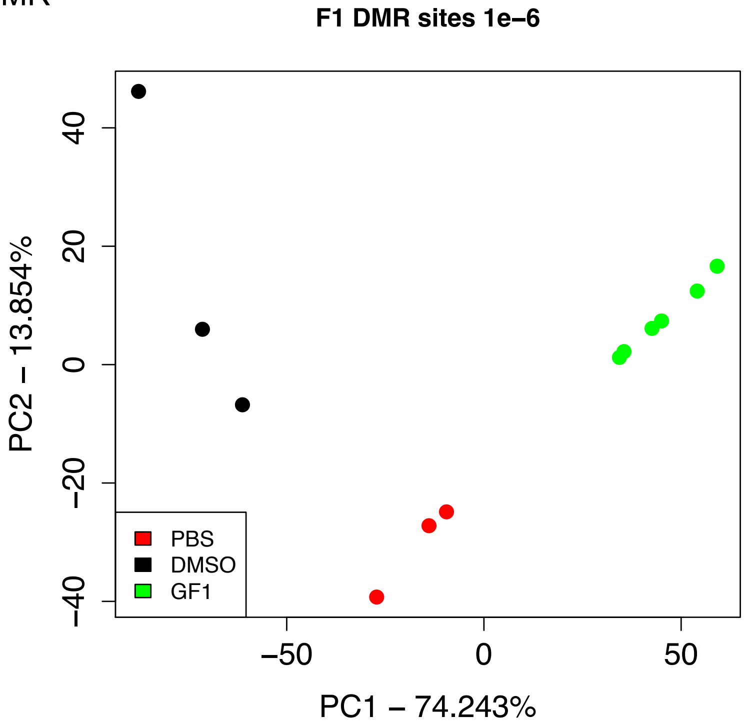

b PCA F2 DMR

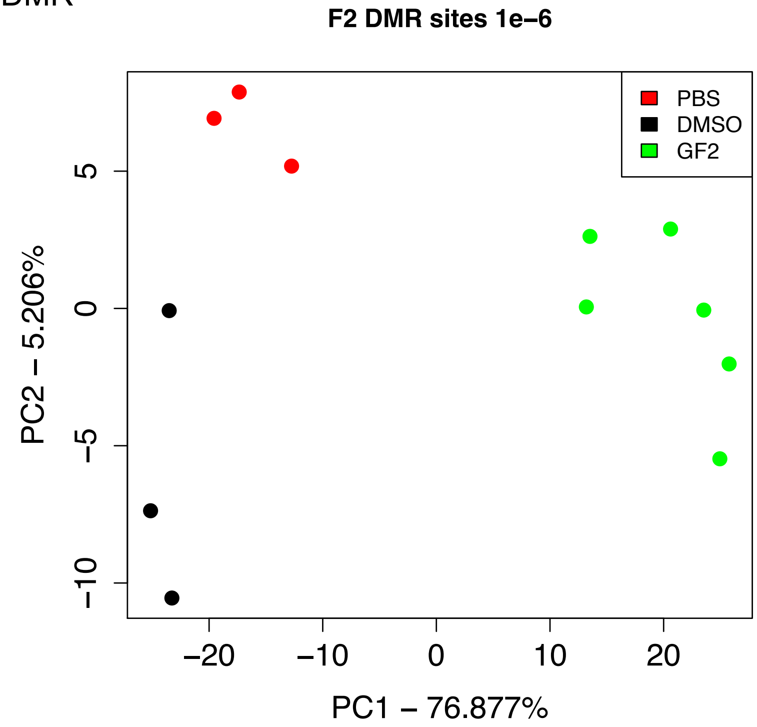

Figure S7

a Normalization obesity frequency of generation offspring to determine lineage effects

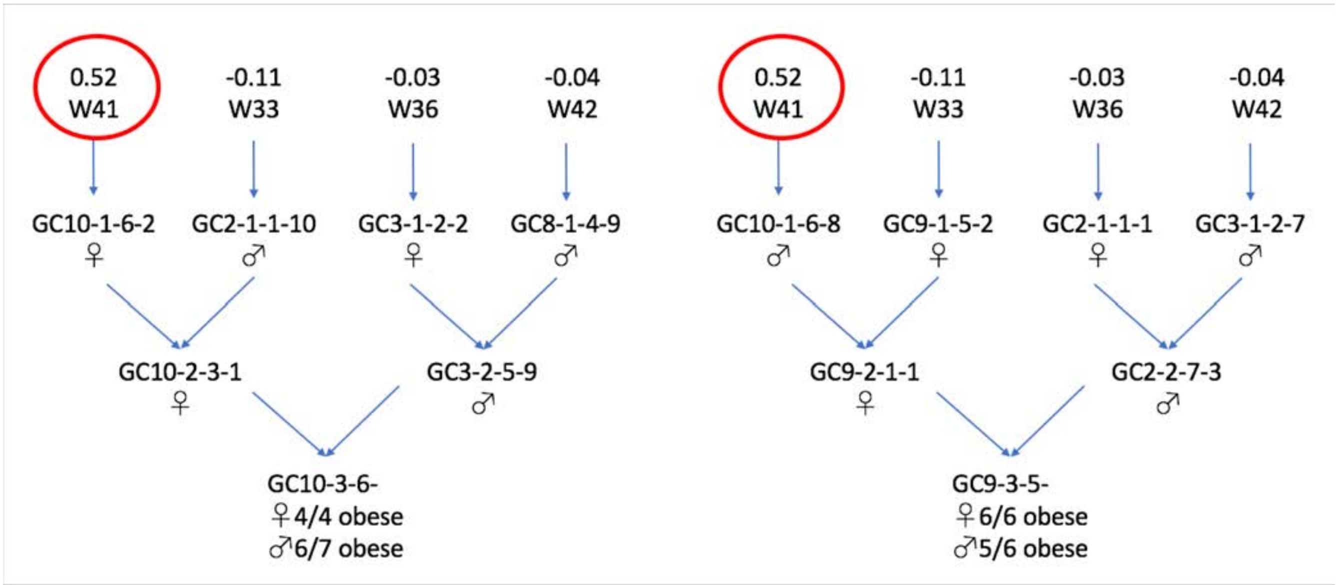

b Founder effect

f_n = normalized frequency of obesity in offspring

N_L = ♂ or ♀ in litter

N_C = # of ♂ or ♀ in colony (controlling for litter size)

f_l = frequency of obesity in litter

f_c = frequency of obesity in colony

$$f_n = \frac{\left({}^{N_L}/_{N_C}\right) f_l}{f_c}$$

Lineage based attributed risk = $\bar{x}(f_n)$ - $\bar{x}(f_c)$

Supplemental Table S1

F1 Individual Animal Pathology

(A) F1 Generation Glyphosate Males

Rat ID	Puberty	Testis	Prostate	Kidney	Tumor	Lean	Obese	Total Disease
15GG1-1-1-6	-	-	-	-	-	+	-	1
15GG1-1-1-7	-	-	+	-	-	+	-	2
15GG1-1-1-8	-	+	+	-	-	-	-	2
15GG1-1-1-9	-	-	-	-	-	-	-	
15GG1-1-1-11	-	-	-	-	-	+	-	1
15GG1-1-1-12		+	-	-	-	-	+	2
15GG1-1-1-13	-	-	+	+	-	+	-	3
15GG1-1-1-14		-	-	+	-	+	-	2
15GG2-1-2-6	-	-	-	-	-	-	+	1
15GG2-1-2-7	-	-	-	-	-	-	-	
15GG3-1-3-1		-	-	+	-	-	+	2
15GG3-1-3-2		-	-	-	-	-	-	
15GG3-1-3-3		-	-	-	-	-	-	
15GG3-1-3-4		-	-	-	-	-	-	
15GG3-1-3-5		-	-	-	-	-	+	1
15GG3-1-3-6		-	-	+	-	-	-	1
15GG4-1-4-6		+	-	+	-	-	+	3
15GG4-1-4-7	-	+	-	+	-	-	+	3
15GG5-1-5-9		-	-	+	-	+	-	2
15GG5-1-5-10		-	-	-	-	+	-	1
15GG5-1-5-11		-	-	-	-	+	-	1
15GG5-1-5-12		-	-	-	-	+	-	1
15GG5-1-5-13		-	-	-	-	+	-	1
15GG5-1-5-14		-	-	-	-	+	-	1
15GG5-1-5-15		-	-	-	-	-	-	
15GG6-1-6-5		-	-	+	-	-	-	1
15GG6-1-6-6	+	-	+	+	-	-	-	3
15GG6-1-6-7	+	-	-	-	-	+	-	2
15GG6-1-6-8	+	-	-	-	-	-	-	1
15GG6-1-6-9	+	-	-	-	-	+	-	2
15GG6-1-6-10	+	-	+	+	-	-	-	3
Affected	5	4	5	10	0	13	6	
Population	14	31	31	31	31	31	31	

(B) F1 Generation Control Males

	Puberty	Testis	Prostate	Kidney	Tumor	Lean	Obese	Total Disease
Rat ID								
15GC2-1-1-9		+	-	-	-	+	-	2
15GC2-1-1-10		-	-	-	-	-	-	
15GC2-1-1-11		-	-	-	-	-	-	
15GC2-1-1-12		-	-	-	-	-	-	
15GC2-1-1-13		+	-	-	-	-	-	1
15GC3-1-2-7	-	-	-	-	-	-	+	1
15GC3-1-2-8	-	-	-	-	-	+	-	1
15GC3-1-2-9	-	-	-	-	-	-	+	1
15GC3-1-2-10		-	-	-	-	+	-	1
15GC3-1-2-11	-	-	-	-	-	-	-	
15GC3-1-2-12	-	-	-	-	-	+	-	1
15GC3-1-2-14	-	-	-	-	-	+	-	1
15GC3-1-2-15	-	-	-	-	+			1
15GC8-1-4-8		-	-	-	-	+	-	1
15GC8-1-4-9		+	-	-	-	+	-	2
15GC8-1-4-10		+	+	-	-	+	-	3
15GC8-1-4-11		-	-	-	-	-	-	
15GC8-1-4-12		-	-	-	-	-	-	
15GC8-1-4-13		+	-	-	-	+	-	2
15GC8-1-4-14		+	-	-	-	+	-	2
15GC8-1-4-15		+	+	-	-	+	-	3
15GC8-1-4-16		-	-	-	-	+	-	1
15GC9-1-5-5		-	-	+	-	-	-	
15GC11-1-7-5	-	-		-	-	-	-	
AC2-1-2-1	+	-	-	+	-	-	-	2
AC2-1-2-2	-	-	-	-	-	-	-	
AC2-1-2-3	-	-	-	+	-	-	-	1
AC2-1-2-4	-	-		-	-	-	-	
AC2-1-2-5	-	-	-	-	-	-	-	
AC3-1-3-1	-				-	-	+	1
AC4-1-4-1	-				-	-	+	1
AC4-1-4-2	-				-	-	-	
AC4-1-4-3	-				-	-	-	
AC4-1-4-4	-				-	-	-	
AC6-1-6-1	-	-	-	-	-	-	-	
AC6-1-6-2	-		+	+	-	-	-	2
AC6-1-6-3	-	-	-	-	-	-	+	1
AC6-1-6-4		+	+	+	-	+	-	4
AC9-1-4-7	-	-	-	-	-	-	-	
AC9-1-4-8	-	-		-	-	-	-	
14C10-1-10-7	-	-	-	-	-	-	+	1
14C10-1-10-8	-	-	-	-	-	-	+	1
14C10-1-10-9		+	-	-	-	-	+	2
14C10-1-10-10		-	-	-	-	-	-	
14C12-1-3-5	-				-	-	+	1
14C16-1-6-6	-				-	-	-	
14C17-1-17-4	-				-	-	-	
14C17-1-17-5	-				-	-	-	
Affected	1	9	4	5	1	13	9	
Population	27	38	36	39	48	47	47	

(C) F1 Generation Glyphosate Females

	Puberty	Ovary	Kidney	Tumor	Lean	Obese	Total Disease
Rat ID							
15GG1-1-1-1		-	-	-	+	-	1
15GG1-1-1-2		+	-	-	-	-	
15GG1-1-1-3		-	-	-	-	-	
15GG1-1-1-4	-	+	-	-	+	-	2
15GG1-1-1-5	-	-	-	-	-	-	
15GG1-1-1-10		+	-	-	-	-	1
15GG2-1-2-1		+	-	-	-	+	2
15GG2-1-2-2	-	-	-	-	-	+	1
15GG2-1-2-3	-	-	-	-	-	+	1
15GG2-1-2-4		+	-	-	-	+	2
15GG2-1-2-5	-	-	-	-	-	+	1
15GG4-1-4-1	-	-	-	-	-	-	
15GG4-1-4-2	-	+	-	-	-	-	1
15GG4-1-4-3	-	-	+	-	-	-	1
15GG4-1-4-4	-	-	-	-	-	+	1
15GG4-1-4-5	-	-	-	-	-	+	1
15GG5-1-5-1		-	-	-	+	-	1
15GG5-1-5-2		-	-	-	+	-	1
15GG5-1-5-3		-	-	-	-	-	
15GG5-1-5-4		-	-	-	+	-	1
15GG5-1-5-5		-	-	-	+	-	1
15GG5-1-5-6		-	-	-	+	-	1
15GG5-1-5-7		-	-	-	+	-	1
15GG5-1-5-8		-	-	-	-	-	
15GG6-1-6-1		-	-	-			
15GG6-1-6-2	-	-	-	-	-	-	
15GG6-1-6-3		-	-	-	-	+	1
15GG6-1-6-4		-	-	-	-	-	
Affected	0	**6**	**1**	**0**	**8**	8	
Population	11	**28**	**28**	**28**	**27**	27	

(D) F1 Generation Control Females

Rat ID	Puberty	Ovary	Kidney	Tumor	Lean	Obese	Total Disease
15GC2-1-1-1		-	-	-	-	-	
15GC2-1-1-2		-	-	-	-	-	
15GC2-1-1-3		-	-	-	-	-	
15GC2-1-1-4		-	-	-	-	-	
15GC2-1-1-5		+	-	-	-	-	1
15GC3-1-2-1	-			-			
15GC3-1-2-2	-	-	-	-	-	-	
15GC3-1-2-3	-	-	+	-	-	-	1
15GC3-1-2-4	-	-	-	-	-	-	
15GC3-1-2-5		-	-	-	+	-	1
15GC6-1-3-1		-	-	-	-	-	
15GC8-1-4-1		-	-	-	+	-	1
15GC8-1-4-2		-	-	-	-	-	
15GC8-1-4-3		-	-	-	-	-	
15GC8-1-4-4		-	-	-	-	-	
15GC8-1-4-5		+	-	-	-	-	1
15GC8-1-4-7		-	-	-	-	-	
15GC9-1-5-1		+	-	-	-	+	2
15GC9-1-5-2		-	+	-	-	-	1
15GC9-1-5-3		-	+	-	-	-	1
15GC9-1-5-4		+	+	-	+	-	3
15GC11-1-7-1				+	-	-	1
15GC11-1-7-2		-	-	-	-	-	
15GC11-1-7-3		-	-	-	-	-	
15GC11-1-7-4		-	-	-	-	-	
AC2-1-2-6	-	+	-	-	-	-	1
AC2-1-2-7	-	-	-	-	+	-	1
AC2-1-2-10	-	-	-	-	-	+	1
AC3-1-3-2	-	-	-	-	-	+	1
AC3-1-3-3	-	-		-	-	+	1
AC3-1-3-4	-	-	-	-	-	+	1
AC3-1-3-6	-	-	+	-	-	-	1
AC6-1-6-5		+	-	+	-	-	2
AC9-1-4-1	-	-	-	+	-	-	1
AC9-1-4-2		-		-	-	-	
AC9-1-4-3		-		-	-	-	
AC9-1-4-4		-	-	-	-	-	
14C10-1-10-1	-	-	-	-	+	-	1
14C10-1-10-2	-	-	-	-	-	+	1
14C12-1-3-2	-		-	-	-	-	
14C12-1-3-3	-	-	+	-	-	-	1
Affected	0	6	6	3	5	6	
Population	16	38	36	41	40	40	

Supplemental Table S2

F2 Individual Animal Pathology

(A) F2 Generation Glyphosate Males

	Puberty	Testis	Prostate	Kidney	Tumor	Lean	Obese	Total Disease
Rat ID								
15GG1-2-5-8	-	-	+	-	-	-	-	1
15GG1-2-5-9	-	+	-	-	-	-	-	1
15GG1-2-5-10		+	-	+	-	-	-	2
15GG1-2-7-6	-	+	-	-	-	-	-	1
15GG1-2-7-7	-	-	-	-	-	-	+	1
15GG1-2-7-8	-	+	-	-	-	-	-	1
15GG1-2-7-9	-	-	-	-	-	-	-	
15GG1-2-7-10	-	+	-	-	-	-	+	2
15GG2-2-3-6	+	+	-	-	-	-	+	3
15GG2-2-3-7	+	-	-	+	-	+	-	3
15GG2-2-3-8	+	-	-	-	-	-	+	2
15GG2-2-3-9	+	+	-	-	-	-	+	3
15GG4-2-4-6		+	-	-	-	-	+	2
15GG4-2-4-7		-	-	+	-	-	+	2
15GG4-2-4-8		+	-	+	-	-	-	2
15GG4-2-4-9		+	-	-	-	-	+	2
15GG4-2-4-10		-	-	-	-	-	+	1
15GG5-2-1-5		+	+	+	-	-	-	3
15GG5-2-1-6		-	-	+	-	-	-	1
15GG5-2-1-7		+	-	+	-	-	+	3
15GG5-2-2-4		-	-	-	-	-	-	
15GG5-2-2-5		-	-	-	-	-	+	1
15GG5-2-2-7	+	-	+	+	-	-	-	3
15GG5-2-2-8	+	+	-	-	-	+	-	3
15GG5-2-2-9	+	-	-	-	-	-	+	2
15GG5-2-2-10	+	+	+	+	-	-	-	4
15GG6-2-8-2	+	-	-	+	-	-	-	2
15GG6-2-9-4	+	-	-	-	-	-	+	2
Affected	10	14	4	10	0	2	13	
Population	17	28	28	28	28	28	28	

(B) F2 Generation Control Males

	Puberty	Testis	Prostate	Kidney	Tumor	Lean	Obese	Total Disease
Rat ID								
15GC2-2-6-5		-	-	-	-	-	-	
15GC2-2-6-6		-	-	-	-	-	+	1
15GC2-2-6-7		-	+	-	-	+	-	2
15GC2-2-7-3	-	+	-	-	-			1
15GC2-2-7-4		-	-	-	-			
15GC2-2-7-5	-	+	-	-	-			1
15GC3-2-5-8		-	+	-	-	-	-	1
15GC3-2-5-9	-	-	-	+	-	-	-	1
15GC3-2-5-10		-	-	-	-	-	-	
15GC3-2-5-11		+	-	-	-	-	-	1
15GC3-2-5-12	-	-	-	-	-	-	-	
15GC6-2-4-7	-	-	-	-	-	-	-	
15GC6-2-4-8		-	-	-	-	-	-	
15GC6-2-4-9	-	-	-	-	-	-	+	1
15GC6-2-4-10		-	-	-	-	-	-	
15GC10-2-2-10	-	-		-	-	-	-	
15GC10-2-2-11	-	-		-	-	-	-	
15GC10-2-2-12		-		-	-	-	+	1
15GC10-2-2-13		+		+	-	-	-	2
15GC10-2-2-14	-	-		-	-	-	-	
15GC10-2-2-15	-	+		-	-	+	-	2
15GC10-2-2-16	-	-		-	-	-	-	
AC2-2-1-6		+	+	-	-	-	+	3
AC2-2-1-7		+	-	-	-	+	-	2
AC2-2-3-3	-				-	-	-	
AC2-2-10-5	-	-	-	-	-	-	-	
AC2-2-10-6	-	-	-	-	-	+	-	1
AC6-2-2-4		-	-	-	-	-	-	
AC6-2-2-5		-	+	-	-	-	-	1
AC6-2-2-6	+	-	+	-	-	-	+	3
AC6-2-12-6	-	-	-	-	+	-	-	
AC6-2-12-7	-	-	-	-	-	-	-	
AC9-2-6-4	-	-	-	-	-	-	-	
AC9-2-6-5	-	-	-	-	-	-	-	
AC9-2-11-3	-	-	-	-	-	-	+	1
14C10-2-7-4	-					-	-	
14C10-2-7-5	-					-	-	
Affected	1	7	5	2	1	4	6	
Population	22	34	27	34	35	34	34	

(C) F2 Generation Glyphosate Females

	Puberty	Ovary	Kidney	Tumor	Lean	Obese	Total Disease
Rat ID							
15GG1-2-5-3				+	-	+	2
15GG1-2-5-4		+	-	-	-	+	2
15GG1-2-5-5	-	+	-	-	-	-	1
15GG1-2-5-6	-	+	-	-	-	+	2
15GG1-2-5-7	-	+	-	-	-	+	2
15GG1-2-7-2		-	-	-	-	+	1
15GG1-2-7-3		+	-	-	-	+	2
15GG1-2-7-4	-	-	-	-	-	-	
15GG1-2-7-5		-	-	-	-	+	1
15GG2-2-3-2		+	-	-	-	-	1
15GG2-2-3-3		-	-	-	-	+	1
15GG2-2-3-4		-	-	-	-	+	1
15GG2-2-3-5		+	-	-	-	+	2
15GG2-2-6-1		-	-	+	-	-	1
15GG2-2-6-2		+	-	-	-	-	1
14GG4-2-4-1		+	-	-	-	-	1
14GG4-2-4-2		-	+	-	-	-	1
14GG4-2-4-3		-	-	-	-	+	1
14GG4-2-4-4		-	-	-	-	-	
14GG4-2-4-5		-	+	-	-	+	2
15GG5-2-1-1	+	-	-	-	-	+	2
15GG5-2-1-2	+	-	-	-	-	-	1
15GG5-2-1-3	+	-	-	-	-	-	1
15GG5-2-1-4	+	-	-	-	-	-	1
15GG5-2-2-1	+	+	-	-	+	-	3
15GG5-2-2-2	+	+	-	-	-	-	2
15GG5-2-2-3	+	+	+	-	-	-	3
15GG6-2-8-1	-	-	+	-	-	-	1
15GG6-2-9-1		+	-	+	-	-	2
15GG6-2-9-3	-	+	-	-	-	+	2
Affected	7	14	4	3	1	14	
Population	13	29	29	30	30	30	

(D) F2 Generation Control Females

	Puberty	Ovary	Kidney	Tumor	Lean	Obese	Total Disease
Rat ID							
15GC10-2-2-1		+	-	-	+	-	2
15GC10-2-2-2	-	-	+	-	+	-	2
15GC10-2-2-3	-	-	-	+	-	-	1
15GC10-2-2-4	-	-	-	-	-	-	
15GC10-2-2-5	-	-	-	-	-	-	
15GC10-2-2-6	-	-	-	-	-	-	
15GC10-2-2-7	-	-	-	-	-	+	1
15GC10-2-2-8	-	-	-	-	+	-	1
15GC10-2-2-9	-	-	-	-	-	-	
15GC2-2-6-1		+	-	-	-	-	1
15GC2-2-6-2	+	-	-	-	-	-	1
15GC2-2-6-3	+	-	-	-	+	-	2
15GC2-2-6-4		-	-	-	-	-	
15GC2-2-7-1		-	-	-			
15GC2-2-7-2		+	-	-			1
15GC3-2-5-1			+	-			1
15GC3-2-5-2		-	+	-	-	-	1
15GC3-2-5-3		-	-	-	-	-	
15GC3-2-5-4		-	+	-	-	-	1
15GC3-2-5-5		+	-	-	-	-	1
15GC3-2-5-6		-	-	-	-	-	
15GC3-2-5-7		+	-	-	-	-	1
15GC6-2-4-1	-	-	-	-	+	-	1
15GC6-2-4-2		-	-	-	-	+	1
15GC6-2-4-3		-	-	-	-	-	
15GC6-2-4-4		-	-	-	-	-	
15GC6-2-4-5	-	-	-	-	-	-	
15GC6-2-4-6		-	-	-	-	-	
AC2-2-1-1	-	-	+	-	-	-	1
AC2-2-1-3	-	-	-	-	-	-	
AC2-2-1-4	-	-	-	-	-	+	1
AC6-2-2-1	-	+	+	-	-	-	2
AC6-2-2-2	-	-	-	-	-	-	
AC6-2-2-3	-	-	-	-	-	-	
AC2-2-3-1	-	-	-	-	-	+	1
AC2-2-3-2	-	-	-	-	-	-	
AC9-2-6-1		+	+	-	-	-	2
AC9-2-6-2	-	-	-	-	+	-	1
AC9-2-6-3	-	-	-	-	-	+	1
14C10-2-7-1	-	-	-	-	-	-	

14C10-2-7-2	-	-	-	-	-	-	
14C10-2-7-3	-	-	-	-	-	-	
14C10-2-8-1	-		-	-	-	-	
14C10-2-8-2	-	+	-	-	-	-	1
14C10-2-8-3	-	-	-	-	-	+	1
AC9-2-11-1	-	+	-	-	-	-	1
AC9-2-11-2	-	-	-	-	-	-	
AC6-2-12-1	-	-	-	-	-	-	
AC6-2-12-2	-	-	-	-	-	-	
AC6-2-12-3		+	-	-	-	-	1
Affected	2	10	7	1	6	6	
Population	32	48	50	50	47	47	

Supplemental Table S3

F3 Individual Animal Pathology

(A) F3 Generation Glyphosate Males

	Puberty	Testis	Prostate	Kidney	Tumor	Lean	Obese	Total Disease
Rat ID								
15GG1-3-2-7		-	-	-	-	-	+	1
15GG1-3-2-8		-	-	-	-	-	+	1
15GG1-3-2-9		-	+	-	-	-	-	1
15GG1-3-2-10		+	-	-	-	-	-	1
15GG1-3-6-4	-	-	-	-	-	-	+	
15GG1-3-6-5	-	+	-	+	-	-	+	3
15GG1-3-6-6	-	-	-	-	-	-	+	1
15GG1-3-6-7	-	-	-	+	-	-	-	1
15GG1-3-6-8	-	-	+	-	-	-	-	1
15GG2-3-8-3	-	-	-	-	-	-	-	
15GG2-3-8-4	-	-	-	-	-	-	-	
15GG2-3-8-5	-	-	-	-	-	-	+	1
15GG2-3-8-6	-	+	+	-	-	-	-	2
15GG2-3-8-7	-	-	-	+	-	-	-	1
15GG2-3-8-8	-	-	+	-	-	-	-	1
15GG4-3-4-4	-	-	-	-	-	-	+	1
15GG4-3-4-5	-	-	-	+	-	-	-	1
15GG4-3-4-6	-	-	-	-	-	-	-	
15GG4-3-4-7	-	+	-	+	-	-	+	3
15GG4-3-4-8	-	+	+	+	-	-	-	3
15GG4-3-4-9	-	+	-	+	-	-	+	3
15GG4-3-4-10					+			1
15GG4-3-4-11						-	+	1
15GG5-3-1-6		-	-	-	-	-	+	1
15GG5-3-1-7	-	-	-	+	-	-	-	1
15GG5-3-1-8	-	+	-	+	-	-	-	2
15GG5-3-1-9		-	-	-	-	-	+	1
15GG5-3-1-10	-	-	-	-	-	-	-	
15GG5-3-7-6	-	-	+	+	-	-	+	3
15GG5-3-7-7	-	-	-	-	-	-	+	1
15GG5-3-9-4	-	-	+	-	-	-	+	2
15GG5-3-9-5	-	-	+	-	-	-	+	2
15GG6-3-3-6	-	-	-	-	-	-	-	
15GG6-3-3-7	-	+	-	-	-	-	-	1
15GG6-3-3-8	-	-	-	-	-	-	-	
15GG6-3-3-9	-	-	-	-	-	-	+	1
15GG6-3-3-10	-	-	+	-	-	-	-	1
15GG6-3-3-11		-	-	-	-	-	-	
15GG6-3-5-4	-	-	-	-	-	-	-	
15GG6-3-5-5	-	-	-	-	-	-	+	1
15GG6-3-5-6	-	-	+	-	+	-	-	2
15GG6-3-5-7	-	-	+	-	-	-	-	1
15GG6-3-5-8	-	-	+	-	-	-	-	1
15GG6-3-5-9	-	-	-	-	-	-	+	1
15GG6-3-5-10	-	-	-	+	-	-	-	1
15GG6-3-5-11	-	-	+	-	-	-	-	1
Affected	0	8	13	11	2	0	19	
Population	37	44	44	44	45	45	45	

(B) F3 Generation Control Males

	Puberty	Testis	Prostate	Kidney	Tumor	Lean	Obese	Total Disease
Rat ID								
15GC10-3-1-10	-	-	+	-	-	-	-	1
15GC10-3-1-7	+	-	-	-	+	+	-	3
15GC10-3-1-8	+	-	+	-	-	-	-	2
15GC10-3-1-9	-	-	-	-	-	-	-	
15GC3-3-2-7	-	-	-	-	-	-	-	
15GC3-3-2-8	-	-	-	+	-	+	-	2
15GC3-3-3-7	-	+	-	-	-	-	+	2
15GC3-3-3-8	-	-	+	-	-	-	-	1
15GC3-3-3-9	-	-	-	-	-	-	-	
15GC3-3-3-10	-	+	-	-	-	-	+	2
15GC6-3-4-7	-	-	-	-	-	-	-	
15GC6-3-4-8	-	-	-	+	-	-	+	2
15GC6-3-4-9	-	-	-	-	-	-	-	
15GC6-3-7-4		-	-	+	-	-	+	2
15GC6-3-7-5	-	-	-	-	-	-	+	1
AC2-3-3-9	-	-	-	-	-	-	-	
AC2-3-3-10	-					-	-	
AC6-3-1-4	-	-	-	-	-	-	-	
AC6-3-1-5	-	-	-	-	-	-	-	
AC6-3-1-6	-	-	-	-	-	-	-	
AC6-3-6-8	-	-	-	-	-	-	-	
AC6-3-6-9	-	-	-	+	-	-	-	1
AC9-3-4-8	-	-	-	-	-	-	-	
AC9-3-4-9	-	-	-	-	-	-	-	
AC9-3-4-10		-	-	-	-	-	-	
AC9-3-5-8	-	-	-	-	-	-	+	1
AC9-3-5-9	-	-	-	-	-	-	-	
14C10-3-2-8	-	-	-	-	-	-	-	
14C10-3-2-9	-	-	-	-	-	-	-	
14C10-3-2-10	-	-	-	-	-	-	-	
14C10-3-3-8	-	-	-	-	-	-	-	
14C10-3-3-9	-		-	+	-	+	-	2
14C10-3-3-10	-		-	-	-	-	-	
Affected	2	2	3	5	1	3	6	
Population	31	30	32	32	32	33	33	

(C) F3 Generation Glyphosate Females

	Puberty	Ovary	Kidney	Tumor	Lean	Obese	Total Disease
Rat ID							
15GG1-3-2-1	-	+	-	-	-	-	1
15GG1-3-2-2	-	+	-	+	-	+	3
15GG1-3-2-3	-	+	-	+	-	+	3
15GG1-3-2-4		-	-	-	-	-	
15GG1-3-2-5		+	-	-	-	+	2
15GG1-3-2-6		+	-	-	-	-	1
15GG1-3-6-1		+	-	-	+	-	2
15GG1-3-6-2		-	+	-	-	+	2
15GG1-3-6-3		-	-	-	-	+	1
15GG2-3-8-1	-	+	+	-	-	+	3
15GG2-3-8-2	-	+	+	-	-	-	2
15GG4-3-4-1	-	-	-	-			
15GG4-3-4-2	-	-	+	-	-	-	1
15GG4-3-4-3		-	+	-	-	-	1
15GG5-3-1-1		-	+	-	-	+	2
15GG5-3-1-2		+	-	-	-	+	2
15GG5-3-1-3		-	-	-	-	-	
15GG5-3-1-4		+	-	-	-	-	1
15GG5-3-1-5		-	-	-	-	+	1
15GG5-3-7-1	-	-	-	-	-	-	
15GG5-3-7-2		-	-	-			
15GG5-3-7-3	-	-	+	+	-	-	2
15GG5-3-7-4		-	+	-	-	+	2
15GG5-3-7-5	-	+	-	-	-	-	1
15GG5-3-9-1		-		-	-	-	
15GG5-3-9-2		-		-	-	+	1
15GG5-3-9-3		-		-	-	-	
15GG6-3-3-1	-	-	-	-	-	-	
15GG6-3-3-2		-	+	-	-	+	2
15GG6-3-3-3	-	-	+	-	+	-	2
15GG6-3-5-1	-	-	+	-	-	-	1
15GG6-3-5-2	-	-	+	+			2
15GG6-3-5-3	-	+		-	-	-	1
Affected	0	12	12	4	2	12	
Population	15	33	29	33	30	30	

(D) F3 Generation Control Females

	Puberty	Ovary	Kidney	Tumor	Lean	Obese	Total Disease
Rat ID							
15GC10-3-1-2	-	-	-	+			1
15GC10-3-1-3		-	-	-	+	-	1
15GC10-3-1-4	-	+	+	-	+	-	3
15GC10-3-1-5	-	-	-	-	+	-	1
15GC10-3-1-6	-	+	-	-	-	-	1
15GC3-3-2-1		-	+	-	+	-	2
15GC3-3-2-2		-	-	-	-	-	
15GC3-3-2-3		-	+	-	+	-	2
15GC3-3-2-4		-	+	-	-	+	2
15GC3-3-2-5		-	-	-	-	+	1
15GC3-3-2-6	-	+	-	-	+	-	2
15GC3-3-3-1		+		-	-	-	1
15GC3-3-3-3		-	-	-	-	-	
15GC3-3-3-4		-	-	-	+	-	1
15GC3-3-3-5		-	-	-	-	-	
15GC3-3-3-6		-	-	-	+	-	1
15GC6-3-4-1		-		-	-	+	1
15GC6-3-4-2	-	-	-	-	-	-	
15GC6-3-4-3		-	-	-	-	+	1
15GC6-3-4-4		-	-	+	+	-	2
15GC6-3-4-5		-	-	-	+	-	1
15GC6-3-4-6	-	-	-	-	-	-	
15GC6-3-7-1	-	-		-	-	-	
15GC6-3-7-2	-	-	-	-	-	+	1
15GC6-3-7-3	-	-	-	-	-	-	
15GC6-3-7-4	-	-	-	-	-	-	
AC6-3-1-7	-	-	-	-	-	-	
AC6-3-1-8		+	-	-	-	-	1
AC2-3-3-1	-	-	-	-	-	-	
AC2-3-3-2	-	+	-	-	-	-	1
AC2-3-3-3	-	-	-	-	-	-	
AC2-3-3-4	-	-	-	-	-	-	
AC2-3-3-5	-		-	+	-	-	1
AC6-3-6-1		-	-	-	-	-	
AC6-3-6-2		-	-	-	-	-	
AC6-3-6-3		-	-	-	-	-	
AC6-3-6-4		-	-	-	-	-	
AC6-3-6-5		+	-	-	-	-	1
AC2-3-7-1	-	-	-	-	-	-	
AC2-3-7-2		-	-	-	-	-	
AC9-3-5-1	-	-	-	-	-	-	
AC9-3-5-2	-	-	-	-	-	-	

AC9-3-5-3	-	-	-	-	-	-	
AC9-3-5-4	-	-	-	-	-	-	
AC9-3-4-1	-	-	-	-	-	-	
AC9-3-4-2	-	-	-	-	-	-	
AC9-3-4-3	-	-	-	-	-	-	
AC9-3-4-4			-	-	+	-	**1**
14C10-3-2-1	-	-	+	-	-	-	**1**
14C10-3-2-2	-	-	-	-	-	-	
14C10-3-2-3	-	-	-	-	-	-	
14C10-3-2-4	-	+	-	-	-	-	**1**
14C10-3-3-1	-	-	-	-	-	-	
14C10-3-3-2	-	-	-	-	-	-	
14C10-3-3-3	-	-	-	-	-	-	
14C10-3-3-4	-			-	-	-	
Affected	0	8	5	3	11	5	
Population	33	53	52	56	55	55	

Supplemental Table S4
Site Table F1 Generation DMRs p<1e-06

DMR Name	Chr	Start	Length	# Sig Win	min P-value	Exp/ Con Ratio	CpG #	CpG Density	Annotation	Category
DMR1:14808401	1	14808401	500	1	5.90E-07	2.0642	2	0.4	Olig3	Transcription
DMR1:22881501	1	22881501	800	1	1.55E-07	1.6707	14	1.75		
DMR1:29563601	1	29563601	400	1	1.15E-07	0.149	7	1.75		
DMR1:62806601	1	62806601	200	1	6.15E-07	0	0	0	LOC682419	
DMR1:79353601	1	79353601	700	1	9.07E-07	0	1	0.1429	LOC102557244	
DMR1:79516201	1	79516201	700	1	7.04E-07	0.2361	26	3.7143		
DMR1:95086001	1	95086001	1700	1	1.86E-07	2.951	20	1.1765		
DMR1:133679901	1	133679901	200	2	3.18E-09	0.1207	1	0.5		
DMR1:143945801	1	143945801	600	1	9.59E-07	2.1366	11	1.8333		
DMR1:163672001	1	163672001	300	1	9.93E-08	2.421	2	0.6667	Thap12	
DMR1:171333401	1	171333401	500	1	5.56E-07	2.1914	8	1.6	Nlrp14	
DMR1:182463701	1	182463701	1300	1	7.97E-07	1.5394	29	2.2308		
DMR1:186237801	1	186237801	200	1	7.09E-07	0.0173	0	0		
DMR1:192158101	1	192158101	300	1	6.23E-07	1.6606	4	1.3333		
DMR1:201292301	1	201292301	2300	1	3.33E-11	3.5608	60	2.6087	Tacc2	Unknown
DMR1:213567401	1	213567401	200	1	6.48E-08	0	1	0.5	RGD1309350	
DMR1:225255401	1	225255401	900	1	8.29E-08	1.9028	22	2.4444		
DMR1:268129001	1	268129001	1200	1	3.46E-08	1.58	8	0.6667		
DMR1:273940501	1	273940501	400	1	7.44E-07	2.2863	11	2.75	Add3	Cytoskeleton
DMR1:275355901	1	275355901	100	1	8.65E-07	0	0	0		
DMR2:11064401	2	11064401	500	1	2.79E-08	0.2535	9	1.8		
DMR2:25279801	2	25279801	1400	1	2.80E-07	1.5462	19	1.3571		
DMR2:26975901	2	26975901	700	1	2.65E-07	2.5798	15	2.1429		
DMR2:28599401	2	28599401	800	1	3.74E-07	2.0397	8	1	RF00026	
DMR2:77540201	2	77540201	1400	1	6.78E-08	0.3318	7	0.5		
DMR2:77685801	2	77685801	600	1	4.06E-07	1.5951	7	1.1667		
DMR2:159149301	2	159149301	800	1	1.32E-07	2.5983	13	1.625		
DMR2:160647201	2	160647201	700	2	2.68E-07	1.7219	15	2.1429		
DMR2:161034201	2	161034201	1600	1	2.44E-07	1.5672	36	2.25		
DMR2:162203601	2	162203601	1300	1	6.99E-07	1.8197	26	2		
DMR2:165806001	2	165806001	900	2	7.94E-08	1.6251	1	0.1111	RGD1563962;RF00420	Signaling
DMR2:203369601	2	203369601	400	1	3.05E-10	3.3053	3	0.75	Cd101	
DMR2:208204701	2	208204701	500	1	7.39E-08	1.6141	2	0.4	Rap1a	Signaling
DMR2:224665601	2	224665601	200	1	2.09E-07	0	0	0		
DMR2:227444301	2	227444301	7400	11	6.46E-11	1.4553	6	0.0811	Sec24d	Transport
DMR2:249422801	2	249422801	400	1	3.35E-10	0.2689	4	1		
DMR2:258034401	2	258034401	1600	1	6.47E-07	1.8407	17	1.0625	St6galnac5	Metabolism
DMR2:260211901	2	260211901	1200	1	5.40E-07	2.0524	12	1		
DMR3:11174401	3	11174401	500	1	1.66E-07	1.987	22	4.4		
DMR3:36093401	3	36093401	300	1	5.80E-08	0.2825	3	1		
DMR3:41242701	3	41242701	200	1	2.31E-07	0.0287	3	1.5		
DMR3:65081501	3	65081501	600	1	5.52E-07	1.8224	3	0.5		
DMR3:70236001	3	70236001	300	1	9.23E-07	1.7671	15	5		
DMR3:76511401	3	76511401	200	2	2.02E-08	0.0144	0	0	Olr623	Receptor
DMR3:83192101	3	83192101	1000	1	7.66E-07	1.5443	9	0.9	Ttc17	Unknown
DMR3:118675601	3	118675601	400	3	9.26E-10	0.2704	8	2	AABR07053650.1	
DMR3:122627301	3	122627301	300	1	1.97E-07	2.1536	1	0.3333		
DMR3:124509901	3	124509901	100	1	1.83E-07	0.1379	1	1	Prnp	Development
DMR3:125342701	3	125342701	3200	2	8.73E-09	1.8062	63	1.9688	LOC681292	
DMR3:134020101	3	134020101	1500	1	5.07E-07	0.5643	36	2.4		
DMR3:162107601	3	162107601	200	2	1.01E-07	0.2265	1	0.5	Slc13a3;RF00001	Transport
DMR4:7168501	4	7168501	500	1	5.61E-07	2.7004	6	1.2	Asb10;Gbx1	
DMR4:32150901	4	32150901	100	1	8.30E-07	3.3567	0	0		
DMR4:39263801	4	39263801	1300	1	5.27E-07	0.53	11	0.8462		
DMR4:87754001	4	87754001	100	1	3.66E-08	0	0	0	Vom1r73	Receptor
DMR4:87756101	4	87756101	200	2	3.21E-08	0	0	0	Vom1r73	Receptor
DMR4:115939201	4	115939201	500	1	3.11E-07	1.9501	9	1.8		
DMR4:116043001	4	116043001	600	1	1.12E-08	2.0639	7	1.1667		
DMR4:121003701	4	121003701	300	1	4.50E-07	2.6942	12	4	LOC102555094	
DMR4:122483501	4	122483501	300	1	5.84E-07	0.0745	5	1.6667		
DMR4:123570001	4	123570001	1300	3	1.23E-08	1.861	7	0.5385		

DMR4:127463301	4	127463301	1300	2	5.08E-07	0	11	0.8462		
DMR4:129949701	4	129949701	400	2	2.58E-07	2.8767	5	1.25		
DMR4:160837001	4	160837001	600	1	2.32E-07	1.9503	3	0.5		
DMR4:162294301	4	162294301	100	1	1.01E-07	0.0106	0	0	Clec2dl1;Clec2d	Development
DMR4:174460801	4	174460801	500	1	6.60E-09	3.0218	8	1.6		
DMR4:176247901	4	176247901	1000	1	5.51E-07	1.7922	33	3.3	RF00560;Slc21a4	
DMR4:176457201	4	176457201	200	1	4.41E-07	0.4307	3	1.5	Slco1a2;RF00100	Transport
DMR4:179061001	4	179061001	1700	1	2.53E-07	6.2475	18	1.0588	RF00560	
DMR4:179528801	4	179528801	500	1	9.14E-08	2.7736	10	2		
DMR4:182543701	4	182543701	900	2	1.99E-08	1.5623	28	3.1111	Far2	Metabolism
DMR5:6373001	5	6373001	1600	2	1.80E-07	0.3459	24	1.5	AABR07046778.1	
DMR5:24526601	5	24526601	1100	1	2.79E-07	0.0587	18	1.6364	Dpy19l4	Development
DMR5:36441901	5	36441901	200	2	3.05E-08	0	1	0.5	AABR07047528.1	
DMR5:42115501	5	42115501	200	1	7.25E-07	0.1831	0	0		
DMR5:57307101	5	57307101	200	2	8.58E-08	0.3089	2	1	Nfx1	Transcription
DMR5:59996501	5	59996501	2000	1	5.38E-07	1.7436	27	1.35	Pax5	Transcription
DMR5:60795901	5	60795901	800	1	4.12E-08	1.6935	11	1.375		
DMR5:71940901	5	71940901	200	1	1.74E-09	0.3016	3	1.5		
DMR5:79611401	5	79611401	200	1	3.39E-07	0.0207	1	0.5		
DMR5:106888401	5	106888401	400	1	9.04E-07	3.3352	9	2.25		
DMR5:119428601	5	119428601	400	1	2.76E-08	5.1054	4	1		
DMR5:120203401	5	120203401	400	1	4.44E-08	1.9929	4	1		
DMR5:127575801	5	127575801	700	1	8.49E-07	1.4261	1	0.1429	RF00410;Slc1a7	Transport
DMR5:136103301	5	136103301	100	1	2.33E-08	0.1208	2	2	RGD1563714;Tmem53	
DMR5:143484101	5	143484101	100	1	5.12E-08	0.0389	1	1		
DMR5:147652701	5	147652701	600	2	4.55E-09	1.8863	0	0	Bsdc1	
DMR5:151994801	5	151994801	200	2	2.30E-10	0.1689	1	0.5		
DMR5:152423401	5	152423401	600	2	4.79E-07	2.0666	19	3.1667	Umodl;Catsper4	Transport
DMR5:159480301	5	159480301	1300	1	2.53E-07	1.9645	13	1	Padi2;Sdhb	Translation;Metabolism
DMR5:161798601	5	161798601	500	1	4.80E-09	2.2333	17	3.4	Prdm2	Transcription
DMR6:2168101	6	2168101	500	1	3.43E-07	1.6158	4	0.8		
DMR6:2199401	6	2199401	600	2	3.35E-07	2.2352	18	3		
DMR6:6054401	6	6054401	400	1	4.37E-08	0.2754	4	1		
DMR6:6358601	6	6358601	400	1	5.70E-09	1.601	1	0.25		
DMR6:22305001	6	22305001	1600	1	9.52E-07	2.6651	27	1.6875	Dpy30	
DMR6:29175001	6	29175001	800	1	6.91E-07	1.8693	20	2.5	Klhl29	Transcription
DMR6:60378601	6	60378601	2100	1	9.28E-09	5.3489	22	1.0476	Zfp277	Transcription
DMR6:73155401	6	73155401	500	1	4.17E-08	1.5368	2	0.4	Nubpl	Metabolism
DMR6:76342801	6	76342801	800	1	2.44E-07	1.7608	4	0.5	Aldoart2	
DMR6:90935301	6	90935301	600	2	3.37E-08	2.503	3	0.5		
DMR6:108383501	6	108383501	200	1	5.80E-08	0.1135	6	3	RF00026;Syndig1l	
DMR6:128764501	6	128764501	400	1	1.17E-07	2.9654	7	1.75	Glrx5	Metabolism
DMR6:131161301	6	131161301	800	2	1.05E-07	0.0277	12	1.5		
DMR6:137697201	6	137697201	1800	1	4.89E-07	2.1413	30	1.6667		
DMR6:145202701	6	145202701	500	1	9.79E-09	1.6604	1	0.2		
DMR7:1622601	7	1622601	4200	1	6.56E-07	1.6043	223	5.3095	RF00002	
DMR7:11762901	7	11762901	100	1	3.96E-08	0	1	1	Oaz1;Jsrp1	Signaling
DMR7:27290301	7	27290301	1300	1	3.58E-07	1.6289	12	0.9231	LOC362863	Unknown
DMR7:30458601	7	30458601	700	1	8.05E-08	2.8644	18	2.5714	Uhrf1bp1l	Protein Binding
DMR7:51607001	7	51607001	500	1	1.12E-07	1.4268	3	0.6		
DMR7:61301301	7	61301301	200	1	8.95E-08	0.016	1	0.5		
DMR7:64066001	7	64066001	500	1	5.25E-07	2.3123	10	2	Srgap1	Signaling
DMR7:81441001	7	81441001	500	1	5.62E-07	0.2606	1	0.2	Angpt1	Growth Factors & Cytokines
DMR7:108664501	7	108664501	200	1	3.16E-07	0	1	0.5	Phf20l1	Epigenetic
DMR7:118462901	7	118462901	2400	1	5.17E-07	1.892	42	1.75	AABR07058464.1	
DMR7:121045001	7	121045001	300	1	2.59E-07	0.3232	2	0.6667	Cbx6	Transcription
DMR7:127102701	7	127102701	500	1	2.22E-07	1.8955	6	1.2	Tbc1d22a	Signaling
DMR7:130536001	7	130536001	1500	1	3.52E-07	2.1705	35	2.3333	Shank3;Acr	Signaling
DMR7:142410701	7	142410701	300	1	5.48E-07	6.0842	11	3.6667	Slc4a8	Transport
DMR8:4984001	8	4984001	200	1	8.44E-07	0	1	0.5		
DMR8:14000501	8	14000501	400	1	9.15E-07	0	6	1.5		
DMR8:64650601	8	64650601	500	1	4.53E-08	0.0865	6	1.2	Myo9a	Cytoskeleton
DMR8:90794301	8	90794301	1000	1	9.95E-07	0	12	1.2		
DMR8:93781301	8	93781301	400	1	7.87E-07	1.9152	3	0.75		
DMR8:111303401	8	111303401	1700	1	1.48E-07	1.4972	26	1.5294		
DMR8:115004001	8	115004001	800	1	9.73E-07	0.1012	10	1.25	Poc1a	Unknown

DMR8:123942201	8	123942201	1000	1	2.55E-08	1.4919	6	0.6		
DMR8:126642001	8	126642001	1000	1	8.57E-08	0	12	1.2	RF00100	
DMR8:132335901	8	132335901	800	1	2.68E-07	1.8818	3	0.375	Tmem158	Unknown
DMR9:7465701	9	7465701	1300	1	9.29E-07	1.7085	89	6.8462	RF00002	
DMR9:17902401	9	17902401	400	1	7.90E-08	2.9024	7	1.75	Spats1	
DMR9:18166501	9	18166501	500	1	5.75E-07	1.634	2	0.4		
DMR9:21131101	9	21131101	100	1	6.50E-07	0	0	0	Ptchd4	Unknown
DMR9:28290801	9	28290801	200	1	3.86E-07	0	0	0		
DMR9:81253701	9	81253701	100	1	2.12E-07	2.653	4	4	Tns1	Signaling
DMR9:85930001	9	85930001	1100	1	4.79E-08	0.2544	17	1.5455		
DMR9:91255501	9	91255501	1100	1	1.70E-07	3.6037	5	0.4545		
DMR9:93276601	9	93276601	500	2	1.24E-08	1.7934	8	1.6	Armc9	
DMR9:96468301	9	96468301	400	1	5.21E-08	2.3879	6	1.5		
DMR9:97721701	9	97721701	1100	1	4.34E-07	1.9342	19	1.7273		
DMR9:112887101	9	112887101	500	1	1.20E-07	4.1349	4	0.8		
DMR9:113005301	9	113005301	400	2	3.12E-07	2.0462	6	1.5	Tmem232	
DMR10:6418801	10	6418801	200	1	8.04E-07	3.0685	3	1.5		
DMR10:6532701	10	6532701	100	1	9.53E-07	0.1302	4	4		
DMR10:18229701	10	18229701	800	2	2.12E-09	1.7804	16	2	Ranbp17	Binding Protein
DMR10:20357301	10	20357301	1700	1	3.44E-07	2.3119	45	2.6471	Slit3	Development
DMR10:29043201	10	29043201	500	1	2.17E-07	1.8034	4	0.8	Slu7	Transcription
DMR10:35431301	10	35431301	600	1	3.85E-07	2.1538	19	3.1667	Rasgef1c	Signaling
DMR10:56153801	10	56153801	300	1	7.38E-07	0.1807	2	0.6667	Dnah2;Efnb3	Cytoskeleton;Signaling
DMR10:59111001	10	59111001	700	4	1.16E-08	1.8048	16	2.2857	Spns3	Transport
DMR10:71958201	10	71958201	1100	1	2.11E-07	1.9581	17	1.5455		
DMR10:72989801	10	72989801	300	1	2.61E-08	0.3163	3	1		
DMR10:77314701	10	77314701	300	1	2.12E-07	0.3776	0	0		
DMR10:88035401	10	88035401	500	1	2.03E-07	1.8838	12	2.4	Krt13	Cytoskeleton
DMR10:101373901	10	101373901	700	1	7.87E-08	2.0737	18	2.5714		
DMR10:102277001	10	102277001	900	1	4.01E-07	2.331	12	1.3333	Cdc42ep4	Signaling
DMR11:30323201	11	30323201	100	1	3.10E-09	0.0237	0	0		
DMR11:35354101	11	35354101	1000	1	2.12E-08	1.5548	3	0.3		
DMR11:43693201	11	43693201	200	1	5.43E-08	0	0	0	Olr1558;Olr1559	Receptor
DMR11:57349901	11	57349901	900	1	4.13E-08	0	5	0.5556	Phldb2	
DMR11:61977001	11	61977001	1300	1	7.59E-07	2.4135	25	1.9231	Gramd1c;Tigit	Receptor
DMR11:64662901	11	64662901	500	1	1.65E-09	1.8884	10	2	Arhgap31	Signaling
DMR11:74159601	11	74159601	800	1	4.84E-07	2.929	16	2		
DMR12:441101	12	441101	1400	1	8.54E-08	1.9144	56	4		
DMR12:1995801	12	1995801	900	1	1.54E-08	0	22	2.4444	Arhgef18;Pex11g	Signaling
DMR12:13769001	12	13769001	300	1	1.08E-07	2.8871	7	2.3333		
DMR12:14995101	12	14995101	400	1	5.05E-07	2.1701	8	2	AABR07035437.1	
DMR12:15478601	12	15478601	200	1	7.61E-07	0.0303	0	0		
DMR12:22811401	12	22811401	3000	1	7.72E-07	0.0814	49	1.6333	Ift22	
DMR12:24660801	12	24660801	2100	1	2.42E-07	1.4903	14	0.6667	Vps37d;Dnajc30;Bud23	Transcription
DMR12:25707901	12	25707901	1100	1	5.80E-07	2.1235	13	1.1818		
DMR12:29832501	12	29832501	400	1	4.10E-08	3.0752	8	2	Tyw1	Translation
DMR12:33137101	12	33137101	200	2	1.88E-07	0.0904	4	2		
DMR12:37309401	12	37309401	800	1	6.19E-07	1.471	13	1.625		
DMR12:41929801	12	41929801	700	1	5.61E-07	2.4064	8	1.1429	Rbm19	Transcription
DMR12:42020701	12	42020701	400	1	2.71E-07	3.6837	5	1.25	RF00001	
DMR12:43468101	12	43468101	800	1	1.13E-07	2.6545	25	3.125	Med13l	Transcription
DMR12:46341301	12	46341301	3800	1	7.25E-07	2.4399	72	1.8947	Cit	Signaling
DMR12:48316101	12	48316101	1600	2	3.57E-08	1.5281	43	2.6875	Usp30;Svop	Proteolysis;Transport
DMR12:51712601	12	51712601	800	1	1.61E-09	2.8958	8	1	Ttc28	Unknown
DMR13:20086301	13	20086301	100	1	3.81E-08	0	0	0		
DMR13:20177201	13	20177201	300	1	9.90E-08	0.4338	3	1		
DMR13:30815101	13	30815101	200	1	1.61E-07	0.0933	0	0		
DMR13:45327101	13	45327101	400	1	1.82E-07	3.4574	8	2	Cxcr4;AABR07020898.1	Receptor
DMR13:49955001	13	49955001	500	1	8.43E-08	2.7019	9	1.8	AABR07020979.1	
DMR13:70459301	13	70459301	1400	2	3.32E-07	1.7613	16	1.1429	Nmnat2	Metabolism
DMR13:71376301	13	71376301	200	1	9.81E-08	0.0268	2	1		
DMR13:72040101	13	72040101	200	2	4.81E-10	0.1033	2	1	Cacna1e	Transport
DMR13:79666401	13	79666401	200	2	4.08E-11	0.0472	1	0.5	AABR07021589.1	
DMR13:83458201	13	83458201	200	2	3.90E-07	0	5	2.5	Sft2d2	
DMR13:83536701	13	83536701	800	1	2.25E-08	1.7763	16	2	Gpr161	Receptor
DMR13:83566201	13	83566201	1500	1	5.68E-08	1.7145	11	0.7333	Gpr161	Receptor
DMR13:97420801	13	97420801	900	1	2.11E-07	0.0121	15	1.6667		

DMR13:104633701	13	104633701	400	1	2.83E-07	4.0377	11	2.75		
DMR13:108398801	13	108398801	2900	1	4.38E-07	1.5988	50	1.7241	Prox1	Transcription
DMR13:111377201	13	111377201	400	1	1.59E-07	2.217	2	0.5	Hhat	Metabolism
DMR13:112211301	13	112211301	400	1	6.56E-07	2.4723	11	2.75		
DMR14:3304701	14	3304701	1600	1	6.63E-07	1.4687	40	2.5	Btbd8;AC106605.1	
DMR14:4217901	14	4217901	200	1	4.87E-08	0	2	1		
DMR14:22067301	14	22067301	2900	1	8.90E-07	1.7481	83	2.8621	Sult1e1	Metabolism
DMR14:26517901	14	26517901	2800	1	8.16E-07	2.223	83	2.9643		
DMR14:36433901	14	36433901	400	1	2.85E-07	1.5776	6	1.5	Scfd2	Unknown
DMR14:37535001	14	37535001	1800	1	5.57E-08	3.1071	36	2		
DMR14:69971501	14	69971501	200	1	6.74E-07	0.3684	2	1		
DMR14:81384801	14	81384801	400	1	9.05E-07	2.7069	15	3.75	Add1	Cytoskeleton
DMR14:89167701	14	89167701	500	1	1.19E-07	1.7034	5	1	Pkd1l1	Signaling
DMR14:97276601	14	97276601	100	1	3.28E-08	0	0	0		
DMR15:9170601	15	9170601	700	1	3.63E-07	1.4938	5	0.7143		
DMR15:23225401	15	23225401	100	1	3.76E-08	0	0	0		
DMR15:33822601	15	33822601	300	1	1.64E-07	2.7814	14	4.6667	RGD1564324	
DMR15:42766801	15	42766801	500	2	7.99E-08	1.664	3	0.6	Ephx2	Metabolism
DMR15:44340601	15	44340601	1100	1	1.09E-07	0.0201	9	0.8182		
DMR15:47867301	15	47867301	600	1	9.44E-07	1.6895	1	0.1667		
DMR15:55228001	15	55228001	2600	1	9.23E-07	1.5355	21	0.8077		
DMR15:60440301	15	60440301	4200	1	4.57E-07	1.4325	45	1.0714		
DMR15:81511201	15	81511201	400	1	7.16E-07	0.4101	2	0.5		
DMR15:100076301	15	100076301	200	2	8.98E-09	0.0048	0	0		
DMR15:106629601	15	106629601	200	1	1.81E-07	0.3623	0	0	Farp1;Stk24	Signaling
DMR16:24572901	16	24572901	100	1	6.07E-08	0	0	0	Naf1	
DMR16:53012201	16	53012201	300	1	5.47E-09	0.2957	2	0.6667		
DMR16:56833001	16	56833001	200	1	9.29E-07	0	1	0.5	Msr1	Receptor
DMR16:63051101	16	63051101	400	1	9.79E-10	3.1784	5	1.25		
DMR16:68420201	16	68420201	400	1	4.81E-07	1.9521	14	3.5		
DMR16:73185601	16	73185601	1000	1	2.01E-07	1.4421	4	0.4		
DMR16:77234001	16	77234001	500	1	9.53E-07	0.1388	6	1.2		
DMR16:82573001	16	82573001	100	1	5.35E-08	5.2933	4	4	AABR07026544.1	
DMR17:687201	17	687201	100	1	2.50E-07	0.1431	1	1	Npepo	Proteolysis
DMR17:6998501	17	6998501	1100	1	8.19E-08	0.1375	16	1.4545	AABR07026957.1	
DMR17:9711201	17	9711201	1200	1	1.27E-07	0.304	27	2.25	Grk6	Signaling
DMR17:24592201	17	24592201	1400	2	3.69E-07	1.908	20	1.4286		
DMR17:62774301	17	62774301	300	1	1.02E-07	6.3048	4	1.3333	AABR07028262.1;AABR07028266.1	
DMR17:83143701	17	83143701	200	1	1.21E-07	0	0	0		
DMR18:6900801	18	6900801	300	1	4.58E-07	4.6657	5	1.6667	Chst9	Metabolism
DMR18:51927301	18	51927301	300	1	8.60E-09	4.2796	4	1.3333	3-Mar	Metabolism
DMR18:52372001	18	52372001	1800	1	6.23E-07	0.2494	27	1.5	Megf10	Extracellular Matrix
DMR18:67838001	18	67838001	200	1	1.18E-07	0	0	0		
DMR18:71246301	18	71246301	200	1	4.66E-07	0.3448	0	0	Dym	
DMR19:8630701	19	8630701	300	1	1.11E-07	0	0	0		
DMR19:31459101	19	31459101	600	1	2.57E-07	3.2288	6	1		
DMR19:33869401	19	33869401	100	1	4.09E-07	0.1398	0	0	RF00560	
DMR19:49571501	19	49571501	500	1	4.63E-09	2.9105	10	2	Pkd1l2	Signaling
DMR19:53384801	19	53384801	200	1	5.84E-07	2.1261	3	1.5		
DMR19:60219101	19	60219101	1100	2	5.26E-08	1.5885	8	0.7273	Pard3	Cell Junction
DMR20:3632101	20	3632101	2900	2	1.52E-09	1.5018	35	1.2069	AABR07044370.1	
DMR20:4334301	20	4334301	1400	1	1.36E-07	3.3785	33	2.3571	LOC103689965;Notch4	
DMR20:15104701	20	15104701	300	1	2.10E-08	0.0072	0	0	Pcdh15	Extracellular Matrix
DMR20:22953701	20	22953701	1400	1	1.96E-07	1.7938	16	1.1429	Reep3	Receptor
DMR20:30499801	20	30499801	600	1	5.10E-09	0.4312	0	0		
DMR20:34872701	20	34872701	1700	1	4.58E-07	1.7986	33	1.9412	Mcm9	Transcription
DMR20:47411701	20	47411701	400	1	3.38E-07	1.961	12	3	Ostm1	

DMRMT:1	MT	1	16300	4	2.48E-07	0.254	303	1.8589	AY172581.13;AY172581.9;AY172581.3;AY172581.24;AY172581.14;Mt-nd1;AY172581.4;AY172581.21;AY172581.15;Mt-nd2;AY172581.6;AY172581.22;AY172581.18;AY172581.10;AY172581.7;Mt-co1;AY172581.19;AY172581.12;Mt-co2;AY172581.1;Mt-atp8;Mt-atp6;Mt-co3;AY172581.5;Mt-nd3;AY172581.16;Mt-nd4l;Mt-nd4;AY172581.23;AY172581.17;AY172581.11;Mt-nd5;Mt-nd6;AY172581.20;Mt-cyb;AY172581.8;AY172581.2	
DMRX:31179701	X	31179701	300	1	7.54E-07	2.3168	4	1.3333	Mospd2	Development
DMRX:31504101	X	31504101	1400	1	7.24E-07	0	10	0.7143		
DMRX:36223301	X	36223301	200	1	3.38E-07	0.0154	1	0.5		
DMRX:66605001	X	66605001	200	1	1.24E-07	0.0164	0	0	Eda2r	
DMRX:81167501	X	81167501	100	1	5.92E-07	0	0	0		
DMRX:81181401	X	81181401	200	1	2.46E-07	0	0	0		
DMRX:104557901	X	104557901	300	1	1.56E-07	0.1161	2	0.6667		
DMRX:117368601	X	117368601	800	1	6.23E-08	1.5645	7	0.875		
DMRX:120821201	X	120821201	300	2	4.45E-08	0.3703	8	2.6667		
DMRX:133727801	X	133727801	600	1	9.03E-09	0	7	1.1667		
DMRX:151484401	X	151484401	200	2	1.13E-07	0	1	0.5		
DMRY:1527401	Y	1527401	400	1	5.00E-08	2.2259	16	4		

Supplemental Table S5
Site Table F2 Generation DMRs p<1e-06

DMR Name	Chr	Start	Length	# Sig Win	min P-value	Exp/ Con Ratio	CpG #	CpG Density	Annotation	Category
DMR1:72322801	1	72322801	1900	1	2.63E-07	1.791	39	2.0526	U2af2;Ccdc106	Transcription
DMR1:77280801	1	77280801	600	1	8.55E-07	2.082	8	1.3333		
DMR1:78806701	1	78806701	200	1	6.35E-08	0.222	3	1.5	Dact3	
DMR1:100431201	1	100431201	600	1	5.19E-07	2.895	7	1.1667		
DMR1:101416601	1	101416601	1400	1	3.00E-07	2.096	24	1.7143	Ntf4;Lhb;Ruvbl2;Gys1	Signaling;Transcription;Metabolism
DMR1:113770201	1	113770201	200	1	7.89E-07	0.14	0	0		
DMR1:140325401	1	140325401	1000	1	4.31E-07	1.627	21	2.1		
DMR1:184455501	1	184455501	200	1	9.33E-07	3.12	1	0.5		
DMR1:199708301	1	199708301	1600	1	3.30E-08	1.891	12	0.75	RGD1310127	Unknown
DMR1:211959401	1	211959401	300	1	3.86E-08	4.078	2	0.6667	AABR07005983.1	
DMR1:246382201	1	246382201	400	1	5.92E-07	2.195	8	2	Glis3	Transcription
DMR2:120201	2	120201	4600	1	2.76E-07	2.291	183	3.9783		
DMR2:9029101	2	9029101	4100	1	3.47E-07	0.321	34	0.8293	Adgrv1	
DMR2:10801801	2	10801801	400	1	2.69E-08	2.537	3	0.75		
DMR2:15654401	2	15654401	100	1	7.32E-08	5.962	0	0		
DMR2:22052301	2	22052301	800	1	1.90E-07	2.578	27	3.375	Fam151b;Zfyve16	Transport
DMR2:38539701	2	38539701	500	1	1.02E-07	2.367	6	1.2		
DMR2:49657501	2	49657501	1000	1	4.86E-07	2.071	45	4.5		
DMR2:78027401	2	78027401	800	1	2.50E-07	3.029	19	2.375	Myo10	Cytoskeleton
DMR2:118493101	2	118493101	1000	1	8.91E-07	1.806	29	2.9		
DMR2:124002501	2	124002501	1300	1	6.50E-07	0.218	14	1.0769		
DMR2:148944901	2	148944901	900	1	3.71E-07	1.794	27	3		
DMR2:152717301	2	152717301	100	1	9.49E-07	5.455	0	0		
DMR2:225911401	2	225911401	1800	1	2.28E-07	1.457	40	2.2222		
DMR2:238145001	2	238145001	900	1	8.85E-07	2.457	18	2	Gstcd	
DMR2:240758301	2	240758301	300	1	6.60E-07	2.686	6	2	Manba	Golgi
DMR2:252154401	2	252154401	500	1	3.26E-08	1.653	12	2.4	Lpar3	Receptor
DMR2:260875901	2	260875901	600	1	8.31E-07	3.12	12	2	Tyw3;Cryz	Metabolism
DMR3:3912901	3	3912901	2600	1	4.50E-07	2.269	84	3.2308	Notch1	Signaling
DMR3:9610701	3	9610701	500	1	2.31E-07	0.424	0	0		
DMR3:13851801	3	13851801	500	1	4.98E-07	2.35	9	1.8	Hspa5;Rabepk	Signaling
DMR3:20057401	3	20057401	2700	1	8.56E-07	0.309	22	0.8148	AABR07051718.1	
DMR3:27129301	3	27129301	400	1	3.27E-08	0.136	3	0.75	AABR07051908.1	
DMR3:27256401	3	27256401	500	1	9.02E-07	3.114	7	1.4		
DMR3:118675601	3	118675601	400	2	6.98E-09	0.29	8	2	AABR07053650.1	
DMR3:119480301	3	119480301	400	1	2.32E-08	6.213	5	1.25	Ap4e1	Golgi
DMR3:137085101	3	137085101	700	1	3.17E-07	2.377	12	1.7143		
DMR3:153062201	3	153062201	2400	1	6.86E-07	2.284	38	1.5833	RF00283	
DMR3:154266901	3	154266901	400	1	1.44E-07	3.682	9	2.25	Ctnnbl1	
DMR3:162869201	3	162869201	800	1	4.14E-07	3.702	19	2.375	Sulf2	Metabolism
DMR3:168276701	3	168276701	2800	1	1.74E-07	1.494	47	1.6786		
DMR3:173798401	3	173798401	200	1	2.73E-07	4.839	4	2	Phactr3	Signaling
DMR3:175859901	3	175859901	300	1	4.10E-07	5.535	8	2.6667	Slco4a1	Transport
DMR4:76973601	4	76973601	900	1	5.85E-08	0.073	9	1	AABR07060487.1	
DMR4:84640301	4	84640301	1500	1	4.81E-07	3.432	24	1.6	Wipf3	Receptor
DMR4:91736601	4	91736601	600	1	1.71E-07	0.224	2	0.3333	Ccser1	
DMR4:119698401	4	119698401	500	1	9.63E-07	1.807	4	0.8	Rab43	Signaling
DMR4:120648401	4	120648401	700	1	3.59E-07	1.95	13	1.8571		
DMR4:157971601	4	157971601	500	1	7.77E-08	1.91	11	2.2	Cd9	Extracellular Matrix
DMR5:7201	5	7201	600	1	5.22E-07	2.266	21	3.5		
DMR5:22118601	5	22118601	1900	1	9.88E-07	1.889	34	1.7895		
DMR5:61148601	5	61148601	600	1	1.69E-07	4.349	9	1.5		
DMR5:94943201	5	94943201	400	1	7.81E-07	0.299	8	2		
DMR5:136698601	5	136698601	400	1	3.79E-08	4.587	13	3.25	Slc6a9;Ccdc24;B4galt2	Metabolism
DMR5:138450001	5	138450001	1500	1	6.22E-07	3.269	21	1.4	AABR07049790.1	
DMR5:143171501	5	143171501	1200	1	9.43E-07	2.66	18	1.5		
DMR5:163160601	5	163160601	400	1	8.15E-08	5.18	6	1.5	Tnfrsf1b	Apoptosis
DMR5:168777901	5	168777901	2200	1	6.68E-08	2.779	30	1.3636	Camta1	Transcription
DMR6:12326001	6	12326001	300	1	1.22E-07	6.1	5	1.6667	Ppp1r21	
DMR6:28223001	6	28223001	2800	1	3.24E-07	2.398	40	1.4286	Dnmt3a	Transcription
DMR6:42815701	6	42815701	500	1	1.01E-07	2.207	8	1.6		
DMR6:46911001	6	46911001	300	2	2.97E-08	3.226	8	2.6667	RF00560	
DMR6:51067001	6	51067001	400	1	4.83E-07	2.825	4	1	Cog5	Golgi
DMR6:78526101	6	78526101	500	1	5.65E-07	1.776	12	2.4		

DMR6:126228401	6	126228401	100	1	4.61E-07	0.238	0	0	Rin3	Signaling
DMR6:126609401	6	126609401	400	1	9.97E-07	1.954	8	2		
DMR6:127002201	6	127002201	600	1	5.99E-07	2.065	20	3.3333	Unc79;RF02271	
DMR6:127673301	6	127673301	400	1	3.40E-07	2.513	6	1.5	Serpina9	Proteolysis
DMR6:132656501	6	132656501	1800	1	5.50E-07	0.199	12	0.6667		
DMR6:134502501	6	134502501	900	1	2.78E-07	4.24	26	2.8889		
DMR7:6471401	7	6471401	400	1	3.74E-07	2.658	18	4.5		
DMR7:14224201	7	14224201	2600	1	4.00E-07	2.473	73	2.8077	Ephx3;Brd4	Metabolism;Transcription
DMR7:21357701	7	21357701	2000	1	8.34E-07	0.243	11	0.55		
DMR7:66505701	7	66505701	700	1	4.55E-07	2.119	19	2.7143		
DMR7:69618501	7	69618501	100	1	6.07E-07	0.128	0	0		
DMR7:73275601	7	73275601	1000	1	5.04E-07	2.351	16	1.6	Rida;Pop1	Transcription
DMR7:142252601	7	142252601	1100	1	5.73E-07	2.228	23	2.0909	Dazap2;Smagp	
DMR8:6181801	8	6181801	200	1	1.03E-07	7.794	1	0.5	Yap1	Transcription
DMR8:7978401	8	7978401	400	1	3.43E-07	2.993	7	1.75	Cntn5	Extracellular Matrix
DMR8:13067101	8	13067101	3700	1	4.55E-07	2.834	62	1.6757	Amotl1	Unknown
DMR8:72419601	8	72419601	700	1	8.25E-07	3.136	28	4	Car12	Metabolism
DMR8:82177201	8	82177201	800	1	5.09E-08	2.149	9	1.125	Myo5c	Cytoskeleton
DMR8:111392201	8	111392201	300	1	2.05E-08	0.41	2	0.6667	Ryk	Receptor
DMR8:115073801	8	115073801	500	1	1.49E-07	2.245	23	4.6	Dusp7	Signaling
DMR8:116339001	8	116339001	400	1	2.12E-09	2.268	14	3.5	Hyal3;Hyal1;Nat6;Ifrd2;Lsmem2	Receptor;Metabolism
DMR8:119126501	8	119126501	500	1	3.61E-09	2.212	11	2.2	Prss50;Prss45;Prss46	Protease
DMR8:126109101	8	126109101	500	1	5.03E-07	2.811	14	2.8		
DMR9:15614901	9	15614901	600	1	1.62E-07	1.726	10	1.6667	RF00026;Guca1a;Guca1b	Signaling
DMR9:16894501	9	16894501	1400	1	6.86E-07	7.622	21	1.5	Ttbk1	Signaling
DMR9:30030101	9	30030101	800	1	6.19E-07	0.278	3	0.375	B3gat2	Metabolism
DMR9:81405001	9	81405001	400	1	7.49E-07	3.2	7	1.75	Tns1;AABR07068074.2;Rufy4	Signaling
DMR9:105027101	9	105027101	1000	1	1.70E-07	0.218	13	1.3		
DMR10:3022901	10	3022901	700	1	9.86E-07	2.797	11	1.5714		
DMR10:37992301	10	37992301	1200	1	2.63E-07	2.001	18	1.5		
DMR10:56063301	10	56063301	1600	1	1.70E-07	3.792	16	1	Dnah2	Cytoskeleton
DMR10:72071701	10	72071701	200	1	1.01E-07	2.56	2	1		
DMR10:97222801	10	97222801	3100	1	9.57E-08	3.353	77	2.4839	Axin2	Signaling
DMR10:100797901	10	100797901	500	1	1.19E-08	5.054	14	2.8		
DMR10:101604301	10	101604301	700	1	7.89E-07	7.05	17	2.4286		
DMR10:102533401	10	102533401	300	1	6.51E-07	0.45	3	1	Sdk2	Development
DMR10:106332401	10	106332401	500	1	9.90E-07	2.583	7	1.4	9-Sep	Cytoskeleton
DMR11:13977501	11	13977501	300	1	8.18E-07	0.355	3	1	Lipi	
DMR11:35566201	11	35566201	500	1	2.80E-07	2.509	5	1	Kcnj15	Transport
DMR11:44538201	11	44538201	600	1	2.32E-07	2.101	15	2.5		
DMR11:53610401	11	53610401	700	1	7.08E-08	2.127	15	2.1429		
DMR11:69451401	11	69451401	1100	1	3.54E-07	3.097	24	2.1818		
DMR12:6437601	12	6437601	1400	1	6.55E-07	2.58	45	3.2143	B3glct	
DMR12:11284101	12	11284101	500	1	9.06E-09	2.643	13	2.6	Arpc1a	Cytoskeleton
DMR12:11907801	12	11907801	400	1	1.96E-07	1.977	5	1.25		
DMR12:25733001	12	25733001	400	1	7.37E-07	2.386	8	2		
DMR12:30385601	12	30385601	4200	1	1.13E-07	2.568	2	0.0476		
DMR12:31081601	12	31081601	5500	2	1.30E-08	1.961	3	0.0545		
DMR12:38163501	12	38163501	3400	1	2.06E-07	1.876	67	1.9706	Hcar2	
DMR12:43150801	12	43150801	1200	1	5.70E-08	2.162	18	1.5		
DMR12:44665701	12	44665701	1000	1	3.55E-07	2.54	20	2	Ksr2	Signaling
DMR12:47571901	12	47571901	800	1	2.64E-07	2.051	29	3.625	Ankrd13a	
DMR12:51312901	12	51312901	400	1	6.93E-07	1.75	5	1.25	Pitpnb	
DMR13:19630001	13	19630001	1100	1	5.38E-08	0.259	23	2.0909		
DMR13:68428501	13	68428501	400	1	6.82E-07	2.149	8	2		
DMR13:69458701	13	69458701	700	1	5.48E-07	2.352	13	1.8571		
DMR13:77807101	13	77807101	1100	1	2.00E-07	1.818	17	1.5455	AABR07021544.1	
DMR13:82580001	13	82580001	2900	1	3.03E-08	2.15	31	1.069	Ccdc181;Blzf1	Transcription
DMR13:83536701	13	83536701	900	1	7.62E-07	1.652	17	1.8889	Gpr161	Receptor
DMR13:97421001	13	97421001	600	1	2.08E-07	0.029	10	1.6667		
DMR13:98257201	13	98257201	1100	1	2.45E-07	3.525	20	1.8182	Cdc42bpa	Signaling
DMR13:105548801	13	105548801	500	1	6.08E-08	6.151	12	2.4	Spata17	
DMR13:109470201	13	109470201	1400	1	3.82E-08	2.82	16	1.1429	Rps6kc1	Signaling
DMR14:11319201	14	11319201	900	1	6.57E-07	1.995	19	2.1111	AC131411.1;AC131411.2	
DMR14:17943701	14	17943701	200	1	6.98E-08	3.577	3	1.5		
DMR14:46643401	14	46643401	7500	1	7.34E-07	1.392	899	11.9867	RF01518;Rn5-8s;AABR07015078.1;AABR07015078.2;AABR07015079.1;AABR07015080.2;AABR07015081.2;AABR07015080.1;AABR07015081.1	

DMR14:62491301	14	62491301	300	2	2.35E-08	0.213	0	0		
DMR14:85361401	14	85361401	2600	1	2.59E-08	1.895	48	1.8462	Rhbdd3;Emid1	Extracellular Matrix
DMR14:101849601	14	101849601	3000	1	7.36E-07	0.315	59	1.9667		
DMR14:104836101	14	104836101	200	1	1.84E-07	0.301	12	6	Sertad2	Transcription
DMR15:47983001	15	47983001	2700	1	3.34E-07	2.836	26	0.963		
DMR15:104436301	15	104436301	400	1	8.45E-07	3.993	7	1.75	AABR07019412.1	
DMR15:107955301	15	107955301	700	1	5.02E-07	1.851	21	3	Dock9	Cell Cycle
DMR16:4204301	16	4204301	500	1	8.91E-08	2.939	13	2.6		
DMR16:13318301	16	13318301	100	1	1.91E-07	0.123	0	0		
DMR16:47188601	16	47188601	800	1	4.08E-07	3.149	16	2	Dctd	
DMR16:69361101	16	69361101	700	1	4.80E-07	2.258	23	3.2857		
DMR16:83968401	16	83968401	800	1	9.42E-08	1.649	8	1		
DMR17:14462301	17	14462301	2200	1	5.58E-08	3.013	35	1.5909	AABR07027111.1	
DMR17:29883601	17	29883601	300	1	2.82E-07	4.318	4	1.3333	Cdyl	Metabolism
DMR17:34625701	17	34625701	300	1	5.03E-07	2.837	5	1.6667		
DMR17:56409801	17	56409801	200	1	7.31E-07	3.803	3	1.5		
DMR17:78196801	17	78196801	200	1	2.41E-09	2.344	4	2		
DMR17:90064101	17	90064101	500	1	3.37E-07	0.124	1	0.2	Pdss1;RF00406	Metabolism
DMR18:25109101	18	25109101	1400	1	8.31E-07	2.893	10	0.7143		
DMR18:52663101	18	52663101	900	1	4.07E-07	5.004	19	2.1111		
DMR18:60691501	18	60691501	200	1	1.55E-07	1.494	1	0.5	Nedd4l	Protease
DMR18:65427001	18	65427001	900	1	2.57E-07	2.91	18	2	Tcf4	Transcription
DMR18:74336101	18	74336101	1400	1	4.18E-07	1.836	31	2.2143	Epg5	
DMR18:80092601	18	80092601	1300	2	2.03E-08	1.68	4	0.3077		
DMR19:11578001	19	11578001	2000	1	8.43E-07	2.485	32	1.6	Gnao1	Signaling
DMR19:25337901	19	25337901	1900	1	7.40E-07	2.398	43	2.2632	Zswim4	Transcription
DMR19:28208801	19	28208801	1100	1	3.36E-08	0.185	11	1		
DMR19:31772401	19	31772401	500	1	4.14E-07	2.94	11	2.2		
DMR19:32755401	19	32755401	400	1	1.29E-07	0.352	7	1.75		
DMR19:49241301	19	49241301	500	1	7.36E-07	2.841	4	0.8		
DMR19:50785401	19	50785401	200	1	1.16E-07	3.196	4	2		
DMR19:51577501	19	51577501	1100	1	7.90E-07	1.963	22	2	Cdh13	Extracellular Matrix
DMR20:4584001	20	4584001	1200	1	9.09E-08	3.654	56	4.6667	Zbtb12;Ehmt2;Slc44a4	Transcription;Metabolism
DMR20:12006501	20	12006501	700	1	7.49E-07	3.314	19	2.7143	Adarb1	Epigenetic
DMR20:14061201	20	14061201	500	1	9.56E-07	2.654	13	2.6	Lrrc75b	
DMR20:22261501	20	22261501	600	2	4.55E-07	2.247	25	4.1667	AABR07044820.1	
DMR20:32047801	20	32047801	1500	1	9.23E-08	3.402	35	2.3333	AABR07045032.1;Supv3l1	Transcription
DMR20:35671301	20	35671301	1100	1	1.03E-07	0.099	8	0.7273		
DMR20:42769101	20	42769101	500	1	7.31E-07	3.983	10	2	AABR07045319.1	
DMRX:63598001	X	63598001	4600	1	5.85E-07	1.841	19	0.413	Apoo	Extracellular Matrix
DMRX:81181501	X	81181501	100	1	7.10E-07	0	0	0		
DMRY:1452901	Y	1452901	1300	1	2.51E-08	2.097	38	2.9231		

Supplemental Table S6
Site Table F3 Generation DMRs p<1e-06

DMR Name	Chr	Start	Length	# Sig Win	min P-value	Exp/ Con Ratio	CpG #	CpG Density	Annotation	Category
DMR1:773701	1	773701	500	1	3.24E-07	0.48	10	2		
DMR1:6685501	1	6685501	500	2	2.32E-08	1.529	2	0.4	Utrn	Cytoskeleton
DMR1:11903501	1	11903501	12300	1	4.94E-08	0.528	1401	11.3902	RF00002;RF01518	
DMR1:11924501	1	11924501	1400	1	1.53E-07	1.636	21	1.5	RF01518	
DMR1:11962001	1	11962001	14200	2	1.50E-08	0.491	1494	10.5211	RF01518;AABR07000398.1;RF00002;AABR07000402.1;AABR07000404.1;LOC10090959	
DMR1:13485401	1	13485401	2100	1	9.44E-07	1.688	42	2		
DMR1:21773001	1	21773001	1100	1	2.67E-07	1.636	1	0.0909	Enpp1	Signaling
DMR1:22677801	1	22677801	1800	1	3.89E-07	0.257	81	4.5		
DMR1:24547301	1	24547301	300	1	7.62E-07	0.429	5	1.6667		
DMR1:26469701	1	26469701	300	1	8.88E-07	1.679	3	1		
DMR1:26565201	1	26565201	1600	1	5.11E-08	2.167	12	0.75		
DMR1:33368301	1	33368301	800	1	7.50E-07	1.467	5	0.625		
DMR1:38442401	1	38442401	3700	1	3.19E-07	1.411	56	1.5135		
DMR1:43865101	1	43865101	200	1	2.07E-08	1.766	0	0	Cnksr3	Signaling
DMR1:45440901	1	45440901	400	1	5.78E-09	2.203	1	0.25		
DMR1:53670001	1	53670001	1000	1	8.33E-07	2.681	6	0.6		
DMR1:54423001	1	54423001	4800	1	2.00E-11	0.617	58	1.2083		
DMR1:70613801	1	70613801	300	1	5.87E-07	1.991	5	1.6667		
DMR1:80429801	1	80429801	900	1	5.70E-07	0.347	21	2.3333	Trappc6a;Nkpd1;Ppp1r37	Metabolism
DMR1:83519901	1	83519901	500	1	3.05E-08	2.539	0	0	Cyp2b12	Metabolism
DMR1:83834001	1	83834001	900	1	2.35E-07	1.804	5	0.5556		
DMR1:87061301	1	87061301	700	1	3.25E-07	1.757	4	0.5714	Capn12	Protease
DMR1:88820101	1	88820101	3400	1	1.14E-08	0.365	53	1.5588	Lrfn3;RF00004	Receptor
DMR1:92061201	1	92061201	1300	1	7.75E-07	0.492	37	2.8462	Dpy19l3	Development
DMR1:93885401	1	93885401	1500	1	7.93E-08	0.279	19	1.2667	Zfp536	Transcription
DMR1:96331701	1	96331701	600	1	7.09E-07	1.518	2	0.3333		
DMR1:143481101	1	143481101	300	1	1.32E-07	1.491	0	0	Homer2	Signaling
DMR1:147112401	1	147112401	1700	1	8.71E-11	0.495	130	7.6471		
DMR1:161286801	1	161286801	600	1	1.13E-07	0.344	5	0.8333	AABR07004852.1	
DMR1:161748001	1	161748001	700	1	6.82E-07	0.35	16	2.2857	Tenm4	Signaling
DMR1:173467701	1	173467701	100	1	8.42E-07	5.617	0	0	Olr281	Receptor
DMR1:173805901	1	173805901	3700	1	7.82E-07	1.367	51	1.3784		
DMR1:176415901	1	176415901	300	1	3.24E-07	1.686	1	0.3333	Galnt18	Unknown
DMR1:182837501	1	182837501	2600	1	1.42E-07	2.409	30	1.1538	AABR07005416.1;Ssty1	
DMR1:192839501	1	192839501	1100	1	6.98E-07	3.926	3	0.2727		
DMR1:201859301	1	201859301	500	1	3.16E-07	2.2	3	0.6	AC123083.1	
DMR1:213528201	1	213528201	400	1	5.06E-08	2.048	0	0	Cyp2e1;Syce1	Metabolism
DMR1:221336401	1	221336401	400	1	4.08E-07	1.522	0	0	Slc22a20;Capn1	Transport;Protease
DMR1:223731001	1	223731001	4700	1	1.82E-08	0.37	57	1.2128		
DMR1:240706601	1	240706601	200	1	2.38E-07	0.185	8	4	Trpm3	Receptor
DMR1:244850501	1	244850501	500	1	5.06E-08	1.613	1	0.2		
DMR1:249595501	1	249595501	1400	1	2.24E-07	1.773	15	1.0714		
DMR1:257403401	1	257403401	400	1	9.75E-07	0.276	11	2.75	Plce1	Signaling
DMR1:260430301	1	260430301	1100	1	1.54E-07	0.41	26	2.3636	Tll2	Protease
DMR1:269146001	1	269146001	300	1	6.91E-07	0.181	5	1.6667		
DMR1:273800601	1	273800601	1000	1	1.06E-07	2.136	3	0.3		
DMR1:281015501	1	281015501	600	1	2.41E-08	1.603	0	0		
DMR2:269301	2	269301	800	1	5.29E-07	0.423	24	3		
DMR2:12498501	2	12498501	1300	1	2.67E-07	2.468	4	0.3077		
DMR2:18122801	2	18122801	600	1	3.28E-08	2.684	6	1	Edil3	Extracellular Matrix
DMR2:26359301	2	26359301	100	1	6.38E-07	5.901	0	0	Iqgap2	Signaling
DMR2:29742901	2	29742901	600	2	1.24E-07	1.689	1	0.1667	Map1b	Cytoskeleton
DMR2:54711101	2	54711101	1400	1	2.12E-07	1.483	12	0.8571	C7	Immune
DMR2:77620501	2	77620501	400	1	8.80E-07	0.381	8	2	Basp1	
DMR2:128814901	2	128814901	4800	1	8.21E-07	0.478	255	5.3125	RF00002	
DMR2:156979201	2	156979201	21100	1	2.67E-07	0.416	811	3.8436		
DMR2:165233401	2	165233401	1500	1	9.98E-09	0.461	49	3.2667		
DMR2:178343601	2	178343601	600	1	3.93E-07	2.204	2	0.3333	Fnip2;AC121415.2	
DMR2:195030801	2	195030801	900	1	5.87E-07	2.273	1	0.1111	AABR07012426.1	
DMR2:198329701	2	198329701	500	1	2.24E-07	0.511	8	1.6	Sv2a;Bola1	Development
DMR2:201701201	2	201701201	300	1	1.79E-08	2.903	3	1		
DMR2:207706801	2	207706801	600	1	4.88E-08	1.802	0	0		
DMR2:213051201	2	213051201	700	1	8.42E-08	2.352	1	0.1429		

DMR2:215418901	2	215418901	600	1	1.76E-07	1.886	3	0.5		
DMR2:225061601	2	225061601	700	1	9.37E-07	0.388	9	1.2857	Slc44a3;AABR07013154.1	Metabolism
DMR2:236372001	2	236372001	800	1	9.97E-09	1.744	6	0.75	Hadh	Metabolism
DMR2:252443801	2	252443801	2400	1	9.47E-07	0.158	38	1.5833	Dnase2b;Uox	Transcription
DMR2:260855401	2	260855401	600	1	1.89E-07	2.024	0	0	Tyw3	
DMR2:262997701	2	262997701	200	1	9.02E-07	2.255	4	2	Negr1	Growth Factors & Cytokines
DMR3:8082001	3	8082001	600	1	1.55E-07	1.494	2	0.3333		
DMR3:9289801	3	9289801	800	1	6.48E-07	0.336	16	2	Lamc3	Extracellular Matrix
DMR3:38318401	3	38318401	300	1	7.30E-08	1.44	1	0.3333		
DMR3:60362001	3	60362001	1600	1	5.35E-09	1.817	18	1.125	RF00001	
DMR3:63144701	3	63144701	1200	1	2.00E-09	2.552	7	0.5833	Cyct	
DMR3:80960201	3	80960201	400	1	9.36E-07	3.082	2	0.5	AC135645.2	
DMR3:102221201	3	102221201	200	1	1.47E-07	2.078	1	0.5		
DMR3:117494601	3	117494601	500	1	2.06E-07	1.826	0	0	Slc12a1	Transport
DMR3:118527001	3	118527001	1400	1	1.28E-07	1.991	19	1.3571		
DMR3:121740801	3	121740801	500	2	1.41E-07	0.32	7	1.4	Slc20a1	Metabolism
DMR3:121939801	3	121939801	1300	1	7.39E-07	0.36	35	2.6923		
DMR3:124492801	3	124492801	600	1	2.59E-07	1.989	6	1		
DMR3:124769001	3	124769001	600	1	7.00E-07	0.371	20	3.3333		
DMR3:135369301	3	135369301	1100	1	4.47E-07	2.553	6	0.5455		
DMR3:153044501	3	153044501	700	1	8.17E-07	2.335	5	0.7143	Ndrg3	Transcription
DMR3:164466501	3	164466501	700	1	2.01E-07	2.36	6	0.8571		
DMR3:166106601	3	166106601	400	1	2.95E-08	1.934	3	0.75		
DMR3:169649101	3	169649101	400	1	5.59E-08	0.301	14	3.5	AABR07054801.1	
DMR3:169745301	3	169745301	1400	1	3.11E-08	5.502	19	1.3571		
DMR3:174105101	3	174105101	400	1	4.35E-07	0.302	12	3		
DMR3:176937901	3	176937901	900	1	1.85E-08	1.935	1	0.1111	Zbtb46;RF00026	Transcription
DMR4:4782901	4	4782901	300	1	1.17E-07	1.784	2	0.6667		
DMR4:5643001	4	5643001	800	1	2.91E-07	1.953	6	0.75	Actr3b	Cytoskeleton
DMR4:9717901	4	9717901	400	1	6.83E-07	0.483	12	3	Reln	Protease
DMR4:25755101	4	25755101	600	2	2.11E-07	1.581	3	0.5		
DMR4:31254401	4	31254401	1200	1	4.55E-09	0.417	35	2.9167	AABR07059663.1	
DMR4:33054601	4	33054601	500	1	1.70E-07	0.233	13	2.6		
DMR4:45947001	4	45947001	200	1	4.23E-07	3.859	1	0.5		
DMR4:48455001	4	48455001	400	1	1.84E-07	0.486	12	3		
DMR4:59957001	4	59957001	500	1	1.64E-07	0.301	11	2.2		
DMR4:77408001	4	77408001	4300	1	8.66E-08	1.438	98	2.2791	AABR07060519.1	
DMR4:77413601	4	77413601	1000	2	1.64E-07	1.448	13	1.3	AABR07060519.1	
DMR4:80607301	4	80607301	300	1	4.14E-07	2.151	3	1		
DMR4:82035201	4	82035201	1400	1	1.01E-07	1.545	0	0		
DMR4:118576401	4	118576401	600	1	7.48E-07	1.721	2	0.3333	Anxa4	Binding Protein
DMR4:119145201	4	119145201	1200	1	1.86E-07	1.834	13	1.0833	Gkn1	
DMR4:119180901	4	119180901	1100	1	3.01E-07	2.183	1	0.0909	Gkn2;Gkn3	
DMR4:127464001	4	127464001	600	1	2.44E-08	2.186	6	1		
DMR4:130474101	4	130474101	500	1	3.94E-08	1.772	4	0.8		
DMR4:145610801	4	145610801	300	1	4.13E-07	0.254	9	3	Irak2	Receptor
DMR4:166666201	4	166666201	3100	1	3.72E-09	0.544	169	5.4516	RF00002	
DMR4:178058001	4	178058001	700	1	2.72E-07	2.223	10	1.4286	Sox5	Transcription
DMR4:178065401	4	178065401	2200	1	9.75E-07	0.267	62	2.8182	Sox5	Transcription
DMR5:16330501	5	16330501	3200	1	8.31E-07	0.475	97	3.0312		
DMR5:27070301	5	27070301	1800	3	4.67E-09	1.642	10	0.5556		
DMR5:27389301	5	27389301	300	1	2.69E-07	1.538	2	0.6667	Runx1t1	Transcription
DMR5:44965101	5	44965101	800	1	8.44E-08	4.87	2	0.25		
DMR5:46735501	5	46735501	300	1	1.53E-07	2.561	1	0.3333		
DMR5:56849001	5	56849001	1100	1	4.55E-07	0.213	15	1.3636		
DMR5:58381201	5	58381201	800	1	3.12E-07	0.146	28	3.5	Phf24	
DMR5:58844701	5	58844701	1100	1	5.48E-07	1.816	14	1.2727	Rusc2	
DMR5:79000201	5	79000201	300	1	2.33E-07	2.378	1	0.3333	Kif12	Cytoskeleton
DMR5:84276301	5	84276301	300	1	7.56E-07	0.201	4	1.3333		
DMR5:91123801	5	91123801	14200	1	7.40E-07	0.426	1605	11.3028	RF01518;RF00002;AABR07048791.1	
DMR5:93806601	5	93806601	400	1	4.45E-07	7.333	4	1	AABR07048856.1	
DMR5:107335401	5	107335401	300	1	8.52E-07	2.816	1	0.3333	Klhl9	Transcription
DMR5:141613901	5	141613901	1000	1	3.42E-07	1.731	6	0.6		
DMR5:149313601	5	149313601	300	1	4.87E-07	2.058	0	0		
DMR5:149684401	5	149684401	400	1	2.21E-07	1.868	2	0.5	AABR07050061.1	
DMR5:151058701	5	151058701	400	1	2.67E-07	0.416	10	2.5	Stx12	Transport
DMR5:153078601	5	153078601	900	1	4.40E-07	2.008	11	1.2222		
DMR5:154051801	5	154051801	600	1	1.92E-07	1.605	2	0.3333	Ifnlr1	Receptor
DMR5:166409301	5	166409301	2500	1	5.88E-08	0.478	59	2.36	Nmnat1	Metabolism

DMR5:167681101	5	167681101	900	1	5.69E-07	0.462	34	3.7778	Slc45a1	Transport
DMR5:169559101	5	169559101	400	1	2.00E-07	0.291	15	3.75	Chd5	Transcription
DMR5:171146901	5	171146901	900	1	5.72E-07	0.35	15	1.6667	RF00560	
DMR5:171761001	5	171761001	300	1	9.84E-07	1.416	0	0		
DMR6:3469901	6	3469901	3100	1	7.77E-09	2.009	12	0.3871		
DMR6:10465901	6	10465901	1900	2	3.60E-07	0.479	85	4.4737		
DMR6:10805801	6	10805801	9900	1	6.71E-09	0.328	426	4.303		
DMR6:14859601	6	14859601	200	1	2.80E-07	4.77	1	0.5	Nrxn1	Receptor
DMR6:21445501	6	21445501	800	1	6.13E-08	0.367	15	1.875	Ltbp1	Metabolism
DMR6:24196401	6	24196401	400	2	1.16E-07	1.805	2	0.5	AABR07063248.1	
DMR6:30628001	6	30628001	7400	1	1.92E-10	0.313	788	10.6486	RF00002;AABR07063421.1;AABR07063424.1;AABR07063425.3;AABR07063425.2;AABR07063425.1	
DMR6:30638101	6	30638101	1100	2	3.47E-09	0.534	113	10.2727	RF00002;AABR07063421.1;AABR07063424.1;AABR07063425.3;AABR07063425.2;AABR07063425.1	
DMR6:31062601	6	31062601	3700	1	2.08E-07	1.831	68	1.8378		
DMR6:41997201	6	41997201	400	1	6.91E-07	0.197	10	2.5	Greb1	
DMR6:43304801	6	43304801	200	1	7.61E-07	4.323	5	2.5	Asap2	Development
DMR6:100611801	6	100611801	600	1	7.05E-07	0.539	11	1.8333		
DMR6:103939401	6	103939401	1700	1	2.33E-07	3.753	12	0.7059	LOC500684;AABR07065012.1	Translation
DMR6:107010601	6	107010601	600	1	1.11E-07	0.369	16	2.6667	Dcaf4	
DMR6:108431201	6	108431201	400	1	3.64E-07	2.117	6	1.5		
DMR6:128247801	6	128247801	600	1	9.11E-08	2.515	2	0.3333		
DMR6:134183901	6	134183901	200	1	6.73E-07	0.312	9	4.5		
DMR6:134844001	6	134844001	1600	1	2.11E-07	0.172	50	3.125	Ppp2r5c	Signaling
DMR6:134973201	6	134973201	2500	1	6.80E-07	0.279	89	3.56	Dync1h1	Cytoskeleton
DMR6:145543401	6	145543401	400	1	1.53E-07	0.191	5	1.25	Rapgef5	Development
DMR6:145926101	6	145926101	300	1	6.76E-07	3.642	17	5.6667	Dnah11	Cytoskeleton
DMR6:146250601	6	146250601	1600	1	2.22E-11	4.875	29	1.8125		
DMR7:710101	7	710101	4400	2	5.03E-09	0.572	254	5.7727	RF00002	
DMR7:732401	7	732401	2900	1	2.25E-08	0.399	116	4		
DMR7:1621201	7	1621201	10700	2	4.13E-07	0.581	334	3.1215	RF00002	
DMR7:1673001	7	1673001	5500	1	5.16E-08	0.573	328	5.9636		
DMR7:2220101	7	2220101	3300	1	6.73E-08	0.484	117	3.5455	RF00002	
DMR7:12384701	7	12384701	2700	1	2.38E-07	0.388	46	1.7037	Efna2	Signaling
DMR7:16630501	7	16630501	200	1	1.59E-07	2.334	1	0.5		
DMR7:22452301	7	22452301	2900	2	8.57E-08	0.52	131	4.5172	RF00002	
DMR7:24058301	7	24058301	2100	2	2.09E-09	1.656	47	2.2381	Btbd11	Signaling
DMR7:25870701	7	25870701	1400	1	3.18E-07	1.839	25	1.7857	Rfx4	Transcription
DMR7:32478801	7	32478801	400	1	9.77E-07	1.956	0	0	AABR07056616.1;RF00001	
DMR7:54513101	7	54513101	200	1	2.37E-07	2.161	0	0		
DMR7:62713901	7	62713901	1600	1	4.52E-08	2.035	17	1.0625		
DMR7:62894501	7	62894501	600	1	1.22E-07	0.41	14	2.3333	Msrb3	Metabolism
DMR7:72581601	7	72581601	900	1	7.99E-08	0.396	14	1.5556		
DMR7:75438701	7	75438701	300	1	2.94E-07	2.749	4	1.3333		
DMR7:82819301	7	82819301	600	1	2.60E-07	1.794	3	0.5		
DMR7:96702701	7	96702701	500	2	6.41E-10	1.463	1	0.2		
DMR7:99269501	7	99269501	1700	1	6.88E-08	1.858	7	0.4118		
DMR7:117236501	7	117236501	5900	1	9.81E-07	0.344	342	5.7966	Plec	Cytoskeleton
DMR7:130333101	7	130333101	300	1	3.12E-07	1.479	2	0.6667	Lmf2;Ncaph2;Tymp	Translation;Metabolism
DMR7:135823401	7	135823401	400	1	7.15E-07	0.484	12	3	Irak4	Signaling
DMR7:136200601	7	136200601	500	1	9.29E-07	0.315	13	2.6	Tmem117	Unknown
DMR7:142251801	7	142251801	1900	1	6.98E-07	0.27	38	2	Dazap2;Smagp	
DMR8:10458701	8	10458701	1400	1	1.08E-07	0.31	14	1		
DMR8:13505601	8	13505601	300	1	9.34E-07	1.978	6	2	LOC108348070	
DMR8:14457301	8	14457301	300	1	3.77E-07	3.047	3	1	Fat3	Transcription
DMR8:36322201	8	36322201	300	1	2.38E-07	0.185	12	4	St3gal4;Dcps	Metabolism
DMR8:47314001	8	47314001	1400	1	5.30E-07	0.451	33	2.3571	Arhgef12	Signaling
DMR8:51020501	8	51020501	200	1	4.36E-07	2.504	0	0		
DMR8:52753901	8	52753901	1400	1	8.49E-07	2.104	12	0.8571	Nxpe1	
DMR8:54439901	8	54439901	500	1	1.01E-07	1.807	3	0.6		
DMR8:59384401	8	59384401	700	1	1.05E-08	1.989	3	0.4286		
DMR8:62228901	8	62228901	200	1	2.80E-07	2.212	1	0.5	Scamp5	Metabolism
DMR8:62371001	8	62371001	1400	1	5.16E-07	0.386	27	1.9286	Scamp2;Ulk3;Cplx3	Binding Protein;Development;Metabolism
DMR8:68416701	8	68416701	100	1	6.27E-07	0.166	3	3	Iqch	

DMR8:72801301	8	72801301	300	1	1.82E-08	2.263	6	2		
DMR8:75345401	8	75345401	200	1	3.21E-07	2.383	2	1		
DMR8:121474601	8	121474601	700	1	5.46E-08	1.675	1	0.1429		
DMR8:124241701	8	124241701	600	1	2.11E-07	1.481	2	0.3333		
DMR8:126981701	8	126981701	1800	1	2.63E-07	1.75	10	0.5556	AABR07071701.1	
DMR8:127483901	8	127483901	1300	1	6.12E-08	0.338	32	2.4615	Itga9	Extracellular Matrix
DMR8:128895301	8	128895301	400	1	4.26E-07	1.722	2	0.5	AABR07071745.1	
DMR9:1649301	9	1649301	200	1	8.67E-07	0.421	13	6.5	Tbc1d5	Signaling
DMR9:3732501	9	3732501	800	1	5.40E-07	1.453	5	0.625		
DMR9:11934201	9	11934201	500	1	2.35E-07	1.932	7	1.4		
DMR9:18053901	9	18053901	400	1	3.21E-07	0.295	10	2.5		
DMR9:19377701	9	19377701	2000	1	3.63E-07	1.572	18	0.9	Clic5	Transport
DMR9:20527501	9	20527501	1400	1	9.55E-07	1.7	11	0.7857	AABR07066871.2;AABR07066871.3	
DMR9:21334601	9	21334601	300	1	2.04E-11	1.887	2	0.6667	Ptchd4	Unknown
DMR9:37552801	9	37552801	400	1	1.17E-08	1.896	0	0		
DMR9:41220101	9	41220101	1100	2	3.57E-07	2.049	7	0.6364		
DMR9:42127701	9	42127701	400	1	4.50E-07	0.25	6	1.5		
DMR9:42506901	9	42506901	1900	1	3.47E-07	1.342	27	1.4211		
DMR9:45953801	9	45953801	700	1	8.56E-08	1.716	8	1.1429	Npas2	Transcription
DMR9:54612901	9	54612901	1600	1	9.84E-07	0.404	30	1.875	Myo1b	Cytoskeleton
DMR9:62623501	9	62623501	300	1	3.28E-07	2.332	3	1		
DMR9:79873501	9	79873501	400	1	4.38E-08	1.844	1	0.25	4-Mar	Metabolism
DMR9:81453401	9	81453401	500	1	3.34E-07	0.354	16	3.2		
DMR9:98353901	9	98353901	1600	1	7.56E-07	1.742	15	0.9375	Ramp1	Signaling
DMR9:99648301	9	99648301	400	1	3.28E-09	0.129	10	2.5	Ndufa10l1;Olr1343	Metabolism;Receptor
DMR9:102810801	9	102810801	900	1	1.25E-10	1.86	9	1		
DMR9:104915101	9	104915101	600	1	8.75E-08	1.694	1	0.1667		
DMR9:110664601	9	110664601	400	1	9.81E-07	0.352	14	3.5	Fbxl17	Proteolysis
DMR10:5314901	10	5314901	2700	1	7.40E-07	1.712	49	1.8148	Tvp23a	
DMR10:29115601	10	29115601	100	1	9.75E-07	3.511	0	0	Ccnjl	Signaling
DMR10:31609001	10	31609001	300	1	7.46E-07	3.247	2	0.6667	LOC100359777	
DMR10:45457801	10	45457801	1100	1	2.62E-07	1.628	14	1.2727	Obscn	Unknown
DMR10:45913001	10	45913001	200	1	4.12E-07	3.381	1	0.5	Nlrp3;Olr1462	Unknown
DMR10:54022701	10	54022701	400	1	8.94E-07	0.284	12	3		
DMR10:55817201	10	55817201	800	1	9.39E-07	0.425	14	1.75		
DMR10:56142401	10	56142401	1000	1	2.58E-07	1.644	9	0.9	Dnah2	Cytoskeleton
DMR10:68253301	10	68253301	800	1	2.90E-07	1.536	9	1.125	Asic2	Transport
DMR10:68992201	10	68992201	600	1	1.63E-07	0.285	17	2.8333		
DMR10:76850301	10	76850301	900	1	6.66E-07	1.515	2	0.2222		
DMR10:83007601	10	83007601	700	1	3.11E-08	1.534	2	0.2857		
DMR10:84050801	10	84050801	500	1	1.58E-07	1.465	2	0.4		
DMR10:84522701	10	84522701	300	1	6.45E-07	2.596	2	0.6667	Skap1	
DMR10:89809001	10	89809001	1200	1	1.95E-07	2.14	26	2.1667	Meox1	Transcription
DMR10:96506501	10	96506501	700	1	2.18E-07	0.363	15	2.1429	Prkca	Binding Protein
DMR10:101093801	10	101093801	1400	1	4.77E-09	1.561	4	0.2857		
DMR10:101564301	10	101564301	800	1	8.18E-07	0.532	22	2.75		
DMR10:107961301	10	107961301	600	1	5.91E-07	1.697	2	0.3333		
DMR10:108125401	10	108125401	1200	1	2.91E-07	0.339	41	3.4167	Cbx2	Transcription
DMR11:12598201	11	12598201	300	1	1.84E-07	10.363	3	1		
DMR11:29958101	11	29958101	800	1	9.58E-07	2.157	19	2.375	Tiam1	Transcription
DMR11:35545501	11	35545501	1400	1	2.78E-07	2.546	34	2.4286		
DMR11:37207001	11	37207001	400	1	1.56E-07	1.656	0	0	Dscam	Extracellular Matrix
DMR11:45363101	11	45363101	800	1	1.36E-07	1.426	14	1.75	Tmem30c	Cell Cycle
DMR11:62107001	11	62107001	300	1	7.46E-07	1.59	1	0.3333	Gramd1c;Zbtb20	Transcription
DMR11:62135101	11	62135101	200	1	4.61E-07	0.236	9	4.5	Gramd1c;Zbtb20	Transcription
DMR11:64396401	11	64396401	700	1	2.67E-07	2.41	18	2.5714	Igsf11;RF00026	Extracellular Matrix
DMR11:70000901	11	70000901	1500	1	9.32E-07	1.508	13	0.8667	Kalrn	Signaling
DMR11:73399201	11	73399201	700	1	2.53E-07	1.433	2	0.2857		
DMR11:79777101	11	79777101	400	1	3.06E-09	4.893	11	2.75		
DMR12:5618801	12	5618801	1000	1	2.58E-07	0.257	16	1.6	Fry	Development
DMR12:10323901	12	10323901	300	1	1.65E-07	2.29	0	0	Wasf3	Cytoskeleton
DMR12:16751701	12	16751701	800	1	9.37E-07	0.386	41	5.125	Elfn1	Receptor
DMR12:22454001	12	22454001	400	2	4.95E-07	0.435	12	3	Slc12a9;Trip6	Transport;Cytoskeleton
DMR12:25813601	12	25813601	700	1	1.71E-07	1.456	2	0.2857		
DMR12:27147401	12	27147401	700	1	7.98E-08	1.656	2	0.2857	AABR07035916.1	
DMR12:29455601	12	29455601	500	1	9.24E-07	1.884	0	0	Caln1	Signaling
DMR12:29706201	12	29706201	300	1	8.44E-07	1.962	8	2.6667	Caln1	Signaling
DMR12:34708301	12	34708301	300	1	9.07E-07	2.787	3	1		
DMR12:37483801	12	37483801	2000	1	5.39E-08	0.207	19	0.95	Tmed2;Rilpl1	Metabolism;Transcription

DMR12:37496201	12	37496201	400	1	7.46E-07	0.197	11	2.75	Rilpl1	Transcription
DMR12:39212301	12	39212301	600	2	1.89E-08	0.324	18	3	Anapc5	
DMR12:46239801	12	46239801	3200	2	1.73E-08	0.285	37	1.1562	RF00100	
DMR12:48847001	12	48847001	900	1	1.67E-07	1.56	9	1	Wscd2	
DMR13:2515101	13	2515101	2500	1	9.25E-07	0.355	99	3.96		
DMR13:32137701	13	32137701	1900	1	5.44E-07	0.417	78	4.1053		
DMR13:35165701	13	35165701	2300	1	6.75E-07	1.695	30	1.3043		
DMR13:44541801	13	44541801	700	1	4.38E-07	1.588	2	0.2857	AABR07020879.1	
DMR13:45623201	13	45623201	300	1	4.84E-07	1.804	3	1		
DMR13:48015801	13	48015801	400	1	1.01E-07	1.935	5	1.25	Rassf5	
DMR13:52035001	13	52035001	900	1	3.37E-07	7.643	8	0.8889		
DMR13:53100501	13	53100501	400	1	6.57E-08	0.33	14	3.5	RGD1311892	
DMR13:60152101	13	60152101	300	2	2.24E-10	1.717	2	0.6667		
DMR13:68530301	13	68530301	300	1	5.70E-07	0.328	4	1.3333		
DMR13:70521301	13	70521301	600	1	5.95E-07	2.245	5	0.8333	Nmnat2	Metabolism
DMR13:80356101	13	80356101	500	1	3.63E-08	0.076	8	1.6	Dnm3	Cytoskeleton
DMR13:89293301	13	89293301	400	1	1.97E-08	0.346	14	3.5	Fcrlb;Fcrla	Receptor
DMR13:96263301	13	96263301	1200	1	2.01E-07	0.347	21	1.75		
DMR13:104476501	13	104476501	300	1	5.51E-07	0.391	6	2	AABR07022053.1	
DMR13:106575301	13	106575301	700	1	3.02E-08	1.55	7	1	Esrrg	Receptor
DMR13:106940401	13	106940401	400	1	2.37E-07	2.539	3	0.75	Ush2a	Extracellular Matrix
DMR13:108572401	13	108572401	2600	1	1.33E-07	3.223	33	1.2692	AABR07022113.1	
DMR14:8254201	14	8254201	1300	1	2.33E-07	2.346	15	1.1538	Mapk10	Signaling
DMR14:8968801	14	8968801	1400	1	4.34E-07	0.368	61	4.3571		
DMR14:11686101	14	11686101	800	1	6.48E-07	0.337	29	3.625		
DMR14:14677101	14	14677101	300	1	1.44E-07	0.26	4	1.3333	Fras1	Development
DMR14:17349201	14	17349201	1200	1	1.94E-07	1.688	10	0.8333		
DMR14:35819301	14	35819301	1000	1	5.57E-07	1.376	6	0.6		
DMR14:36882401	14	36882401	400	1	2.92E-07	2.012	1	0.25	AABR07014897.1	
DMR14:37621401	14	37621401	200	1	5.37E-07	5.633	3	1.5	Fryl	
DMR14:42884901	14	42884901	900	1	7.52E-07	0.411	18	2	Limch1	Cytoskeleton
DMR14:46522201	14	46522201	9000	3	1.14E-07	0.976	680	7.5556	AABR07015055.1;AABR07015057.1;AABR07015055.2;AABR07015056.1	
DMR14:46532501	14	46532501	6600	1	1.67E-08	1.481	85	1.2879	AABR07015055.1;AABR07015057.1;AABR07015055.2;AABR07015056.1	
DMR14:46540101	14	46540101	1300	1	1.44E-07	1.919	20	1.5385		
DMR14:46548101	14	46548101	8000	2	8.46E-08	0.792	126	1.575		
DMR14:46588301	14	46588301	800	1	1.86E-08	0.463	80	10	RF01518;AABR07015066.1;AABR07015067.1;AABR07015067.2	
DMR14:46590101	14	46590101	15400	3	9.12E-08	0.819	385	2.5	RF01518;AABR07015066.1;AABR07015067.1;AABR07015067.2	
DMR14:46619801	14	46619801	1600	1	9.49E-07	1.307	19	1.1875		
DMR14:46636901	14	46636901	5400	1	1.49E-10	0.525	600	11.1111	RF01518;Rn5-8s;AABR07015078.1;AABR07015078.2	
DMR14:46643301	14	46643301	15500	7	1.08E-08	0.486	1496	9.6516	RF01518;Rn5-8s;AABR07015078.1;AABR07015078.2;AABR07015079.1;AABR07015080.2;AABR07015081.2;AABR07015080.1;AABR07015081.1	
DMR14:46660201	14	46660201	1500	1	1.69E-07	1.561	23	1.5333	AABR07015078.1;AABR07015078.2;AABR07015079.1;AABR07015080.2;AABR07015081.2;AABR07015080.1;AABR07015081.1	
DMR14:46679001	14	46679001	9500	1	4.59E-07	0.345	1044	10.9895	RF01518;RF00002	
DMR14:60462101	14	60462101	400	1	9.49E-07	0.294	9	2.25	Anapc4	Cell Cycle
DMR14:70007701	14	70007701	200	1	8.51E-08	3.638	1	0.5	Fam184b	
DMR14:105548001	14	105548001	600	1	1.97E-07	1.876	1	0.1667		
DMR15:5392701	15	5392701	700	2	1.50E-07	1.967	1	0.1429		
DMR15:5436301	15	5436301	800	1	2.30E-10	2.166	1	0.125	Spetex-2F;AABR07016950.2	Unknown
DMR15:9138901	15	9138901	400	1	1.85E-07	2.87	4	1		
DMR15:23851001	15	23851001	1000	1	4.80E-07	1.638	4	0.4	Samd4a	Signaling
DMR15:28779401	15	28779401	400	1	4.99E-07	0.244	32	8	Olr1639	
DMR15:42827401	15	42827401	100	1	2.75E-07	15.438	1	1	Chrna2;Ptk2b	Receptor;Signaling

DMR15:45368001	15	45368001	600	1	2.02E-08	0.332	12	2	Dleu7	
DMR15:52736001	15	52736001	600	1	4.16E-09	1.436	0	0		
DMR15:56301201	15	56301201	900	1	1.66E-07	0.247	27	3		
DMR15:59583901	15	59583901	2000	1	3.38E-08	0.499	154	7.7		
DMR15:65440701	15	65440701	500	1	4.78E-07	2.009	3	0.6		
DMR15:85819901	15	85819901	300	1	6.61E-07	0.37	5	1.6667		
DMR15:104154601	15	104154601	600	1	1.96E-07	0.445	17	2.8333	Dzip1	Transcription
DMR16:5676301	16	5676301	800	1	6.25E-07	1.491	6	0.75	Cacna2d3	Transport
DMR16:9600301	16	9600301	1000	1	7.62E-07	0.428	29	2.9	Arhgap22	Unknown
DMR16:15614601	16	15614601	400	1	6.58E-08	2.264	2	0.5		
DMR16:15627201	16	15627201	1300	1	6.20E-07	2.053	26	2		
DMR16:32181401	16	32181401	500	1	7.51E-08	1.817	3	0.6	Sh3rf1	
DMR16:45437101	16	45437101	300	1	1.63E-07	2.524	1	0.3333		
DMR16:73488501	16	73488501	700	1	2.06E-07	1.961	2	0.2857		
DMR16:81584001	16	81584001	1200	1	6.44E-07	2.695	3	0.25	LOC103693999;NEWGENE_1582994;RF00026	
DMR16:84591901	16	84591901	400	1	2.30E-07	1.645	1	0.25	Myo16	Cytoskeleton
DMR17:6916801	17	6916801	600	1	8.44E-07	1.72	8	1.3333	LOC681410	Transcription
DMR17:13855801	17	13855801	1600	1	9.65E-07	0.357	21	1.3125	RF00017	
DMR17:17851201	17	17851201	800	1	3.74E-07	0.275	19	2.375	Rnf144b	Proteolysis
DMR17:17900601	17	17900601	400	1	1.81E-07	1.895	9	2.25	Rnf144b	Proteolysis
DMR17:20789201	17	20789201	3500	1	2.14E-07	1.413	29	0.8286		
DMR17:22557301	17	22557301	300	1	4.43E-07	1.824	0	0		
DMR17:37677701	17	37677701	400	1	7.51E-07	4.296	5	1.25	RGD1563300;AABR07027581.1	
DMR17:75670701	17	75670701	500	1	5.29E-07	2.082	3	0.6		
DMR17:79094501	17	79094501	2700	1	3.52E-07	0.313	85	3.1481	Fam171a1	
DMR18:12279201	18	12279201	1100	1	1.66E-07	4.977	15	1.3636	AABR07031399.1	
DMR18:12752901	18	12752901	700	1	1.10E-07	1.815	11	1.5714		
DMR18:15459101	18	15459101	500	1	4.62E-07	0.215	11	2.2	B4galt6	Golgi
DMR18:25274101	18	25274101	1000	2	4.34E-07	1.446	16	1.6	AABR07031674.1	
DMR18:34247701	18	34247701	300	1	3.76E-07	4.236	1	0.3333		
DMR18:53970401	18	53970401	2400	1	4.22E-08	1.568	24	1	Adamts19	Proteolysis
DMR18:61745701	18	61745701	600	1	6.98E-07	1.553	3	0.5		
DMR18:64606101	18	64606101	1900	1	7.49E-07	2.219	17	0.8947		
DMR18:69962001	18	69962001	1200	1	4.21E-07	8.331	12	1		
DMR18:70495301	18	70495301	100	1	3.61E-07	7.928	1	1	Myo5b	Cytoskeleton
DMR18:84832101	18	84832101	200	1	4.44E-08	2.966	0	0		
DMR19:249701	19	249701	300	1	1.22E-07	3.268	1	0.3333	AABR07042607.1	
DMR19:5829301	19	5829301	8600	1	6.57E-07	0.746	230	2.6744		
DMR19:18155901	19	18155901	1200	3	1.17E-07	1.763	11	0.9167	Tox3	Transcription
DMR19:24925901	19	24925901	600	1	5.21E-09	3.209	9	1.5		
DMR19:25337801	19	25337801	400	1	9.02E-07	0.422	14	3.5	Zswim4	Transcription
DMR19:27994001	19	27994001	600	2	7.37E-09	2.114	5	0.8333	AABR07043389.1	
DMR19:39448501	19	39448501	1100	1	3.05E-07	2.506	18	1.6364		
DMR19:42763101	19	42763101	3600	1	3.33E-07	0.2	144	4	Zfhx3	Transcription
DMR19:42792801	19	42792801	1000	1	5.44E-07	0.149	23	2.3	Zfhx3	Transcription
DMR19:52477001	19	52477001	600	1	9.10E-07	1.38	3	0.5	Cotl1	Cytoskeleton
DMR19:53268901	19	53268901	400	1	4.14E-07	2.172	3	0.75		
DMR19:54125601	19	54125601	600	1	7.76E-09	0.348	29	4.8333	Gse1	Transcription
DMR19:54996901	19	54996901	2000	2	2.42E-09	0.554	36	1.8		
DMR19:56971701	19	56971701	1300	1	5.81E-07	1.648	8	0.6154	Cox6c-ps1	
DMR19:57172201	19	57172201	400	1	2.16E-07	0.316	9	2.25	Pgbd5	Epigenetic
DMR19:59156801	19	59156801	1000	1	1.44E-07	2.231	11	1.1		
DMR20:5072201	20	5072201	300	1	5.21E-07	3.146	5	1.6667	Ly6g6d;Ly6g6e;Ly6g6f;AC094348.1;Abhd16a	Immune
DMR20:8345601	20	8345601	1500	1	5.55E-07	0.136	31	2.0667	Cmtr1	Epigenetic
DMR20:18691201	20	18691201	600	1	4.33E-07	2.336	5	0.8333		
DMR20:22907101	20	22907101	1100	1	6.80E-08	0.421	31	2.8182	Reep3	Receptor
DMR20:28121201	20	28121201	300	1	5.58E-07	0.368	4	1.3333	Ccdc138	
DMR20:32797901	20	32797901	600	2	2.21E-08	1.53	0	0	AABR07045047.1	
DMR20:40504901	20	40504901	800	1	4.01E-07	1.764	8	1	Hs3st5;AABR07045274.1	Metabolism
DMR20:47411301	20	47411301	600	1	9.37E-07	0.39	14	2.3333	Ostm1	
DMRX:182201	X	182201	400	1	7.80E-08	4.072	1	0.25		
DMRX:32237801	X	32237801	300	1	2.38E-08	2.506	2	0.6667	RF00026;Ca5b	
DMRX:36466401	X	36466401	5200	1	1.76E-07	0.11	108	2.0769		
DMRX:73399601	X	73399601	2000	2	4.21E-08	2.075	0	0	Zfp449	Transcription
